# Derivation of induced pluripotent stem cells from orangutan skin fibroblasts

**DOI:** 10.1186/s13104-015-1567-0

**Published:** 2015-10-16

**Authors:** Krishna Ramaswamy, Wing Yan Yik, Xiao-Ming Wang, Erin N. Oliphant, Wange Lu, Darryl Shibata, Oliver A. Ryder, Joseph G. Hacia

**Affiliations:** Department of Biochemistry and Molecular Biology, University of Southern California, Los Angeles, CA USA; Department of Preventive Medicine, University of Southern California, Los Angeles, CA USA; San Diego Zoo Institute for Conservation Research , San Diego Zoo Global, San Diego, CA USA

**Keywords:** Induced pluripotent stem cell, Reprogramming, Conservation, Evolution, Orangutan, Great ape, Gene expression

## Abstract

**Background:**

Orangutans are an endangered species whose natural habitats are restricted to the Southeast Asian islands of Borneo and Sumatra. Along with the African great apes, orangutans are among the closest living relatives to humans. For potential species conservation and functional genomics studies, we derived induced pluripotent stem cells (iPSCs) from cryopreserved somatic cells obtained from captive orangutans.

**Results:**

Primary skin fibroblasts from two Sumatran orangutans were transduced with retroviral vectors expressing the human *OCT4*, *SOX2*, *KLF4*, and c-*MYC* factors. Candidate orangutan iPSCs were characterized by global gene expression and DNA copy number analysis. All were consistent with pluripotency and provided no evidence of large genomic insertions or deletions. In addition, orangutan iPSCs were capable of producing cells derived from all three germ layers in vitro through embryoid body differentiation assays and in vivo through teratoma formation in immune-compromised mice.

**Conclusions:**

We demonstrate that orangutan skin fibroblasts are capable of being reprogrammed into iPSCs with hallmark molecular signatures and differentiation potential. We suggest that reprogramming orangutan somatic cells in genome resource banks could provide new opportunities for advancing assisted reproductive technologies relevant for species conservation efforts. Furthermore, orangutan iPSCs could have applications for investigating the phenotypic relevance of genomic changes that occurred in the human, African great ape, and/or orangutan lineages. This provides opportunities for orangutan cell culture models that would otherwise be impossible to develop from living donors due to the invasive nature of the procedures required for obtaining primary cells.

**Electronic supplementary material:**

The online version of this article (doi:10.1186/s13104-015-1567-0) contains supplementary material, which is available to authorized users.

## Background

Orangutans are southeast Asian great apes that last shared a common ancestor with humans and the African great apes (chimpanzees, bonobos, and gorillas) 12–16 million year ago [1]. The native geographical distribution of the two orangutan species is currently restricted to the South-East Asian islands of Borneo (*Pongo pygmaeus*) and Sumatra (*Pongo abelii*) [2]. Their speciation time is estimated to be approximately 300,000–400,000 years ago, with evidence of continued low levels of gene flow afterwards [1, 3]. Both orangutan species are classified as endangered [4, 5] with an estimated 6600 Sumatran and 54,000 Bornean individuals alive in 2004 [6]. In general, orangutan census techniques are subject to interpretation since they are most commonly based on the density of orangutan nests due to the fact that direct counts of animals are hindered by low encounter rates [7]. Nevertheless, the indisputable rapid decline in Sumatran and Bornean orangutan populations (suggested average annual losses of 230–400 Sumatran and 2050–4850 Bornean orangutans over the last 25 years) has heightened awareness that rapid action is needed for conversation efforts to be effective [5, 8–10].

The ability to reprogram somatic cells obtained from living or recently deceased donors into induced pluripotent stem cells (iPSCs) provides exciting new opportunities for conversation biology [11]. While reprogramming technologies are constantly evolving, traditionally this has been accomplished through the transient expression of a group of transcription factors (*OCT4*, *SOX2*, *KLF4*, and *c*-*MYC*), also called Yamanaka reprogramming factors, in cultured somatic cells. iPSCs can be expanded indefinitely and differentiated into cells from all three germ layers. This is highlighted by ongoing efforts to develop methods to differentiate iPSCs from endangered species into germ cells for the in vitro production of embryos that can be implanted into surrogate females of a related existing species [12]. This would further enhance the value of cryopreserved cells banks, such as the Frozen Zoo© at the Zoological Society of San Diego [13, 14]. The initial reports of iPSCs derived from endangered species involved the silver-maned drill monkey and northern white rhinoceros [15] followed by the prairie vole [16] and snow leopard [17]. Most recently, iPSCs have been reported for small numbers of chimpanzees, bonobos, and gorillas using traditional Yamanaka reprogramming factors and retroviral vectors that integrated into the genome [18, 19]. iPSCs has also been derived from two chimpanzees using reprogramming methods that do not involve the genomic integration of transgenes [20]. Despite these reports involving African great apes as well as non-endangered primates used in biomedical research [21], the derivation of iPSCs from orangutans has not been reported to date.

In addition to the aforementioned conservation efforts, orangutans have also been studied because of their complex behaviors [22] and their phylogenetic relationships with humans and the African great apes [23]. While the social behaviors [24–26] and adept tool use [26–32] of orangutans have been extensively studied, it remains a challenge to catalog the underlying genetic underpinnings for the cognitive abilities and other specializations in the orangutan lineage. Genomic analyses have indicated positive selection in the orangutan lineage of genes that play critical roles in pathways pertaining to visual perception and glycolipid metabolism relevant to neurological functions [1]. Nevertheless, it is difficult to test hypotheses regarding the functional relevance of these genomic signatures of selection given the limited availability of appropriately preserved orangutan tissues and cultured cells outside of primary skin fibroblasts [33] and transformed lymphoblasts [34]. A recent investigation of enhancer divergence and cis-regulatory evolution in the human and chimp neural crest using iPSC-derived cell culture models highlights the value these models can have in addressing fundamental questions in molecular evolution [35]. Furthermore, more complex organoid models, such as ‘mini-brains’, that can be developed from iPSCs [36] would open new opportunities to engage in comparative neuroscience studies.

Here, we report the generation of orangutan iPSCs as a novel resource for conservation biology and investigating the functional relevance of genetic changes that could have contributed to phenotypic specializations in great apes. Gene expression information obtained from these orangutan iPSCs as part of their characterization could provide information relevant to molecular changes that have occurred after the split of the human and orangutan lineages. Furthermore, these resources provide an initial step required for the development of in vitro model systems to investigate potential differences in the production and activities of specialized cell types in humans and the great apes.

## Results and discussion

### Derivation of candidate iPSCs from primary orangutan skin fibroblasts

**Fig. 1:**
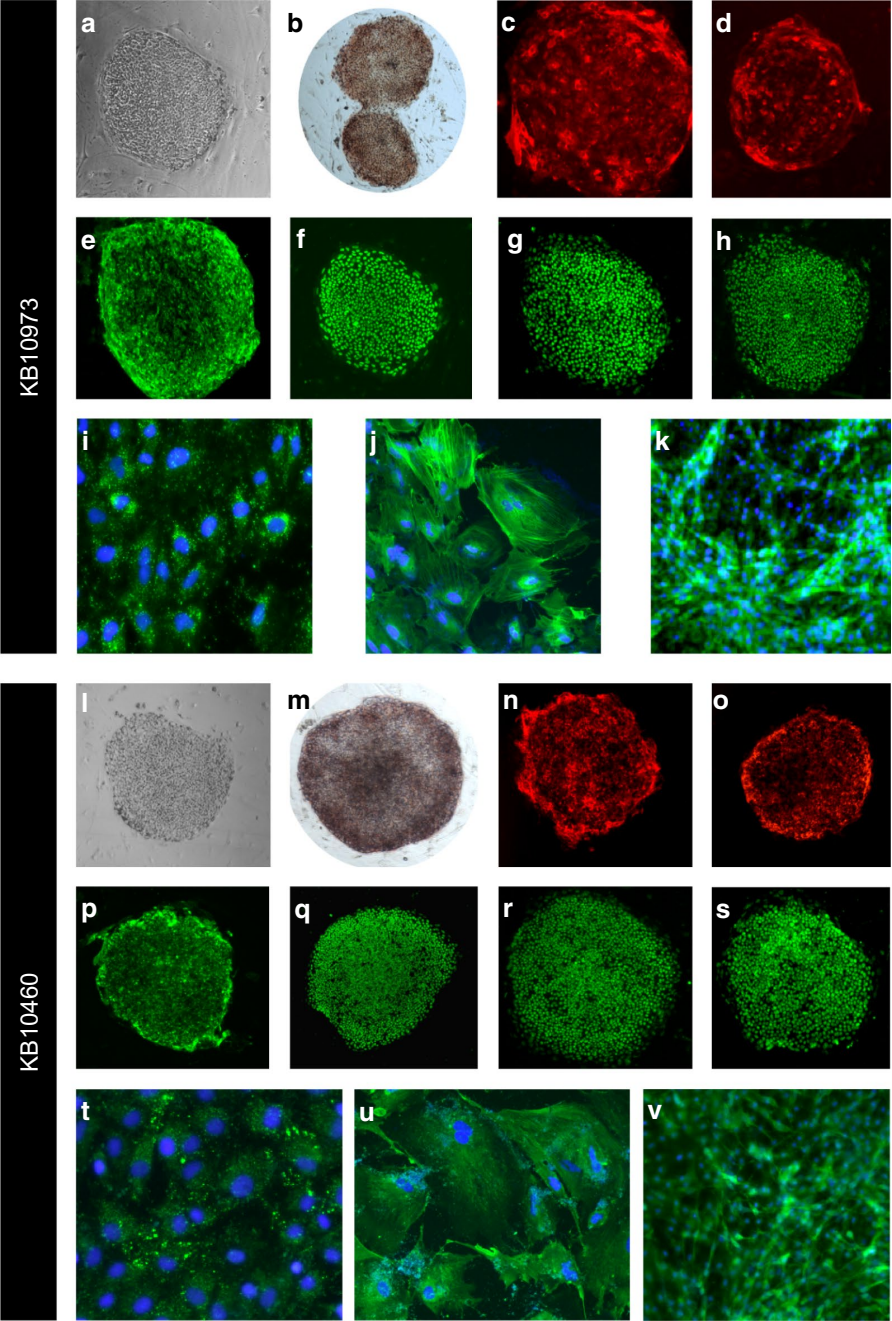
Images of orangutan iPSCs and derivatives. Data pertaining to KB10973 iPSC colony 1 (*Panels* **a**–**k**) and KB10460 iPSC colony 1 (*panels* **l**–**v**) are provided, as described in “Methods” Section. Light microscope images of iPSCs are shown in *panels* (**a**, **l**). Alkaline phosphatase staining is depicted in *panels* (**b**, **m**). Images of iPSCs immunostained for TRA-1-60 (*panels* **c**, **n**), TRA-1-81 (*panels* **d**, **o**), SSEA4 (*panels* **e**, **p**), OCT4 (*panels* **f**, **q**), SOX2 (*panels* **g**, **r**), and NANOG (*panels* **h**, **s**) are shown. DAPI nuclear counterstaining is not shown for the purpose of image clarity. *Panels* **t**–**v** provide the results of in vitro differentiation assays conducted on KB10973 iPSC colony 1 (*panels* **i**–**k**) and KB10460 iPSC colony 1. Cell populations derived from each of the three germ layers were detected by immunostaining for AFP (endoderm; *panels* **i**, **t**), SMA (mesoderm; *panels* **j**, **u**), and beta-III-tubulin (ectoderm; *panels* **k**, **v**). Nuclei were counterstained with DAPI (*blue*) in *panels* **t**–**v**

Primary skin fibroblast cultures previously derived from punch biopsies of the upper limbs of two Sumatran orangutans (KB10973, 29 year old male; KB10460, 43 year old female) [33] were obtained from the Zoological Society of San Diego. They were transduced with retroviruses designed to express the human *OCT4*, *SOX2*, *KLF4* and *c*-*MYC* genes, as previously described [37, 38] (“Methods” Section). We observed iPSC-like colonies by 2 weeks and clonally expanded TRA-1–60 positive colonies by 3 weeks, consistent with prior reports of reprogramming skin fibroblasts from healthy human donors [37, 39]. Candidate iPSC colonies KB10973-1 and KB10460-1 showed the expected morphological features and expressed protein biomarkers of pluripotency (Fig. 1). No differences were observed in orangutan iPSC with respect the colony morphology, passage characteristics, or molecular characteristics relative to other human iPSCs that were produced in our laboratory at approximately the same time [37, 38].

### In vitro differentiation of orangutan iPSCs and teratoma assays

**Fig. 2:**
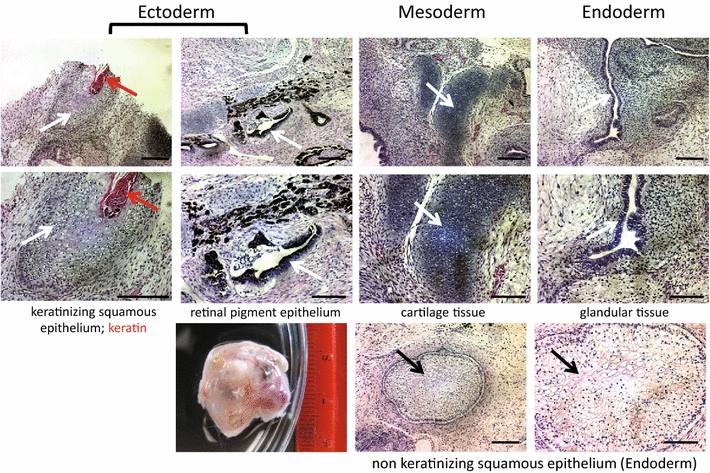
iPSC teratoma assay. Histological analysis of a teratoma derived from KB10973 iPSC colony 1 is provided. Cell populations representative of all three germ layers are present as indicated. *Arrows* correspond to annotations provided under each panel. *Scale bars* on the top two rows and bottom row of tissue sections are 200 µm and 50 µM, respectively

Orangutan iPSCs were capable of forming embryoid bodies which were capable of producing cells derived from all three germ layers, as assessed by in vitro differentiation assays (“Methods” Section) (Fig. 1). Furthermore, both orangutan iPSCs tested formed teratomas when injected into immune-deficient mice (“Methods” Section). Figure 2 depicts the histology of one of these teratomas, which demonstrated the presence of tissues representative of all three germ layers. The results of in vitro and in vivo differentiation assays conducted on orangutan iPSCs were indistinguishable from those of human iPSCs produced in our laboratory at approximately the same time [37, 38].

### Gene expression profiles of orangutan donor cells

**Fig. 3:**
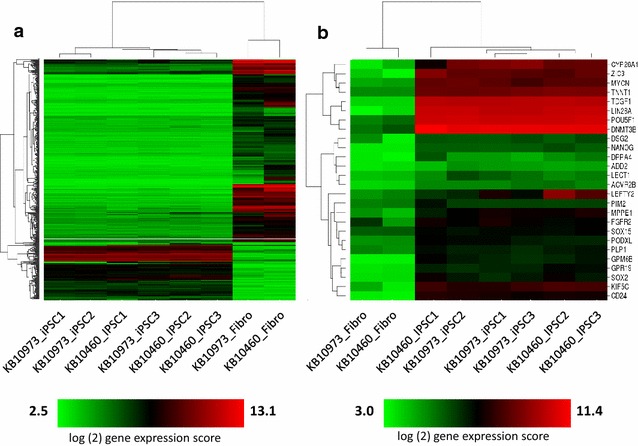
Gene expression profiles of orangutan fibroblasts and candidate iPSCs. Dendrograms depicting unsupervised hierarchical clustering analysis of gene expression data from skin fibroblasts and iPSCs derived from orangutan donors. Donor ID is provided along with colony number in the case of iPSCs. All analyses were conducted using average linkage and Euclidean distance. **a** Clustering based on log2-transformed gene expression scores from 546 probe sets with coefficient of variation (CV) greater than 0.25 and conducted using average linkage and Euclidean distance. **b** Clustering based on log2-transformed gene expression scores corresponding to 26 pluripotency-related genes that are differentially expressed between fibroblasts and iPSC (>1.5 fold change, FDR < 0.05). *Color bars* represent log-2 transformed gene expression values for each panel

We validated the robust expression of previously reported iPSC signature genes [39] in multiple candidate orangutan donor-derived iPSC colonies and skin fibroblasts based on global gene expression profiling data of over 18,000 transcripts conducted on human GeneChip microarrays (Additional file 1). Unsupervised hierarchical clustering analysis based on the expression of the most variable transcripts [i.e. coefficient of variation (CV) >0.25 across all samples)] (Fig. 3a) or preselected pluripotency biomarkers produced two distinct clusters consisting of skin fibroblasts and the iPSCs (Fig. 3b). As expected, the pluripotency biomarkers consistency showed higher expression in the iPSCs relative to the fibroblasts (Fig. 3b). All iPSC gene expression profiles showed similar expression signatures as demonstrated by the dendrograms shown in Fig. 3a, b. Since all samples were derived from orangutans, our report of differentially expressed genes between fibroblasts and iPSCs are valid. Nevertheless, any genes with low expression in fibroblasts and iPSCs samples could be influenced by mismatches between the orangutan transcript and human probes [40]. We provide alignments of human probe sequences corresponding to these pluripotency genes and orangutan genomic sequences in Additional file 2. Overall, the orangutan gene profiling results were consistent with that of human iPSCs produced in our laboratory at approximately the same time [37, 38].

### Genotypes and DNA copy number profiles of iPSCs

As expected, SNP genotypes in iPSCs and corresponding donor fibroblasts were concordant by analysis on CytoSNP-12 BeadArrays, consisting of 50 mer oligonucleotide probes designed to interrogate human genomes (“Methods” Section). Based on comparing hybridization signals from fibroblast and iPSC genomic DNA (gDNA) samples obtained from the same donor, we did not detect large copy number changes (CNCs) in the iPSCs analyzed (Additional file 3). Since all comparisons involve orangutan gDNAs that share approximately 0.974 nucleotide identity in unique gap free sequences with the human gDNAs [1], we minimize the chance that mismatches between orangutan gDNAs and the BeadArray probes will result in the spurious calling of genomic deletions in the orangutan iPSCs.

## Conclusions

We have demonstrated that cryopreserved skin fibroblasts from orangutans can be reprogrammed into iPSCs using the Yamanaka factors. Their ability to produce cells from all three germ layers in vitro and in vivo provides evidence of their pluripotency (Figs. 1, 2). Since iPSC colonies could be derived from these fibroblast cultures using the traditional retroviral methods, they should also have utility in evaluating non-retroviral reprogramming methods that do not alter the genome [41].

Orangutan iPSCs have potential applications for conservation biology in the present time as well as in the future. In terms of immediate relevance, orangutan iPSCs can be used to evaluate protocols for deriving germ cells from iPSCs [42–46]. Even if functional male and female gametes could be derived from genetically unaltered iPSCs in the future, numerous challenges for their applications in assisted reproductive technologies will remain [11, 12]. Furthermore, these technologies do not address vital issues regarding the preservation of the natural habitats of endangered species.

Transcriptome analyses of cultured human and great ape fibroblasts [47] and lymphoblastoid cell lines [48–50] have been used generate specific hypotheses regarding evolutionary selection in various primate lineages. In the former case [47], follow up studies were conducted on blood specimens that can be ethically obtained from living great apes and other non-human primates [33, 51, 52]. Although landmark studies have been performed on autopsy materials from great apes [53–57], appropriate biological specimens from deceased individuals are often difficult to obtain from a significant number of individuals. Likewise, ethical and sample acquisition issues strongly affect research into adaptations that affect developmental processes in each of these lineages.

Cross-species comparisons of the development and functions of central nervous system (CNS) cell lineages are of among those of greatest interest for human evolutionary biology [58]. iPSCs from humans, orangutans, and other great apes provide a gateway to address focused hypotheses regarding the timing of neurological adaptations that have occurred within and among these species [58]. A recent application of iPSC and genomic technologies to investigate enhancer divergence and cis-regulatory evolution in the human and chimp-derived cranial neural crest cells provides a model for future investigations in CNS cell types from orangutans, humans, and other great apes [35]. The mapping of fixed genetic changes across species that affect gene regulatory networks in a cell or tissue-specific manner would have enormous value for deciphering the molecular basis for phenotypic specializations in different ape lineages. As noted, iPSCs can also provide in vitro models of species-specific developmental processes that are not otherwise accessible due to numerous ethical and practical reasons [35]. Furthermore, more complex organoid models, such as ‘mini-brains’, that can be developed from iPSCs [36] would open new opportunities to engage in comparative neuroscience studies.

While purified cell types are useful for investigating cell autonomous mechanisms, recent technological innovations have allowed three-dimensional organoid models of the CNS [59] and other systems [60] to be derived from iPSCs in order to investigate non-cell autonomous mechanisms of biomedical relevance. These approaches could also be used to address questions relevant to human and great ape evolution. Finally, we note possible applications of genome editing technologies to investigate the functional relevance of genomic regions under selection within and among these primate lineages by introducing specific genetic changes in human and great ape iPSC-derived cell types [61].

## Methods

### Cell culture

Previously established primary orangutan skin fibroblast cultures were obtained from the Zoological Society of San Diego (ZSSD) [33]. Cultured skin fibroblasts were maintained in fibroblast growth media containing Dulbecco’s modified eagle medium (DMEM) containing 10 % fetal bovine serum (FBS), 2 mM l-glutamine, 100 µM nonessential amino acids and 0.5 % penicillin and streptomycin (Invitrogen). 293T packaging cells were maintained in the same media except with 10 % FBS. iPSCs were generated and maintained in human iPSC (hiPSC) growth medium containing DMEM/F12 containing 20 % KOSR (vol/vol) (Invitrogen), 10 ng/ml bFGF, 1 mM l-glutamine, 100 µM nonessential amino acids, 100 µM β-mercaptoethanol, 50 U/mL penicillin and 50 mg/mL streptomycin.

### Cellular reprogramming

Cellular reprogramming was conducted using the Yamanaka factors as previously described [37]. Retroviral pMX vectors for human OCT-3/4, SOX2, KLF4 and c-MYC were obtained from Addgene (https://www.addgene.org/). Briefly, GP2-293 packaging cells (Clontech) were plated at 2 × 10^6^ cells per 100-mm dish and incubated overnight. The cells were transfected with 5 µg of pMX vectors and 5 µg of pVSV-G vectors in the presence of Lipofectamine Transfection Reagent (Invitrogen) and the media was replaced the next day. Virus containing supernatant was collected on days 2 and 3 post-transfection and filtered through a 0.4 micron pore cellulose acetate filter (Corning).

Prior to transduction, primary orangutan cultures were plated at 8 × 10^5^ cells per well of a 6-well plate and incubated overnight. The cells were transduced with equal amounts of the four retroviruses in the presence of 5 ng/ml protamine sulfate. The following day the virus containing media was replaced with Fibroblast Growth (FG) medium. After another round of transduction, cells were trypsinized on day 6 and plated on a murine embryonic fibroblast (MEF) feeder layer at 2 × 10^5^ cells per well of a 6-well plate. The next day, the medium was replaced with hiPSC growth medium containing 1 mM valproic acid and 10 ng/mL βFGF. After daily media changing for 1 week, hiPSC culture medium conditioned with mitomycin C (Sigma-Aldrich) inactivated mouse embryonic fibroblasts (iMEFs) was used to support cell growth. Approximately 21 days after transduction, colonies exhibiting iPSC characteristics were picked mechanically and plated on iMEFs for expansion.

### Immunostaining

Cells were first fixed with 4 % paraformaldehyde, permeabilized with 1 % triton, and incubated with the primary antibody overnight at 4 °C. After washing three times with PBS, the cells were incubated with the secondary antibodies for 1 h at room temperature. The following primary antibodies were used: TRA-1-81 (mouse monoclonal against human, 1:100 dilution, EMD Millipore, Catalog #MAB4381), TRA-1-60 (mouse monoclonal against human, 1:100 dilution, EMD Millipore, Catalog #MAB4360), OCT4 (goat polyclonal against human, 1:100 dilution, R&D Systems, Catalog #AF1759), NANOG (goat polyclonal against human, 1:50 dilution, R&D systems, Catalog #1997), SOX2 (goat polyclonal against mouse, rat and human, 1:100 dilution, Santa Cruz Biotechnology, Catalog #sc17320), SSEA4 (mouse monoclonal against human, 1:100, Developmental Studies Hybridoma Bank, Catalog #MC-813-70), α-fetoprotein (AFP) (mouse monoclonal against human, 1:250 dilution, Sigma, Catalog #A8452), βIII-Tubulin (rabbit monoclonal against rat, 1:100 dilution, Covance Catalog #MRB-435P) and alpha smooth muscle actin (αSMA) (1:500 dilution, Abcam, Catalog #ab5694). Secondary antibodies used were rhodamine-labeled donkey anti-mouse IgG (1:100 dilution, Santa Cruz Biotechnology, Catalog #sc-2300), FITC-labeled donkey anti-rabbit IgG (1:100 dilution, Jackson ImmunoResearch, Catalog #711-095-152), FITC-labeled donkey anti-goat IgG (1:100 dilution, Jackson ImmunoResearch, Catalog #705-095-003) and Alexa Fluor 488 donkey anti-mouse IgG (1:500 dilution, ThermoFisher Scientific, Catalog #R37114). The Alkaline Phosphatase Detection Kit (Sigma-Aldrich) was used according to the manufacturer’s protocol.

### Genotyping and copy number analysis

Human CytoSNP-12 Infinium HD BeadChips (Illumina) that interrogate the genotypes of approximately 300,000 human single nucleotide polymorphisms (SNPs) were used to evaluate copy number in total genomic DNA from orangutan fibroblasts and iPSCs. Data filtering was performed using GenomeStudio (Illumina). Copy number analysis was performed using CNVPartition version 2.4.4, as previously described [62].

### Gene expression profiling

Biotin-labeled cRNA targets obtained from total RNA samples (Affymetrix GeneChip IVT Labeling Kit) were processed and analyzed on Affymetrix Human Genome 133A 2.0 GeneChips, as previously described [47]. All data normalization and analysis of differentially expressed genes (DEGSs) were conducted using WebArray software [63, 64]. We applied the RMA algorithm to generate log2-transformed gene expression values and used linear model statistical analysis (limma) to identify DEGSs with false discovery rates (FDRs), based on the spacings LOESS histogram (SPLOSH) method (Additional file 1B). We used CIMminer software (http://discover.nci.nih.gov/cimminer/home.do) for hierarchical clustering analysis [65].

### In vitro differentiation and teratoma assays

iPSCs were detached from culture dishes with collagenase IV, maintained in suspension to induce embryoid body formation, and subjected to an in vitro differentiation procedure as described [37]. For teratoma analysis, iPSCs from a confluent 10 cm^2^ plate were harvested and subcutaneously injected to the dorsal flanks of immunodeficient (SCID) mice (Jackson Laboratory). Teratomas were fixed in 10 % formalin, sectioned, stained with hematoxylin and eosin, and subject to histological analysis as described [37]. All mice used in this study were maintained in accordance with the Guide for the Care and Use of Animals (United States Department of Health and Human Services, Public Health Service, Bethesda, MD, 2012).

### Availability of supporting data

Gene expression scores and .cel files supporting the results of this article are available in the National Center for Biotechnology Information (NCBI) Gene Expression Omnibus (GEO) repository [Series Accession Number GSE69603 and http://www.ncbi.nlm.nih.gov/geo/query/acc.cgi?acc=GSE69603].

## Additional files

10.1186/s13104-015-1567-0 Log-transformed gene expression scores from orangutan fibroblasts and iPSCs. A complete list of log-transformed gene expression scores from orangutan fibroblasts and iPSCs is provided along with their percentile rank within a given samples. Differentially expressed genes (1.5-fold change FDR<0.05) are also provided.
10.1186/s13104-015-1567-0 Orangutan sequences and probe tilings present in the human GeneChip microarrays that pertain to pluripotency genes. Alignments of orangutan sequences and selected probe tilings from the human GeneChip arrays pertaining to pluripotency genes listed in reference [39] are provided.
10.1186/s13104-015-1567-0 Copy number variation analysis for orangutan iPSCs. Log base 2 of the ratio of subject (iPSC) and reference (fibroblast) R values for probes in the CNV BeadArray are provided for KB10973 iPSC colony 1 and KB10460 iPSC colony 1. All data is mapped onto the human karyotype.

## Abbreviations

iPSC: induced pluripotent stem cell
FBS: fetal bovine serum
CNC: copy number change
