# Supplementary material for: Derivation of induced pluripotent stem cells from orangutan skin fibroblasts
Source: BMC Res Notes. 2015 Oct 16;8:577. doi: 10.1186/s13104-015-1567-0 (PMC4609060; doi:10.1186/s13104-015-1567-0)
Supplement: Supplementary file 2 — 10.1186/s13104-015-1567-0 Orangutan sequences and probe tilings present in the human GeneChip microarrays that pertain to pluripotency genes. Alignments of orangutan sequences and selected probe tilings from the human GeneChip arrays pertaining to pluripotency genes listed in reference [39] are provided. [file 13104_2015_1567_MOESM2_ESM.docx]

**Additional file 4. Orangutan sequences and probe tilings present in the human GeneChip microarrays that pertain to pluripotency genes.**

**>HG-U133_PLUS_2:219823_AT GENE LIN28A**

cacactactgtattttggatggatcaaacctccttaattttaatttctaatcctaaagtaaagagatgcaattgggggccttccatgtagaaagtggggtcaggaggccaagaaagggaatatgaatgtatatccaagtcactcaggaacttttatgcaggtgctagaaactttatgtcaaagtggccacaagattgtttaataggagacgaacgaatgtaactccatgtttactgctaaaaaccaaagctttgtgtaaaatcttgaatttatggggcgggagggtaggaaagcctgtacctgtctgtttttttcctgatccttttccctcattcctgaactgcaggagactgagcccctttgggctttggtgaccccatcactggggtgtgtttatttgatggttgattttgctgtactgggtacttcctttcccattttctaatcattttttaacacaagctgactcttccctt

**Alignment (orangutan on top; human on bottom)**

000000001 cacactactgtattttggatggatcaaacctccttaattttaatttctaa 000000050

<<<<<<<<< ||||||||||||||||||||||||||||||| |||||||||||||||||| <<<<<<<<<

203941672 cacactactgtattttggatggatcaaacctacttaattttaatttctaa 203941623

000000051 tcctaaagtaaagagatgcaattgggggccttccatgtagaaagtggggt 000000100

<<<<<<<<< ||||||||||||||||||||||||||||||||||||||| |||||||||| <<<<<<<<<

203941622 tcctaaagtaaagagatgcaattgggggccttccatgtaaaaagtggggt 203941573

000000101 caggaggccaagaaagggaatatgaatgtatatccaagtcactcaggaac 000000150

<<<<<<<<< |||||||||||||||||||||||||||||||||||||||||||||||||| <<<<<<<<<

203941572 caggaggccaagaaagggaatatgaatgtatatccaagtcactcaggaac 203941523

000000151 ttttatgcaggtgctagaaactttatgtcaaagtggccacaagattgttt 000000200

<<<<<<<<< ||||||||||||||||||||| |||||||||||||||||||||||||||| <<<<<<<<<

203941522 ttttatgcaggtgctagaaac.ttatgtcaaagtggccacaagattgttt 203941474

000000201 aataggagacgaacgaatgtaactccatgtttactgctaaaaaccaaagc 000000250

<<<<<<<<< ||||||||||||||||||||||||||||||||||||||| |||||||||| <<<<<<<<<

203941473 aataggagacgaacgaatgtaactccatgtttactgctagaaaccaaagc 203941424

000000251 tttgtgtaaaatcttgaatttatggggcgggagggtaggaaagcctgtac 000000300

<<<<<<<<< ||||||||||||||||||||||||||| |||||||||||||||||||||| <<<<<<<<<

203941423 tttgtgtaaaatcttgaatttatggggagggagggtaggaaagcctgtac 203941374

000000301 ctgtctgtttttttcctgatccttttccctcattcctgaactgcaggaga 000000350

<<<<<<<<< |||||||||||| ||||||||||||||||||||||||||||||||||||| <<<<<<<<<

203941373 ctgtctgtttttctcctgatccttttccctcattcctgaactgcaggaga 203941324

000000351 ctgagcccctttgggctttggtgaccccatcactggggtgtgtttatttg 000000400

<<<<<<<<< ||||||||| |||||||||||||||||||||||||||||||||||||||| <<<<<<<<<

203941323 ctgagccccattgggctttggtgaccccatcactggggtgtgtttatttg 203941274

000000401 atggttgattttgctgtactgggtacttcctttcccattttctaatcatt 000000450

<<<<<<<<< |||||||||||||||||||||||||||||||||||||||||||||||||| <<<<<<<<<

203941273 atggttgattttgctgtactgggtacttcctttcccattttctaatcatt 203941224

000000451 ttttaacacaagctgactcttccctt 000000476

<<<<<<<<< |||||||||| ||||||||||||||| <<<<<<<<<

203941223 ttttaacacatgctgactcttccctt 203941198

| Probe Sequence(5'-3') | Probe X | Probe Y | Probe Interrogation Position | Target Strandedness |
| --- | --- | --- | --- | --- |
| CACACTACTGTATTTTGGATGGATC | 687 | 307 | 2941 | Antisense |
| GATGGATCAAACCTCCTTAATTTTA | 1014 | 665 | 2958 | Antisense |
| AGATGCAATTGGGGGCCTTCCATGT | 1157 | 107 | 3004 | Antisense |
| GTATATCCAAGTCACTCAGGAACTT | 1019 | 721 | 3068 | Antisense |
| TCAGGAACTTTTATGCAGGTGCTAG | 829 | 1019 | 3083 | Antisense |
| GCAGGTGCTAGAAACTTTATGTCAA | 748 | 527 | 3097 | Antisense |
| GAATGTAACTCCATGTTTACTGCTA | 947 | 557 | 3155 | Antisense |
| GGTGACCCCATCACTGGGGTGTGTT | 614 | 817 | 3310 | Antisense |
| GATGGTTGATTTTGCTGTACTGGGT | 1123 | 665 | 3340 | Antisense |
| TTGCTGTACTGGGTACTTCCTTTCC | 1014 | 1147 | 3351 | Antisense |
| TTTAACACAAGCTGACTCTTCCCTT | 570 | 1103 | 3392 | Antisense |

**>HG-U133_PLUS_2:206286_S_AT GENE TDGF1**

gtggaccttagaatacagttttgagtagagttgatcaaaatcaattaaaatagtctctttaaaaggaaagaaaacatctttaaggggaggaaccagagtgctgaaggaatggaagtccatctgcgtgtgtgcagggagactgggtaggaaagaggaagcaaatagaagagagaggttgaaaaacaaaatgggttacttgattggtgattaggtggtggtagagaagcaagtaaaaaggctaaatggaagggcaagtttccatcatctatagaaagctatataagacaagaactcccctttttttcccaaaggcattataaaaagaatgaagcctccttagaaaaaaaattatacctcaatgtccccaacaagattgcttaataaattgtgtttcctccaagctattcaattcttttaactgttgtagaagacaaaatgttcacaatatatttagttgtaaaccaagtgatcaaactacatattgtaaagcccattttt

**Alignment (orangutan on top; human on bottom)**

000000001 gtggaccttagaa.tacagttt.tgagtagagttgatcaaaatcaa.tta 000000047

<<<<<<<<< ||||||||||||| |||||||| ||||||||||||||||||||||| ||| <<<<<<<<<

100019721 gtggaccttagaaatacagtttatgagtagagttgatcaaaatcaattta 100019672

000000048 aaatagtctctttaaaaggaaag.aaaacatctttaaggggaggaaccag 000000096

<<<<<<<<< ||||||||||||||||||||||| |||||||||||||||||||||||||| <<<<<<<<<

100019671 aaatagtctctttaaaaggaaagaaaaacatctttaaggggaggaaccag 100019622

000000097 agtgctgaa.ggaat.ggaagtccatctgcgtgtgtgcagggagactggg 000000144

<<<<<<<<< ||||||||| ||||| ||||||||||||| |||||||||||||||||||| <<<<<<<<<

100019621 agtgctgaaaggaatgggaagtccatctgtgtgtgtgcagggagactggg 100019572

000000145 taggaaagaggaagcaaatagaagagagaggttg.aaaaac.aaaatggg 000000192

<<<<<<<<< |||||||||||||||||||||||||||||||||| |||||| |||||||| <<<<<<<<<

100019571 taggaaagaggaagcaaatagaagagagaggttggaaaaacaaaaatggg 100019522

000000193 ttacttgattggtgattaggtggtggtagagaagcaagtaaaaaggctaa 000000242

<<<<<<<<< |||||||||||||||||||||||||||||||||||||||||||||||||| <<<<<<<<<

100019521 ttacttgattggtgattaggtggtggtagagaagcaagtaaaaaggctaa 100019472

000000243 atggaagggcaagtttccatcatctatagaaagctatataagacaagaac 000000292

<<<<<<<<< ||||||||||||||||||||||||||||||||||||||||||||||| || <<<<<<<<<

100019471 atggaagggcaagtttccatcatctatagaaagctatataagacaaggac 100019422

000000293 tcccctttttttcccaaaggcattataaaaagaatgaagcctccttagaa 000000342

<<<<<<<<< |||||||||||||||||||||||| |||||||||||||| |||||||||| <<<<<<<<<

100019421 tcccctttttttcccaaaggcattgtaaaaagaatgaagtctccttagaa 100019372

000000343 aaaaaattatacctcaatgtccccaacaagattgcttaataaattgtgtt 000000392

<<<<<<<<< |||||||||||||||||||||||||||||||||||||||||||| |||| <<<<<<<<<

100019371 aaaaaattatacctcaatgtccccaacaagattgcttaataaat..tgtt 100019324

000000393 tcctccaagctattcaattcttttaactgttgtagaagacaaaatgttca 000000442

<<<<<<<<< ||||||||||||| ||||| ||||||||||||||||||| |||||||||| <<<<<<<<<

100019323 tcctccaagctatgcaattgttttaactgttgtagaagagaaaatgttca 100019274

000000443 caatatatttagttgtaaaccaagtgatcaaactacatattgtaaagccc 000000492

<<<<<<<<< |||||||||||||||||||||||||||||||||||||||||||||||||| <<<<<<<<<

100019273 caatatatttagttgtaaaccaagtgatcaaactacatattgtaaagccc 100019224

000000493 attttt 000000498

<<<<<<<<< |||||| <<<<<<<<<

100019223 attttt 100019218

| Probe Sequence(5'-3') | Probe X | Probe Y | Probe Interrogation Position | Target Strandedness |
| --- | --- | --- | --- | --- |
| GTGGACCTTAGAATACAGTTTTGAG | 809 | 787 | 1481 | Antisense |
| TAAGGGGAGGAACCAGAGTGCTGAA | 149 | 1037 | 1561 | Antisense |
| GGAAGTCCATCTGCGTGTGTGCAGG | 341 | 867 | 1591 | Antisense |
| GTGTGCAGGGAGACTGGGTAGGAAA | 369 | 777 | 1607 | Antisense |
| GGGTTACTTGATTGGTGATTAGGTG | 161 | 807 | 1670 | Antisense |
| AAGGGCAAGTTTCCATCATCTATAG | 1065 | 263 | 1727 | Antisense |
| TATACCTCAATGTCCCCAACAAGAT | 544 | 1083 | 1830 | Antisense |
| AATGTCCCCAACAAGATTGCTTAAT | 710 | 271 | 1838 | Antisense |
| TGTGTTTCCTCCAAGCTATTCAATT | 98 | 919 | 1867 | Antisense |
| GCTATTCAATTCTTTTAACTGTTGT | 497 | 513 | 1881 | Antisense |
| ACTACATATTGTAAAGCCCATTTTT | 1059 | 173 | 1954 | Antisense |

**>HG-U133_PLUS_2:220668_s_at GENE DNMT3B**

ggcacaggtccccagatgagaagtctgctaccctcatttctcatctttttactaaactcagaggcagtgacagcagtcagggacagacatacatttctcataccttccccacatctgagagatgacagggaaaactgcaaagctcggtgctccctttggagattttttaatccttttttattccataagaagtcgtttttagggagaacgggaattcagacaagctgcatttcagaaatgctgtcataatggtttttaacaccttttactcttcttactggtgctattttgtagaataaggaacaacgttgacaagttttgtggggctttttatacactttttaaaatctcaaacttctatttttatgtttaacgttttcattaaaatttttttgtaactggagccacgacgtaacaaatatggggaaaaaactgtgccttgtttcaacagtttttgctaatttttaggctgaaagatgacggatgcctagagtttaccttat

**Alignment (orangutan on top; human on bottom)**

00000001 ggcacaggtccccagatgagaagtctgctaccctcatttctcatcttttt 00000050

>>>>>>>> |||||||||||||||||||||||||||||||||||||||||||||||||| >>>>>>>>

30015756 ggcacaggtccccagatgagaagtctgctaccctcatttctcatcttttt 30015805

00000051 actaaactcagaggcagtgacagcagtcagggacagacatacatttctca 00000100

>>>>>>>> |||||||||||||||||||||||||||||||||||||| |||| |||||| >>>>>>>>

30015806 actaaactcagaggcagtgacagcagtcagggacagacgtacacttctca 30015855

00000101 taccttccccacatctgagagatgacagggaaaactgcaaagctcggtgc 00000150

>>>>>>>> |||||||||| ||||||||||||||||||||||||||||||||||||||| >>>>>>>>

30015856 taccttcccctcatctgagagatgacagggaaaactgcaaagctcggtgc 30015905

00000151 tccctttggagattttttaatccttttttattccataagaagtcgttttt 00000200

>>>>>>>> ||| ||||||||||||| |||||||||||||||| | ||||||||||||| >>>>>>>>

30015906 tcc.tttggagatttttaaatccttttttattccgtgagaagtcgttttt 30015954

00000201 agggagaacgggaattcagacaagctgcatttcagaaatgctgtcataat 00000250

>>>>>>>> |||||||| |||| |||||||||||||||||||||||||||||||||||| >>>>>>>>

30015955 agggagaaggggagttcagacaagctgcatttcagaaatgctgtcataat 30016004

00000251 ggtttttaacaccttttactcttcttactggtgctattttgtagaataag 00000300

>>>>>>>> |||||||||||||||||||||||||||||||||||||||||||||||||| >>>>>>>>

30016005 ggtttttaacaccttttactcttcttactggtgctattttgtagaataag 30016054

00000301 gaacaacgttgacaagttttgtggggctttttatacacttttt..aaaat 00000348

>>>>>>>> ||||||||||||| ||||||||||||||||||||||||||||| ||||| >>>>>>>>

30016055 gaacaacgttgaccagttttgtggggctttttatacactttttttaaaat 30016104

00000349 ctcaaacttctatttttatgtttaacgttttcattaaaatttttttgtaa 00000398

>>>>>>>> |||||||||||||||||||||||||||||||||||||||||||||||||| >>>>>>>>

30016105 ctcaaacttctatttttatgtttaacgttttcattaaaatttttttgtaa 30016154

00000399 ctggagccacgacgtaacaaatatggggaaaaaactgtgccttgtttcaa 00000448

>>>>>>>> |||||||||||||||||||||||||||||||||||||||||||||||||| >>>>>>>>

30016155 ctggagccacgacgtaacaaatatggggaaaaaactgtgccttgtttcaa 30016204

00000449 cagtttttgctaatttttaggctgaaagatgacggatgcctagagtttac 00000498

>>>>>>>> |||||||||||||||||||||||||||||||| |||||||||||||| >>>>>>>>

30016205 cagtttttgctaatttttaggctgaaagatga....tgcctagagtttac 30016250

00000499 cttat 00000503

>>>>>>>> ||||| >>>>>>>>

30016251 cttat 30016255

| Probe Sequence(5'-3') | Probe X | Probe Y | Probe Interrogation Position | Target Strandedness |
| --- | --- | --- | --- | --- |
| GGCACAGGTCCCCAGATGAGAAGTC | 71 | 873 | 3612 | Antisense |
| GATGAGAAGTCTGCTACCCTCATTT | 836 | 671 | 3626 | Antisense |
| TTCTCATCTTTTTACTAAACTCAGA | 837 | 1135 | 3649 | Antisense |
| GTGACAGCAGTCAGGGACAGACATA | 987 | 769 | 3678 | Antisense |
| CAGACATACATTTCTCATACCTTCC | 259 | 329 | 3695 | Antisense |
| GAAAACTGCAAAGCTCGGTGCTCCC | 559 | 585 | 3741 | Antisense |
| CTCGGTGCTCCCTTTGGAGATTTTT | 531 | 383 | 3754 | Antisense |
| CTTTTACTCTTCTTACTGGTGCTAT | 447 | 365 | 3874 | Antisense |
| GTAACTGGAGCCACGACGTAACAAA | 757 | 735 | 4006 | Antisense |
| GAAAAAACTGTGCCTTGTTTCAACA | 212 | 585 | 4037 | Antisense |
| GACGGATGCCTAGAGTTTACCTTAT | 513 | 603 | 4090 | Antisense |

**>HG-U133_PLUS_2:208286_X_AT GENE POU5F1**

aacccacactgcagcagatcagccacatcgcccagcagcttgggctcgagaaggatgtggtccgagtgtggttctgtaaccggcgccagaagggcaagcgatcaagcagcgactatgcacaacgagaggattttgaggctgctgggtctcctttctcagggggaccagtgtcctttcctctggccccagggccccattttggtgccccaggctatgggagccctcacttcactgcactgtactcctcggtccctttccctgagggggaagcctttccccctgtctctgtcaccactctgggctctcccttgcattcaaactgaggtgcctgcctgcccttctaggaatgggggacagggggaggggaggagctagggaaagaaaacctggagtttgtgccagggtttttggattaagttcttcattcactaaggaaggaattgggaacacaaagggtgggggcaggggagtttggggcaactggttggagggaaggtgaagttcaatgatgctcttgattttaatcccacatcatgtatcacttttttct

**Alignment (orangutan on top; human on bottom)**

000001 aacccacactgcagcagatcagccacatcgcccagcagcttgggctcgag 000050

<<<<<< |||||||||||||||||||||||||||| ||||||||||||||||||||| <<<<<<

571606 aacccacactgcagcagatcagccacattgcccagcagcttgggctcgag 571557

000051 aaggat 000056

<<<<<< |||||| <<<<<<

571556 aaggat 571551

000057 gtggtccgagtgtggttctgtaaccggcgccagaagggcaagcgatcaag 000106

<<<<<< |||||||||||||||||||||||||||||||||||||||||||||||||| <<<<<<

571368 gtggtccgagtgtggttctgtaaccggcgccagaagggcaagcgatcaag 571319

000107 cagcgactatgcacaacgagaggattttgaggctgctgggtctcctttct 000156

<<<<<< ||| |||||||||||||||||||||||||||||||||||||||||||||| <<<<<<

571318 cagtgactatgcacaacgagaggattttgaggctgctgggtctcctttct 571269

000157 cagggggaccagtgtcctttcctctggccccagggccccattttggtgcc 000206

<<<<<< ||||||||||||||||||||||||||||||||||||||||||||||| || <<<<<<

571268 cagggggaccagtgtcctttcctctggccccagggccccattttggtacc 571219

000207 ccaggctatgggagccctcacttcactgcactgtactcctcggtcccttt 000256

<<<<<< |||||||||||||||||||||||||||||||||||||||||||||||||| <<<<<<

571218 ccaggctatgggagccctcacttcactgcactgtactcctcggtcccttt 571169

000257 ccctgagggggaagcctttccccctgtctctgtcaccactctgggctctc 000306

<<<<<< |||||||||||||||||||||||||||||||||||||||||||||||||| <<<<<<

571168 ccctgagggggaagcctttccccctgtctctgtcaccactctgggctctc 571119

000307 ccttgcattcaaactgaggtgcctgcctgcccttctaggaatgggggaca 000356

<<<<<< || |||||||||||||||| ||||||||||||||||||||||||||| <<<<<<

571118 ccatgcattcaaactgagg....tgcctgcccttctaggaatgggggaca 571073

000357 gggggaggggaggagctagggaaagaaaacctggagtttgtgccaggg.t 000405

<<<<<< |||||||||||||||||||||||||| ||||||||||||||||||||| | <<<<<<

571072 gggggaggggaggagctagggaaagagaacctggagtttgtgccagggct 571023

000406 ttttggattaagttcttcattcactaaggaaggaattgggaacacaaagg 000455

<<<<<< ||| |||||||||||||||||||||||||||||||||||||||||||||| <<<<<<

571022 tttgggattaagttcttcattcactaaggaaggaattgggaacacaaagg 570973

000456 gtgggggcaggggagtttggggcaactggttggagggaaggtgaagttca 000505

<<<<<< ||||||||| ||||||||||||||||||||||||||||||||||| |||| <<<<<<

570972 gtgggggcaagggagtttggggcaactggttggagggaaggtgaaattca 570923

000506 atgatgctcttgattttaatcccacatcatgtatcacttttttct 000550

<<<<<< |||| |||||||||||||||||||||||||||||||||||||||| <<<<<<

570922 atgacgctcttgattttaatcccacatcatgtatcacttttttct 570878

| Probe Sequence(5'-3') | Probe X | Probe Y | Probe Interrogation Position | Target Strandedness |
| --- | --- | --- | --- | --- |
| AACCCACACTGCAGCAGATCAGCCA | 553 | 241 | 589 | Antisense |
| ATCGCCCAGCAGCTTGGGCTCGAGA | 935 | 47 | 615 | Antisense |
| GATGTGGTCCGAGTGTGGTTCTGTA | 1130 | 667 | 642 | Antisense |
| GTAACCGGCGCCAGAAGGGCAAGCG | 960 | 737 | 664 | Antisense |
| GGGCAAGCGATCAAGCAGCGACTAT | 257 | 797 | 680 | Antisense |
| AGGATTTTGAGGCTGCTGGGTCTCC | 497 | 83 | 715 | Antisense |
| TCTCAGGGGGACCAGTGTCCTTTCC | 520 | 1003 | 742 | Antisense |
| TTTGGTGCCCCAGGCTATGGGAGCC | 781 | 1091 | 786 | Antisense |
| GCTCTCCCTTGCATTCAAACTGAGG | 338 | 507 | 889 | Antisense |
| CTGCCTGCCCTTCTAGGAATGGGGG | 326 | 391 | 917 | Antisense |
| CCCACATCATGTATCACTTTTTTCT | 751 | 421 | 1114 | Antisense |

**>HG-U133_PLUS_2:207197_AT GENE ZIC3**

cttacagtgtcagtttccatctgggaagactctcctttctttatctctatctcagatggttgtttaactgcgagtttaaatgtgtttgtcctggattttcggcatgcaaatcaaatattactgatcaattcagttagtggccatgacatctcaatcttgtacttcaaagactgagaagctggatttaatcatccctgccctacatatataaacataaggtaacctactgaattttatgtcccttagttctttattaccttacataaaaatgaaaattgcggcaggatgcatgtctgtctgttctatctagagatcacccatatacctatatatgtttgtatctatgacttatctaatctgcctatcaatctatctagtagctatctatatattttcaaaagatagcttatgtctaaaacagtggtgatgagtaaggccagttgagcattgcttacttatggttaaagtgcttcttaaaagaaaccatagtccatttacaattttggaaggcaaaggctgatttgtttgctgtatatagttccaatccataattaccgcca

**Alignment (orangutan on top; human on bottom)**

000000001 cttacagtgtcagtttccatctgggaagactctcctttctttatctctat 000000050

>>>>>>>>> |||||||||||||||||||||||||||||||||||||||||||||||||| >>>>>>>>>

137151993 cttacagtgtcagtttccatctgggaagactctcctttctttatctctat 137152042

000000051 ctcagatggttgtttaactgcgagtttaaatgtgtttgtcctggattttc 000000100

>>>>>>>>> |||||||||||||||||||||||||||||||||||||||||||||||||| >>>>>>>>>

137152043 ctcagatggttgtttaactgcgagtttaaatgtgtttgtcctggattttc 137152092

000000101 ggcatgcaaatcaaatattactgatcaattcagttagtggccatgacatc 000000150

>>>>>>>>> |||||||||||||||||||||||||||||||||||||||||||||||||| >>>>>>>>>

137152093 ggcatgcaaatcaaatattactgatcaattcagttagtggccatgacatc 137152142

000000151 tcaatcttgtacttcaaagactgagaagctggatttaatcatccctgccc 000000200

>>>>>>>>> |||||||||||||||||||||||||||||||||||||||||||||||||| >>>>>>>>>

137152143 tcaatcttgtacttcaaagactgagaagctggatttaatcatccctgccc 137152192

000000201 tacatatataaacataaggtaacctactgaattttatgtcccttagttct 000000250

>>>>>>>>> |||||||||||||||||||||||||||||||||||||||||||||||||| >>>>>>>>>

137152193 tacatatataaacataaggtaacctactgaattttatgtcccttagttct 137152242

000000251 ttattaccttacataaaaatgaaaattgcggcaggatgcatgtctgtctg 000000300

>>>>>>>>> |||||||||||||||||||||||||||||||||||||||||||||||||| >>>>>>>>>

137152243 ttattaccttacataaaaatgaaaattgcggcaggatgcatgtctgtctg 137152292

000000301 ttctatctagagatcacccatatacctatatatgtttgtatctatgactt 000000350

>>>>>>>>> |||||||||||||||||||||||||||||||||||||||||||||||||| >>>>>>>>>

137152293 ttctatctagagatcacccatatacctatatatgtttgtatctatgactt 137152342

000000351 atctaatctgcctatcaatctatctagtagctatctatatattttcaaaa 000000400

>>>>>>>>> |||||||||||||||||||||||||||||||||||||||||||||||||| >>>>>>>>>

137152343 atctaatctgcctatcaatctatctagtagctatctatatattttcaaaa 137152392

000000401 gatagcttatgtctaaaacagtggtgatgagtaaggccagttgagcattg 000000450

>>>>>>>>> |||||||||||||| ||||||||||||||||||||||||||||||||||| >>>>>>>>>

137152393 gatagcttatgtctgaaacagtggtgatgagtaaggccagttgagcattg 137152442

000000451 cttacttatggttaaagtgcttcttaaaagaaaccatagtccatttacaa 000000500

>>>>>>>>> |||||||||||||||||||||||||||||||| ||||||||||||||||| >>>>>>>>>

137152443 cttacttatggttaaagtgcttcttaaaagaa.ccatagtccatttacaa 137152491

000000501 ttttggaaggcaaaggctgatttgtttgctgtatatagttccaatccata 000000550

>>>>>>>>> ||||||||||||||||||||||||||||||||||||||||| ||| |||| >>>>>>>>>

137152492 ttttggaaggcaaaggctgatttgtttgctgtatatagttc.aattcata 137152540

000000551 atta 000000554

>>>>>>>>> |||| >>>>>>>>>

137152541 atta 137152544

| Probe Sequence(5'-3') | Probe X | Probe Y | Probe Interrogation Position | Target Strandedness |
| --- | --- | --- | --- | --- |
| CTTACAGTGTCAGTTTCCATCTGGG | 906 | 363 | 2858 | Antisense |
| TCCATCTGGGAAGACTCTCCTTTCT | 387 | 977 | 2873 | Antisense |
| GTTTGTCCTGGATTTTCGGCATGCA | 517 | 719 | 2941 | Antisense |
| GTGGCCATGACATCTCAATCTTGTA | 552 | 791 | 2994 | Antisense |
| GAAGCTGGATTTAATCATCCCTGCC | 681 | 571 | 3032 | Antisense |
| TCATCCCTGCCCTACATATATAAAC | 843 | 1017 | 3046 | Antisense |
| TATGTCCCTTAGTTCTTTATTACCT | 763 | 1077 | 3092 | Antisense |
| GGATGCATGTCTGTCTGTTCTATCT | 1049 | 839 | 3141 | Antisense |
| GACTTATCTAATCTGCCTATCAATC | 656 | 609 | 3203 | Antisense |
| GAGTAAGGCCAGTTGAGCATTGCTT | 555 | 649 | 3286 | Antisense |
| TAGTTCCAATCCATAATTACCGCCA * | 372 | 1069 | 3393 | Antisense |

*End of probe sequence not present in side by side alignment.

**>HG-U133_PLUS_2:206424_AT GENE CYP26A1**

Tccaggagggtttcgggttgctctgaagacttttgaattaaatggataccagattcccaagggctggaatgttatctacagtatctgtgatactcatgatgtggcagagatcttcaccaacaaggaagaatttaatcctgaccgattcatgctgcctcacccagaggatgcatccaggttcagcttcattccatttggaggaggccttaggagctgtgtaggcaaagaatttgcaaaaattcttctcaaaatatttacagtggagctggccaggcattgtgactggcagcttctaaatggacctcctacaatgaaaaccagtcccaccgtgtatcctgtggacaatctccctgcaagattcacccatttccatggggaaatctgatgagcttgaatgttcaaacctgagacttattggaagtgtacatatgagtttttaaggagtgttgtgttgactttatatttaatttctaaatgtatattataatatttatgtgttttgactatactaccacaatctttaaat

**Alignment (orangutan on top; human on bottom)**

00000001 tccaggagggtttcgggttgctctgaagacttttgaattaaat 00000043

>>>>>>>> ||||||||||||||||||||||||||||||||||||||||||| >>>>>>>>

91813647 tccaggagggtttcgggttgctctgaagacttttgaattaaat 91813689

00000044 ggataccagattcccaagggctggaatgttatctacagtatctgtgatac 00000093

>>>>>>>> |||||||||||||||||||||||||||||||||||||||||||||||||| >>>>>>>>

91813956 ggataccagattcccaagggctggaatgttatctacagtatctgtgatac 91814005

00000094 tcatgatgtggcagagatcttcaccaacaaggaagaatttaatcctgacc 00000143

>>>>>>>> ||||||||||||||||||||||||||||||||||||||||||||||||| >>>>>>>>

91814006 tcatgatgtggcagagatcttcaccaacaaggaagaatttaatcctgaca 91814055

00000144 gattcatgctgcctcacccagaggatgcatccaggttcagcttcattcca 00000193

>>>>>>>> |||||||||||||||||||||||||||||||||||||||||||||||||| >>>>>>>>

91814056 gattcatgctgcctcacccagaggatgcatccaggttcagcttcattcca 91814105

00000194 tttggaggaggccttaggagctgtgtaggcaaagaatttgcaaaaattct 00000243

>>>>>>>> |||||||||||||||||||||||||||||||||||||||||||||||||| >>>>>>>>

91814106 tttggaggaggccttaggagctgtgtaggcaaagaatttgcaaaaattct 91814155

00000244 tctcaaaatatttacagtggagctggccaggcattgtgactggcagcttc 00000293

>>>>>>>> |||||||||||||||||||||||||||||||||||||||||||||||||| >>>>>>>>

91814156 tctcaaaatatttacagtggagctggccaggcattgtgactggcagcttc 91814205

00000294 taaatggacctcctacaatgaaaaccagtcccaccgtgtatcctgtggac 00000343

>>>>>>>> ||||||||||||||||||||||||||||||| |||||||||||||||||| >>>>>>>>

91814206 taaatggacctcctacaatgaaaaccagtcctaccgtgtatcctgtggac 91814255

00000344 aatctccctgcaagattcacccatttccatggggaaatctgatgagcttg 00000393

>>>>>>>> |||||||||||||||||||||||||||||||||||||||||||||||||| >>>>>>>>

91814256 aatctccctgcaagattcacccatttccatggggaaatctgatgagcttg 91814305

00000394 aatgttcaaacctgagacttattggaagtgtacatatgagtttttaagga 00000443

>>>>>>>> |||| ||||||||||||||||||||||||||||||||||||||||||||| >>>>>>>>

91814306 aatgctcaaacctgagacttattggaagtgtacatatgagtttttaagga 91814355

00000444 gtgttgtgttgactttatatttaatttctaaatgtatattataatattta 00000493

>>>>>>>> |||||||||||||||||||||||||||||||||||||||||||||||||| >>>>>>>>

91814356 gtgttgtgttgactttatatttaatttctaaatgtatattataatattta 91814405

00000494 tgtgttttgactatactaccacaatctttaaat 00000526

>>>>>>>> ||||||||||||||||||||||||||||||||| >>>>>>>>

91814406 tgtgttttgactatactaccacaatctttaaat 91814438

| Probe Sequence(5'-3') | Probe X | Probe Y | Probe Interrogation Position | Target Strandedness |
| --- | --- | --- | --- | --- |
| TCCAGGAGGGTTTCGGGTTGCTCTG | 563 | 967 | 1160 | Antisense |
| ATGGATACCAGATTCCCAAGGGCTG | 111 | 69 | 1201 | Antisense |
| TTAATCCTGACCGATTCATGCTGCC | 1041 | 1125 | 1291 | Antisense |
| CTGCCTCACCCAGAGGATGCATCCA | 859 | 391 | 1311 | Antisense |
| CCAGGTTCAGCTTCATTCCATTTGG | 669 | 457 | 1333 | Antisense |
| GAGGAGGCCTTAGGAGCTGTGTAGG | 576 | 659 | 1357 | Antisense |
| CAGCTTCTAAATGGACCTCCTACAA | 983 | 323 | 1446 | Antisense |
| GTCCCACCGTGTATCCTGTGGACAA | 122 | 757 | 1480 | Antisense |
| GTGGACAATCTCCCTGCAAGATTCA | 904 | 787 | 1497 | Antisense |
| CTGCAAGATTCACCCATTTCCATGG | 562 | 395 | 1510 | Antisense |
| GACTATACTACCACAATCTTTAAAT | 956 | 607 | 1661 | Antisense |

**>HG-U133_PLUS_2:266_S_AT GENE CD24**

ttcacaaacttttatactctttctgtatatacattttttttctttaaaaaacaactatggatcagaatagcaacatttagaacactttttgttatcagtcaatatttttagatagttagaacctggtcctaagcctaaaagtgggcttgattctgcagtaaatcttttacaactgcctcgacacacataaacctttttaaaaatagacactccccgaagtcttttgtttgtatggtcacacactgatgcttagatgttccagtaatctaatatggccacagtagtcttgatgaccaaagtcctttttttccatctttagaaaactacatgggaacaaacagatcgaacagttttgaagctactgtgtgtgtgaatgaacactcttgctttattccagaatgctgtacatctattttggattgtatattgtggttgtgtatttacgctttgattcatagtaacttcttatggaattgat

**Alignment (orangutan on top; human on bottom)**

000000001 ttcaca..aacttttatactctttctgtatatacatttttttt.ctttaa 000000047

<<<<<<<<< |||||| ||||||||||||||||||||||||||||||||||| |||||| <<<<<<<<<

108952862 ttcacaagaacttttatactctttctgtatatacattttttttcctttaa 108952813

000000048 aaaacaactatggatcagaatagcaacatttagaacactttttgttatca 000000097

<<<<<<<<< |||||||||||||||||||||||| |||||| |||||||||||||||||| <<<<<<<<<

108952812 aaaacaactatggatcagaatagccacatttggaacactttttgttatca 108952763

000000098 gtcaatatttttagatagttagaacctggtcctaagcctaaaagtgggct 000000147

<<<<<<<<< |||||||||||||||||||||||||||||||||||||||||||||||||| <<<<<<<<<

108952762 gtcaatatttttagatagttagaacctggtcctaagcctaaaagtgggct 108952713

000000148 tgattctgcagtaaatcttttacaactgcctcgacacacataaacctttt 000000197

<<<<<<<<< |||||||||||||||||||||||||||||||||||||||| ||||||||| <<<<<<<<<

108952712 tgattctgcagtaaatcttttacaactgcctcgacacacagaaacctttt 108952663

000000198 taaaaatagacactccccgaagtcttttgtttgtatggtcacacactgat 000000247

<<<<<<<<< ||||| ||||||||||||||||||||||||| | |||||||||||||||| <<<<<<<<<

108952662 taaaactagacactccccgaagtcttttgttcgcatggtcacacactgat 108952613

000000248 gcttagatgttccagtaatctaatatggccacagtagtcttgatgaccaa 000000297

<<<<<<<<< |||||||||||||||||||||||||||||||||||||||||||||||| | <<<<<<<<<

108952612 gcttagatgttccagtaatctaatatggccacagtagtcttgatgaccga 108952563

000000298 agtcctttttttccatctttagaaaactacatgggaacaaacagatcgaa 000000347

<<<<<<<<< |||||| ||||||||||||||||||||||||||||||||||||||| ||| <<<<<<<<<

108952562 agtcctgtttttccatctttagaaaactacatgggaacaaacagattgaa 108952513

000000348 cagttttgaagctactgtgtgtgtgaatgaacactc..ttgctttattcc 000000395

<<<<<<<<< ||||| |||||||||||||||||||||||||||||| |||||||||||| <<<<<<<<<

108952512 cagttctgaagctactgtgtgtgtgaatgaacactcttttgctttattcc 108952463

000000396 agaatgctgtacatctattttggattgtatattgtggttgtgtatttacg 000000445

<<<<<<<<< |||||||||||||||||||||||||||||||||||| ||||||||||||| <<<<<<<<<

108952462 agaatgctgtacatctattttggattgtatattgtgtttgtgtatttacg 108952413

000000446 ctttgattcatagtaacttcttat.....ggaattgat 000000478

<<<<<<<<< |||||||||||||||||||||||| ||||||||| <<<<<<<<<

108952412 ctttgattcatagtaacttcttatgttatggaattgat 108952375

| Probe Sequence(5'-3') | Probe X | Probe Y | Probe Interrogation Position | Target Strandedness |
| --- | --- | --- | --- | --- |
| TTCACAAACTTTTATACTCTTTCTG | 637 | 1127 | 1612 | Antisense |
| GTCCTAAGCCTAAAAGTGGGCTTGA | 740 | 757 | 1737 | Antisense |
| AGTGGGCTTGATTCTGCAGTAAATC | 1122 | 91 | 1751 | Antisense |
| CAGTAAATCTTTTACAACTGCCTCG | 567 | 333 | 1767 | Antisense |
| GCCTCGACACACATAAACCTTTTTA | 258 | 479 | 1786 | Antisense |
| ACACTGATGCTTAGATGTTCCAGTA | 946 | 191 | 1851 | Antisense |
| GTAATCTAATATGGCCACAGTAGTC | 1082 | 739 | 1873 | Antisense |
| CACAGTAGTCTTGATGACCAAAGTC | 802 | 309 | 1888 | Antisense |
| TTTCCATCTTTAGAAAACTACATGG | 878 | 1095 | 1918 | Antisense |
| GGAACAAACAGATCGAACAGTTTTG | 429 | 861 | 1942 | Antisense |
| CTACTGTGTGTGTGAATGAACACTC | 731 | 373 | 1970 | Antisense |
| AACACTCTTGCTTTATTCCAGAATG | 33 | 229 | 1988 | Antisense |
| TATTCCAGAATGCTGTACATCTATT | 1102 | 1087 | 2001 | Antisense |
| TGCTGTACATCTATTTTGGATTGTA | 1142 | 963 | 2011 | Antisense |
| TTTACGCTTTGATTCATAGTAACTT | 655 | 1103 | 2051 | Antisense |
| CATAGTAACTTCTTATGGAATTGAT | 3 | 351 | 2065 | Antisense |

**>HG-U133_PLUS_2:203130_S_AT GENE KIF5C**

aagatttcacttcattgtctagcccagaatcttgagcaagctaaagaaaccatcataatctaaaattgcttcatttaacactaacaatttagactttttaaaccaagcattgaataatggctggataactgccgaagtaagcgccgctccatgaagtctgcttacttatttaaaaattgtgtatcagttttaaatactgttcattgtgtgcagatataaggggaatagggcattctgtagaattatacatgtctagtttgtaaagtgtgtcctgtgtactgcagatgtgtgttctctgggctttatgtatctgtacagtagctttcacattaaaaaaattgtggacaaacttgtccggggggtttgaggggagaatggtggtttatatcaataacgatgctgtactatagtccatgtaacaaaagatctggaagtcaccctcctctggcccacggaaaattttggtaatcttctaggttctaaaatgaagatgtatgggtactctggcagactgc

**Alignment (orangutan on top; human on bottom)**

00000103 ccaagcattgaataatggctggataactgccgaagtaagcgccgctc 00000149

>>>>>>>> ||||||||||||||||||||||||||||||||||||||||||||||| >>>>>>>>

38417846 ccaagcattgaataatggctggataactgccgaagtaagcgccgctc 38417892

00000155 agtctgct 00000162

>>>>>>>> |||||||| >>>>>>>>

38417896 agtctgct 38417903

00000167 tatttaaaaattgtgtatcagttttaaatact 00000198

>>>>>>>> ||||||||||| |||||||||||| |||||| >>>>>>>>

38417906 tatttaaaaat.gtgtatcagttt..aatact 38417934

00000201 tcattgtgtgcagatataaggggaatagggcattctgtagaattatacat 00000250

>>>>>>>> |||||||||||||||||||||||||||||||||||||||||||||||||| >>>>>>>>

38417936 tcattgtgtgcagatataaggggaatagggcattctgtagaattatacat 38417985

00000251 gtctagtttgtaaagtgtgtcctgtgtactgcagatgtgtgttctctggg 00000300

>>>>>>>> |||||||| ||||||||||||||||||||||||||||||||||||||||| >>>>>>>>

38417986 gtctagtt.gtaaagtgtgtcctgtgtactgcagatgtgtgttctctggg 38418034

00000301 ctttatgtatctgtacagtagctttcacattaaaaaaattgtggacaaac 00000350

>>>>>>>> |||||||||||||||||||||||||||||||||||||||||||||||||| >>>>>>>>

38418035 ctttatgtatctgtacagtagctttcacattaaaaaaattgtggacaaac 38418084

00000351 ttgtccggggggtttgaggggagaatggtggtttatatcaataacgatgc 00000400

>>>>>>>> |||||||||||||||||||||||||||||||||||||||| ||||||||| >>>>>>>>

38418085 ttgtccggggggtttgaggggagaatggtggtttatatcagtaacgatgc 38418134

00000401 tgtactatagtccatgtaacaaaagatctggaagtcaccctcctctggcc 00000450

>>>>>>>> |||||||||||||||||||||||| ||||||||||| |||||||||||| >>>>>>>>

38418135 tgtactatagtccatgtaacaaaacgtctggaagtcatcctcctctggcc 38418184

00000451 cacggaaaattttggtaatcttctaggttctaaaatgaagatgtatgggt 00000500

>>>>>>>> |||||||||||||||||||||||||||||||||||||||||||||||||| >>>>>>>>

38418185 cacggaaaattttggtaatcttctaggttctaaaatgaagatgtatgggt 38418234

00000501 actctggcagactgc 00000515

>>>>>>>> ||||||||||||||| >>>>>>>>

38418235 actctggcagactgc 38418249

| Probe Sequence(5'-3') | Probe X | Probe Y | Probe Interrogation Position | Target Strandedness |
| --- | --- | --- | --- | --- |
| AAGATTTCACTTCATTGTCTAGCCC | 671 | 257 | 6369 | Antisense |
| GGATAACTGCCGAAGTAAGCGCCGC | 615 | 837 | 6491 | Antisense |
| CCGCTCCATGAAGTCTGCTTACTTA * | 713 | 435 | 6512 | Antisense |
| AAGTGTGTCCTGTGTACTGCAGATG | 479 | 263 | 6631 | Antisense |
| ACTGCAGATGTGTGTTCTCTGGGCT | 536 | 169 | 6646 | Antisense |
| TTCTCTGGGCTTTATGTATCTGTAC | 211 | 1137 | 6660 | Antisense |
| GTATCTGTACAGTAGCTTTCACATT | 76 | 725 | 6675 | Antisense |
| AACGATGCTGTACTATAGTCCATGT | 655 | 239 | 6761 | Antisense |
| AAAAGATCTGGAAGTCACCCTCCTC | 691 | 223 | 6789 | Antisense |
| CTCCTCTGGCCCACGGAAAATTTTG | 427 | 369 | 6808 | Antisense |
| ATGTATGGGTACTCTGGCAGACTGC | 49 | 63 | 6859 | Antisense |

*Middle of Probe Sequence (in two places) is not present in side by side alignment.

**>HG-U133_PLUS_2:209757_S_AT GENE MYCN**

cagatgccacataaggggtttgccatttgatacccctggggaacatttctgtaaataccattgacacatccgccttttgtatacatcctgggtaatgagaggtggcttttgcggccagtattagactggaagttcatacctaagtactgtaataatacctcaatgtttgaggagcatgttttgtatacaaatatattgttaatctctgttatgtactgtactaattcttacactgcctgtatactttagtatgacgctgatacataactaaatttgatacttatattttcgtatgaaaatgagttgtgaaagttttgagtagatattactttatcactttttgaactaagaaacttttgtaaagaaatttactatatatatatgcctttttcctagcctgtttcttcctgttaatgtatttgttcatgtttggtgcatagaactgggtaaatgcaaagttctgtgtttaatttcttcaaaatgtatatatttagtgctgcatctta

**Alignment (orangutan on top; human on bottom)**

00000001 cagatgccacataaggggtttgccatttgatacccctggggaacatttct 00000050

<<<<<<<< |||||||||||||||||||||||||||||||||||||||||||||||||| <<<<<<<<

96540971 cagatgccacataaggggtttgccatttgatacccctggggaacatttct 96540922

00000051 gtaaataccattgacacatccgccttttgtatacatcctgggtaatgaga 00000100

<<<<<<<< |||||||||||||||||||||||||||||||||||||||||||||||||| <<<<<<<<

96540921 gtaaataccattgacacatccgccttttgtatacatcctgggtaatgaga 96540872

00000101 ggtggcttttgcggccagtattagactggaagttcatacctaagtactgt 00000150

<<<<<<<< ||||||||||| |||||||||||||||||||||||||||||||||||||| <<<<<<<<

96540871 ggtggcttttgtggccagtattagactggaagttcatacctaagtactgt 96540822

00000151 aataatacctcaatgtttgaggagcatgttttgtatacaaatatattgtt 00000200

<<<<<<<< |||||||||||||||||||||||||||||||||||||||||||||||||| <<<<<<<<

96540821 aataatacctcaatgtttgaggagcatgttttgtatacaaatatattgtt 96540772

00000201 aatctctgttatgtactgtactaattcttacactgcctgtatactttagt 00000250

<<<<<<<< |||||||||||||||||||||||||||||||||||||||||||||||||| <<<<<<<<

96540771 aatctctgttatgtactgtactaattcttacactgcctgtatactttagt 96540722

00000251 atgacgctgatacataactaaatttgatacttatattttcgtatgaaaat 00000300

<<<<<<<< |||||||||||||||||||||||||||||||||||||||||||||||||| <<<<<<<<

96540721 atgacgctgatacataactaaatttgatacttatattttcgtatgaaaat 96540672

00000301 gagttgtgaaagttttgagtagatattactttatcactttttgaactaag 00000350

<<<<<<<< |||||||||||||||||||||||||||||||||||||||||||||||||| <<<<<<<<

96540671 gagttgtgaaagttttgagtagatattactttatcactttttgaactaag 96540622

00000351 aaacttttgtaaagaaatttactatatatatatgcctttttcctagcctg 00000400

<<<<<<<< |||||||||||||||||||||||||||||||||||||||||||||||||| <<<<<<<<

96540621 aaacttttgtaaagaaatttactatatatatatgcctttttcctagcctg 96540572

00000401 tttcttcctgttaatgtatttgttcatgtttggtgcatagaactgggtaa 00000450

<<<<<<<< |||||||||||||||||||||||||||||||||||||||||||||||||| <<<<<<<<

96540571 tttcttcctgttaatgtatttgttcatgtttggtgcatagaactgggtaa 96540522

00000451 atgcaaagttctgtgtttaatttcttcaaaatgtatatatttagtgctgc 00000500

<<<<<<<< |||||||||||||||||||||||||||||||||||||||||||||||||| <<<<<<<<

96540521 atgcaaagttctgtgtttaatttcttcaaaatgtatatatttagtgctgc 96540472

00000501 atctta 00000506

<<<<<<<< |||||| <<<<<<<<

96540471 atctta 96540466

| Probe Sequence(5'-3') | Probe X | Probe Y | Probe Interrogation Position | Target Strandedness |
| --- | --- | --- | --- | --- |
| CAGATGCCACATAAGGGGTTTGCCA | 391 | 331 | 1908 | Antisense |
| GGGGTTTGCCATTTGATACCCCTGG | 198 | 813 | 1922 | Antisense |
| TGATACCCCTGGGGAACATTTCTGT | 640 | 931 | 1935 | Antisense |
| GTAAATACCATTGACACATCCGCCT | 120 | 737 | 1958 | Antisense |
| TGACACATCCGCCTTTTGTATACAT | 117 | 949 | 1969 | Antisense |
| GAGGTGGCTTTTGCGGCCAGTATTA | 949 | 653 | 2006 | Antisense |
| AGTACTGTAATAATACCTCAATGTT | 194 | 101 | 2050 | Antisense |
| GTACTGTACTAATTCTTACACTGCC | 560 | 733 | 2120 | Antisense |
| TACACTGCCTGTATACTTTAGTATG | 874 | 1051 | 2136 | Antisense |
| TCATGTTTGGTGCATAGAACTGGGT | 537 | 1017 | 2331 | Antisense |
| TGTATATATTTAGTGCTGCATCTTA | 1088 | 927 | 2389 | Antisense |

**>HG-U133_PLUS_2:213201_S_AT GENE TNNT1**

gagcggcgccggtcagagagagccgagcaacagcgcttcagaactgagaaggaacgcgaacgtcaggctaagctggcnggaggagaagatgaggaaggaagagganagaggccaagaagcgggcagaggatgatgccaagaaaaagaaggtgctgtccaacatgggggcccattttggcggctacctggtcaaggcagaacagaagcgtggtaagcggcagacggggcgggagatgaaggtgcgcatcctctccgagcgtaagaagcctctggacattgactacatgggggaggaacagctccgggagaaagcccaggagctgtcggactggatccaccagctggagtctgagaagttcgacctgatggcgaanctgaaacagcagaaatatgagatcaacgtgctgtacaaccgcatcagccacgcccagaagttccggaagggggcagggaagggccgcgttggaggccgctggaagtgaggatgccgccccggacagtggcacctgggaagcctgggagtgtttgtcccatcggtagcttgaaataaacgctcccctc

**Alignment (orangutan on top; human on bottom)**

00000001 gagcggcgccggtcagagagagccgagcaacagcgcttcagaactgagaa 00000050

<<<<<<<< ||||||||||| || |||||||| |||||||||||||||||||||||||| <<<<<<<<

57011183 gagcggcgccgatcggagagagctgagcaacagcgcttcagaactgagaa 57011134

00000051 ggaacgcgaacgtcaggctaagctggcng 00000079

<<<<<<<< ||||||||||||||||||||||||||| | <<<<<<<<

57011133 ggaacgcgaacgtcaggctaagctggcgg 57011105

00000080 gaggagaagatgaggaaggaagagganagaggccaagaagcgggcagagg 00000129

<<<<<<<< |||||||||||||||||||||||||| ||||||||||||||||||||||| <<<<<<<<

57007984 gaggagaagatgaggaaggaagagga.agaggccaagaagcgggcagagg 57007936

00000130 atgatgccaagaaaaagaaggtgctgtccaacatgggggcccattttggc 00000179

<<<<<<<< ||||||||||||||||||||||||||||||||||||| |||||||||||| <<<<<<<<

57007935 atgatgccaagaaaaagaaggtgctgtccaacatgggagcccattttggc 57007886

00000180 ggctacctggtcaag 00000194

<<<<<<<< ||||||||||||||| <<<<<<<<

57007885 ggctacctggtcaag 57007871

00000195 gcagaacagaagcgtggtaagcggcagacggggcgggagatgaaggtgcg 00000244

<<<<<<<< |||||||||||||||||||||||||||||||||||||||||||||||||| <<<<<<<<

57004770 gcagaacagaagcgtggtaagcggcagacggggcgggagatgaaggtgcg 57004721

00000245 catcctctccgagcgtaagaagcctctggacattgactacatgggggagg 00000294

<<<<<<<< ||||||||||||||| |||||||||||||||||||||||||||||||||| <<<<<<<<

57004720 catcctctccgagcggaagaagcctctggacattgactacatgggggagg 57004671

00000295 aacagctccgg 00000305

<<<<<<<< ||||||||||| <<<<<<<<

57004670 aacagctccgg 57004660

00000394 agatcaacgtgctgtacaaccgcatcagccacgcccagaagtt 00000436

<<<<<<<< |||||||||||||||||||||||||||||| |||||||||||| <<<<<<<<

57000975 agatcaacgtgctgtacaaccgcatcagccccgcccagaagtt 57000933

00000437 ccggaagggggcagggaagggccgcgttggaggccgctggaagtgaggat 00000486

<<<<<<<< |||||||||||||||||||||||||||||||||||||||||||||||| | <<<<<<<<

57000047 ccggaagggggcagggaagggccgcgttggaggccgctggaagtgaggct 56999998

00000487 gccgccccggacagtggcacctgggaagcctgggagtgtttgtcccatcg 00000536

<<<<<<<< || ||||||||||| |||||| ||||||||||||||||||||||||||| <<<<<<<<

56999997 gctgccccggacagcggcacccgggaagcctgggagtgtttgtcccatca 56999948

00000537 gtagcttgaaataaacgctcccctc 00000561

<<<<<<<< ||||||||||||||||||||||||| <<<<<<<<

56999947 gtagcttgaaataaacgctcccctc 56999923

| Probe Sequence(5'-3') | Probe X | Probe Y | Probe Interrogation Position | Target Strandedness |
| --- | --- | --- | --- | --- |
| GAGCGGCGCCGGTCAGAGAGAGCCG | 458 | 627 | 380 | Antisense |
| AGCCGAGCAACAGCGCTTCAGAACT | 175 | 145 | 400 | Antisense |
| AACGCGAACGTCAGGCTAAGCTGGC | 401 | 235 | 432 | Antisense |
| AACATGGGGGCCCATTTTGGCGGCT | 683 | 229 | 538 | Antisense |
| GGGAGATGAAGGTGCGCATCCTCTC | 398 | 803 | 608 | Antisense |
| GCATCCTCTCCGAGCGTAAGAAGCC | 358 | 523 | 623 | Antisense |
| AGAAGCCTCTGGACATTGACTACAT | 399 | 125 | 641 | Antisense |
| GGACTGGATCCACCAGCTGGAGTCT * | 791 | 855 | 705 | Antisense |
| GATCAACGTGCTGTACAACCGCATC | 689 | 679 | 774 | Antisense |
| GCCACGCCCAGAAGTTCCGGAAGGG | 609 | 463 | 800 | Antisense |
| GTAGCTTGAAATAAACGCTCCCCTC | 985 | 727 | 916 | Antisense |

*Probe sequence not found in side by side alignment.

**>HG-U133_PLUS_2:209169_AT GENE GPM6B**

aatgcctaaaaatgcctatttgggattttttttttttttnaaattaagagaagctctcttctgtgtagaacagttgttccaaaatagcttagtgttttgttttcctgttgcatgacagatttaactattctttccagcagtggaggtgctgtcagagtccagtgttctagaagaggcagtgtctaaagcctaattttncttttctaattctggtagctattaccaggaatttttgaaagttttgtttaagtagtctaatattttttatgtaaagagcattaaattttgctatgtataaatttttgtaacctaacagtgaatcaatattttctatcagtnccaagggcttcctgtagttctattcaa

**Alignment (orangutan on top; human on bottom)**

00000001 aatgcctaaaaatgcctatttgggatttt 00000029

<<<<<<<< ||||||||||||||||||||||||||||| <<<<<<<<

13674181 aatgcctaaaaatgcctatttgggatttt 13674153

00000036 ttttnaaattaagagaagctctcttctgtgtagaacagttgttccaaaat 00000085

<<<<<<<< |||| ||||||||||||||||||||||||||||||||||||||||||||| <<<<<<<<

13674151 tttttaaattaagagaagctctcttctgtgtagaacagttgttccaaaat 13674102

00000086 agc.ttagtgttttgttttcctgttgcatgacagatttaactattctttc 00000134

<<<<<<<< ||| |||||||||||||||||||||||||||||||||||||||||||||| <<<<<<<<

13674101 agctttagtgttttgttttcctgttgcatgacagatttaactattctttc 13674052

00000135 cagcagtggaggtgctgtcagagtccagtgttctagaagaggcagtgtct 00000184

<<<<<<<< |||||||||||||||||||||||||||||||||||||||||||||||||| <<<<<<<<

13674051 cagcagtggaggtgctgtcagagtccagtgttctagaagaggcagtgtct 13674002

00000185 aaagcctaattttnc.ttttctaattctggtagctattaccaggaatttt 00000233

<<<<<<<< ||||||||||||| | |||||||||||||||||||||||||||||||||| <<<<<<<<

13674001 aaagcctaattttactttttctaattctggtagctattaccaggaatttt 13673952

00000234 tgaaagttttgtttaagtagtctaatattttttatgtaaagagcattaaa 00000283

<<<<<<<< |||||||||||||||||||||||||||||||||||||||||||||||||| <<<<<<<<

13673951 tgaaagttttgtttaagtagtctaatattttttatgtaaagagcattaaa 13673902

00000284 ttttgctatgtataaatttttgtaacctaacagtgaatcaatattttcta 00000333

<<<<<<<< |||||||||||||||||||||||||||||||||||||||||||||||||| <<<<<<<<

13673901 ttttgctatgtataaatttttgtaacctaacagtgaatcaatattttcta 13673852

00000334 tcagtnccaagggcttcc.tgta.gttctattcaa 00000366

<<<<<<<< ||||| |||||||||||| |||| ||||||||||| <<<<<<<<

13673851 tcagtgccaagggcttccctgtatgttctattcaa 13673817

| Probe Sequence(5'-3') | Probe X | Probe Y | Probe Interrogation Position | Target Strandedness |
| --- | --- | --- | --- | --- |
| AATGCCTAAAAATGCCTATTTGGGA | 315 | 261 | 4186 | Antisense |
| AGAAGCTCTCTTCTGTGTAGAACAG | 84 | 127 | 4234 | Antisense |
| CAGTTGTTCCAAAATAGCTTAGTGT | 1147 | 331 | 4256 | Antisense |
| TGTTTTGTTTTCCTGTTGCATGACA | 98 | 931 | 4278 | Antisense |
| GACAGATTTAACTATTCTTTCCAGC | 32 | 605 | 4299 | Antisense |
| TATTCTTTCCAGCAGTGGAGGTGCT | 213 | 1089 | 4311 | Antisense |
| GAGGTGCTGTCAGAGTCCAGTGTTC | 528 | 657 | 4328 | Antisense |
| GTCCAGTGTTCTAGAAGAGGCAGTG | 314 | 761 | 4342 | Antisense |
| GAGGCAGTGTCTAAAGCCTAATTTT | 1158 | 661 | 4358 | Antisense |
| GTAGCTATTACCAGGAATTTTTGAA | 540 | 729 | 4398 | Antisense |
| AAGGGCTTCCTGTAGTTCTATTCAA | 1041 | 263 | 4527 | Antisense |

**>HG-U133_PLUS_2:213721_AT GENE SOX2**

gcacatgaacggctggagcaacggcagctacagcatgatgcaggaccagctgggctacccgcagcacccgggcctcaatgcgcacggcgcagcgcagatgcagcccatgcaccgctacgacgtgagcgccctgcagtacaactccatgaccagctcgcagacctacatgaacggntcgcccacctacagcatgtcctactcgcagcagggcacccctggcatggctcttggctccatgggttcggtggtcaagtccgaggccagctccagcccccctgtnggttacctcttcctcccactccagggcgccctgccaggccggggacctccgggacatgatcagcatgtatctccccggcgccgaggtgccggaacccgccgcccccagcagacttcacatgtcccagcactaccagagcggcccggtgcccngcacnggccattaacggcacactgcccctctcacacatgtgagggccggacagcgaactggaggggggagaaattttcaaagaaaaacgagggaaatgggaggggtgcaaaagaggagagtaagaaacagcatggagaaaacccggtacgctca

**Alignment (orangutan on top; human on bottom)**

000000001 gcacatgaacggctggagcaacggcagctacagcatgatgcaggaccagc 000000050

>>>>>>>>> |||||||||||||||||||||||||||||||||||||||||||||||||| >>>>>>>>>

185446901 gcacatgaacggctggagcaacggcagctacagcatgatgcaggaccagc 185446950

000000051 tgggctacccgcagcacccgggcctcaatgcgcacggcgcagcgcagatg 000000100

>>>>>>>>> |||||||||||||||||||||||||||||||||||||||||||||||||| >>>>>>>>>

185446951 tgggctacccgcagcacccgggcctcaatgcgcacggcgcagcgcagatg 185447000

000000101 cagcccatgcaccgctacgacgtgagcgccctgcagtacaactccatgac 000000150

>>>>>>>>> |||||||||||||||||||||||||||||||||||||||||||||||||| >>>>>>>>>

185447001 cagcccatgcaccgctacgacgtgagcgccctgcagtacaactccatgac 185447050

000000151 cagctcgcagacctacatgaacggntcgcccacctacagcatgtcctact 000000200

>>>>>>>>> |||||||||||||||||||||||| ||||| ||||||||||||||||||| >>>>>>>>>

185447051 cagctcgcagacctacatgaacggctcgccaacctacagcatgtcctact 185447100

000000201 cgcagcagggcacccctggcatggctcttggctccatgggttcggtggtc 000000250

>>>>>>>>> |||||||||||||||||||||||||||||||||||||||||||||||||| >>>>>>>>>

185447101 cgcagcagggcacccctggcatggctcttggctccatgggttcggtggtc 185447150

000000251 aagtccgaggccagctccagcccccctgtnggttacctcttcctcccact 000000300

>>>>>>>>> ||||||||||||||||||||||||||||| |||||||||||||||||||| >>>>>>>>>

185447151 aagtccgaggccagctccagcccccctgt.ggttacctcttcctcccact 185447199

000000301 ccagggcgccctgccaggccggggacctccgggacatgatcagcatgtat 000000350

>>>>>>>>> |||||||||||||||||||||||||||||||||||||||||||||||||| >>>>>>>>>

185447200 ccagggcgccctgccaggccggggacctccgggacatgatcagcatgtat 185447249

000000351 ctccccggcgccgaggtgccggaacccgccgcccccagcagacttcacat 000000400

>>>>>>>>> |||||||||||||||||||||||||||||||||||||||||||||||||| >>>>>>>>>

185447250 ctccccggcgccgaggtgccggaacccgccgcccccagcagacttcacat 185447299

000000401 gtcccagcactaccagagcggcccggtgcccngcacnggccattaacggc 000000450

>>>>>>>>> ||||||||||||||||||||||||||||||| |||| ||||||||||||| >>>>>>>>>

185447300 gtcccagcactaccagagcggcccggtgcccggcac.ggccattaacggc 185447348

000000451 acactgcccctctcacacatgtgagggccggacagcgaactggagggggg 000000500

>>>>>>>>> |||||||||||||||||||||||||||||||||||||||||||||||||| >>>>>>>>>

185447349 acactgcccctctcacacatgtgagggccggacagcgaactggagggggg 185447398

000000501 agaaattttcaaagaaaaacgagggaaatgggaggggtgcaaaagaggag 000000550

>>>>>>>>> |||||||||||||||||||||||||||||||||||||||||||||||||| >>>>>>>>>

185447399 agaaattttcaaagaaaaacgagggaaatgggaggggtgcaaaagaggag 185447448

000000551 agtaagaaacagcatggagaaaacccggtacgctca 000000586

>>>>>>>>> |||||||||||||||||||||||||||||||||||| >>>>>>>>>

185447449 agtaagaaacagcatggagaaaacccggtacgctca 185447484

| Probe Sequence(5'-3') | Probe X | Probe Y | Probe Interrogation Position | Target Strandedness |
| --- | --- | --- | --- | --- |
| GCACATGAACGGCTGGAGCAACGGC | 1133 | 537 | 942 | Antisense |
| CTGGAGCAACGGCAGCTACAGCATG | 237 | 407 | 954 | Antisense |
| GCTACAGCATGATGCAGGACCAGCT | 532 | 509 | 968 | Antisense |
| CCTGCAGTACAACTCCATGACCAGC | 1123 | 441 | 1071 | Antisense |
| CCAGCTCGCAGACCTACATGAACGG | 1062 | 459 | 1091 | Antisense |
| GCTCCATGGGTTCGGTGGTCAAGTC | 45 | 505 | 1172 | Antisense |
| TTCGGTGGTCAAGTCCGAGGCCAGC | 42 | 1139 | 1182 | Antisense |
| CTGCCCCTCTCACACATGTGAGGGC | 80 | 393 | 1395 | Antisense |
| CACATGTGAGGGCCGGACAGCGAAC | 551 | 311 | 1407 | Antisense |
| GCCGGACAGCGAACTGGAGGGGGGA | 198 | 483 | 1418 | Antisense |
| GCATGGAGAAAACCCGGTACGCTCA | 564 | 525 | 1503 | Antisense |

**>HG-U133_PLUS_2:207183_AT GENE GPR19**

tcatgtagctcagctatggcacccccatgaacaagactataagaaaagttcccttgttttcacagctatcacatggatatcctttagttcttcagcctctaaacctactctgtattcaatttataatgccaattttcggagagggatgaaagagactttttgcatgtcctctatgaaatgttaccgaagcaatgcctatactatcacaacaagttcaaggatggccaaaaaaaactacgttggcatttcagaaatcccttccatggccaaaactattaccaaagactcgatctatgactcatttgacagagaagccaaggaaaaaaagcttgcttggcccattaactcaaatccaccaaatacttttgtctaagttctcattctttcaattgttatgcaccagagattaaaaagctttaactataaaaacagaagctatttacatatttgttttcactcaactttccaaggg

**Alignment (orangutan on top; human on bottom)**

00000001 tcatgtagctcagctatggcacccccatgaacaagactataagaaaagtt 00000050

<<<<<<<< ||||||||||||||||||||||||||||||||||||||||||||| |||| <<<<<<<<

13095931 tcatgtagctcagctatggcacccccatgaacaagactataagaagagtt 13095882

00000051 cccttgttttcacagctatcacatggatatcctttagttcttcagcctct 00000100

<<<<<<<< |||||||||||||||||||||||||||||||||||||||||||||||||| <<<<<<<<

13095881 cccttgttttcacagctatcacatggatatcctttagttcttcagcctct 13095832

00000101 aaacctactctgtattcaatttataatgccaattttcggagagggatgaa 00000150

<<<<<<<< |||||||||||||||||||||||||||||||||||||||||||||||||| <<<<<<<<

13095831 aaacctactctgtattcaatttataatgccaattttcggagagggatgaa 13095782

00000151 agagactttttgcatgtcctctatgaaatgttaccgaagcaatgcctata 00000200

<<<<<<<< |||||||||||||||||||||||||||||||||||||||||||||||||| <<<<<<<<

13095781 agagactttttgcatgtcctctatgaaatgttaccgaagcaatgcctata 13095732

00000201 ctatcacaacaagttcaaggatggccaaaaaaaactacgttggcatttca 00000250

<<<<<<<< |||||||||| ||||||||||||||||||||||||||||||||||||||| <<<<<<<<

13095731 ctatcacaaccagttcaaggatggccaaaaaaaactacgttggcatttca 13095682

00000251 gaaatcccttccatggccaaaactattaccaaagactcgatctatgactc 00000300

<<<<<<<< |||||||||||||||||||||||||||||||||||||||||||||||||| <<<<<<<<

13095681 gaaatcccttccatggccaaaactattaccaaagactcgatctatgactc 13095632

00000301 atttgacagagaagccaaggaaaaaaagcttgcttggcccattaactcaa 00000350

<<<<<<<< |||||||||||||||||||||||||||||||||||||||||||||||||| <<<<<<<<

13095631 atttgacagagaagccaaggaaaaaaagcttgcttggcccattaactcaa 13095582

00000351 atccaccaaatacttttgtctaagttctcattctttcaattgttatgcac 00000400

<<<<<<<< |||||||||||||||||||||||||||||||||||||||||||||||||| <<<<<<<<

13095581 atccaccaaatacttttgtctaagttctcattctttcaattgttatgcac 13095532

00000401 cagagattaaaaagctttaactataaaaacagaagctatttacatatttg 00000450

<<<<<<<< |||||||||||||||||||||||||||||||||||||||||||||||||| <<<<<<<<

13095531 cagagattaaaaagctttaactataaaaacagaagctatttacatatttg 13095482

00000451 ttttcactcaactttccaaggg 00000472

<<<<<<<< ||||||||||||||||||||| <<<<<<<<

13095481 ctttcactcaactttccaaggg 13095460

| Probe Sequence(5'-3') | Probe X | Probe Y | Probe Interrogation Position | Target Strandedness |
| --- | --- | --- | --- | --- |
| TCATGTAGCTCAGCTATGGCACCCC | 716 | 1019 | 910 | Antisense |
| GCACCCCCATGAACAAGACTATAAG | 407 | 537 | 928 | Antisense |
| TTCCCTTGTTTTCACAGCTATCACA | 643 | 1141 | 958 | Antisense |
| ATCCTTTAGTTCTTCAGCCTCTAAA | 167 | 51 | 988 | Antisense |
| GCCTCTAAACCTACTCTGTATTCAA | 297 | 477 | 1004 | Antisense |
| GAAGCAATGCCTATACTATCACAAC | 851 | 569 | 1095 | Antisense |
| AAATCCCTTCCATGGCCAAAACTAT | 330 | 1 | 1161 | Antisense |
| AAAAGCTTGCTTGGCCCATTAACTC | 77 | 223 | 1233 | Antisense |
| GCCCATTAACTCAAATCCACCAAAT | 1066 | 487 | 1246 | Antisense |
| TTTTGTCTAAGTTCTCATTCTTTCA | 330 | 1113 | 1273 | Antisense |
| TTGTTTTCACTCAACTTTCCAAGGG | 337 | 1155 | 1357 | Antisense |

**>HG-U133_PLUS_2:213924_AT GENE MPPE1**

agaacctgtcgtaccagcatcatgagctggatgcaggagcccatggctgaaaggagttaaaacgcccagtggtcattaagtgaaacatcttttatcaacctgcaaaagctgcagcgttctctgccaggtcaaatgggcatgtttagaaaataagagaagatggctgagtatagctaatgaataaatggttgtttctttagaaaattaaacacacacagagtgtaagaggagaggatacggccctccctgaaggataaagtccncctggacggtgccctgccctcgcttctcacattaactgcccaggaatgtcatgctgattggttcccggaagggtgtttggcaaggggcagtgtatggagctacgtgtagaaggagagaaatttgtgtgtggcttttgtaaattttgaccgattgcagcaat

**Alignment (orangutan on top; human on bottom)**

00000001 agaacctgtcgtaccagcatcatgagctggatgcaggagcccatggctga 00000050

<<<<<<<< |||||||||||||||||||||||||||||||||||||||||||||||||| <<<<<<<<

20114914 agaacctgtcgtaccagcatcatgagctggatgcaggagcccatggctga 20114865

00000051 aaggagtt.aaaacgcccagtggtcattaagtg.aaacatcttttatcaa 00000098

<<<<<<<< |||||||| |||||||||||||||||||||||| |||||||||||||||| <<<<<<<<

20114864 aaggagttaaaaacgcccagtggtcattaagtgaaaacatcttttatcaa 20114815

00000099 cctgcaaaagctgcagcgttctctgccaggtcaaatgggcatgtttagaa 00000148

<<<<<<<< |||| |||||||| |||||||||||||||||||||||||||||||||||| <<<<<<<<

20114814 cctgtaaaagctgtagcgttctctgccaggtcaaatgggcatgtttagaa 20114765

00000149 aataagagaagatggctgagtatagctaatgaataaatggttgtttcttt 00000198

<<<<<<<< |||||||||||||||||||||||||||||||||||||||||||||||||| <<<<<<<<

20114764 aataagagaagatggctgagtatagctaatgaataaatggttgtttcttt 20114715

00000199 agaaaattaaacacacacagagtgtaagaggagaggatacggccctccct 00000248

<<<<<<<< ||||||||||||||||||||||||||||||||||||| ||| |||||||| <<<<<<<<

20114714 agaaaattaaacacacacagagtgtaagaggagaggacacgtccctccct 20114665

00000249 gaaggataaagtccncctggacggtgccctgccctcgcttctcacattaa 00000298

<<<<<<<< ||||||||||| || ||||||||||||||||||||||||||||||||||| <<<<<<<<

20114664 gaaggataaaggccacctggacggtgccctgccctcgcttctcacattaa 20114615

00000299 ctgcccaggaatgtcatgctgattggttcccggaagggtgtttggcaagg 00000348

<<<<<<<< ||||||||||| |||||||||||||||||||||||||||||||||||| | <<<<<<<<

20114614 ctgcccaggaacgtcatgctgattggttcccggaagggtgtttggcaacg 20114565

00000349 ggcagtgtatggagctacgtgtagaaggagagaaatttgtgtgtggcttt 00000398

<<<<<<<< ||||||||||||| ||||||||||| |||| ||||||||||||||||||| <<<<<<<<

20114564 ggcagtgtatggaactacgtgtagagggagggaaatttgtgtgtggcttt 20114515

00000399 tgtaaattttgaccgattgcagcaat 00000424

<<<<<<<< |||||||||| || |||||||||||| <<<<<<<<

20114514 tgtaaatttttacagattgcagcaat 20114489

| Probe Sequence(5'-3') | Probe X | Probe Y | Probe Interrogation Position | Target Strandedness |
| --- | --- | --- | --- | --- |
| AGAACCTGTCGTACCAGCATCATGA | 759 | 125 | 999 | Antisense |
| GGAGTTAAAACGCCCAGTGGTCATT | 844 | 845 | 1051 | Antisense |
| GAAACATCTTTTATCAACCTGCAAA | 536 | 587 | 1080 | Antisense |
| GCGTTCTCTGCCAGGTCAAATGGGC | 405 | 493 | 1112 | Antisense |
| TACGGCCCTCCCTGAAGGATAAAGT | 725 | 1055 | 1234 | Antisense |
| CATTAACTGCCCAGGAATGTCATGC | 407 | 353 | 1291 | Antisense |
| GCTGATTGGTTCCCGGAAGGGTGTT | 537 | 497 | 1314 | Antisense |
| GAAGGGTGTTTGGCAAGGGGCAGTG | 1039 | 561 | 1329 | Antisense |
| GGGGCAGTGTATGGAGCTACGTGTA | 53 | 815 | 1345 | Antisense |
| AGGAGAGAAATTTGTGTGTGGCTTT | 166 | 81 | 1372 | Antisense |
| GTAAATTTTGACCGATTGCAGCAAT | 100 | 737 | 1398 | Antisense |

**>HG-U133_PLUS_2:206012_AT GENE LEFTY2**

tcagctgggagtttctgttctctggcaaattcttcactgagtctggaacaataataccctatgattagaactggggaaacagaactgaattgctgtgttatatgaggaattaaaaccttcaaatctctatttcccccaaatactgacccattctggacttttgtaaacatacctaggcccctgttcccctgagagggtgctaagaggaaggatgaagggcttcaggctgggggcagtggacagggaattgggatacctggattctggttctgacagggccacaagctaggatctctaacaaacgcagaaggctttggctcgtcatttcctcttaaaaaaggaggagctgggcttcagctctaagaacttcattgccctggggatcagacagcccctacctacccctgcccactcctctggagactgagccttgcccgtgcatatttaggtcatttcccacactgtcttagagaacttgtcaccagaaaccacatgt

**Alignment (orangutan on top; human on bottom)**

00000001 tcagctgggagtttctgttctctggcaaattcttcactgagtctggaaca 00000050

>>>>>>>> |||||||||||||||||||||||||||||||| ||||||||||||||||| >>>>>>>>

23731183 tcagctgggagtttctgttctctggcaaattcctcactgagtctggaaca 23731232

00000051 ataataccctatgattagaactggggaaacagaactgaattgctgtgtta 00000100

>>>>>>>> |||||||||||||||||||||||||||||||||||||||||||| | ||| >>>>>>>>

23731233 ataataccctatgattagaactggggaaacagaactgaattgctctatta 23731282

00000101 tatgaggaattaaaaccttcaaatctctatttccccc.aaatactgaccc 00000149

>>>>>>>> ||||||||||||||||||||||||||||||||||||| |||||||||||| >>>>>>>>

23731283 tatgaggaattaaaaccttcaaatctctatttccccccaaatactgaccc 23731332

00000150 attctggacttttgtaaacatacctaggcccctgttcccctgagagggtg 00000199

>>>>>>>> |||||||||||||||||||||||||||||||||||||||||||||||||| >>>>>>>>

23731333 attctggacttttgtaaacatacctaggcccctgttcccctgagagggtg 23731382

00000200 ctaagaggaaggatgaagggcttcaggctgggggcagtggacagggaatt 00000249

>>>>>>>> |||||||||||||||||||||||||||| || |||||||||||||||||| >>>>>>>>

23731383 ctaagaggaaggatgaagggcttcaggccggtggcagtggacagggaatt 23731432

00000250 gggatacctggattctggttctgacagggccacaagctaggatctctaac 00000299

>>>>>>>> ||||| |||||||||||||||||||||||||||||||||||||||| ||| >>>>>>>>

23731433 gggatgcctggattctggttctgacagggccacaagctaggatctcaaac 23731482

00000300 aaacgcagaaggctttggctcgtcatttcctcttaaaaaaggaggagctg 00000349

>>>>>>>> |||||||||||||||||| |||||||||||||||||||| |||||||||| >>>>>>>>

23731483 aaacgcagaaggctttgggtcgtcatttcctcttaaaaa.ggaggagctg 23731531

00000350 ggcttcagctctaagaacttcattgccctggggatcagacagcccctacc 00000399

>>>>>>>> |||||||||||||||||||||||||||||||||||||||||||||||||| >>>>>>>>

23731532 ggcttcagctctaagaacttcattgccctggggatcagacagcccctacc 23731581

00000400 tacccctgcccactcctctggagactgagccttgcccgtgcatatttagg 00000449

>>>>>>>> ||||||||| ||| |||||||||||||||||||||| ||||||||||||| >>>>>>>>

23731582 tacccctgctcacccctctggagactgagccttgcctgtgcatatttagg 23731631

00000450 tcatttcccacactgtcttagagaacttgtcaccagaaaccacatgt 00000496

>>>>>>>> ||||||||||||||||||||||||||||||||||||||||||||||| >>>>>>>>

23731632 tcatttcccacactgtcttagagaacttgtcaccagaaaccacatgt 23731678

| Probe Sequence(5'-3') | Probe X | Probe Y | Probe Interrogation Position | Target Strandedness |
| --- | --- | --- | --- | --- |
| TCAGCTGGGAGTTTCTGTTCTCTGG | 515 | 1025 | 1406 | Antisense |
| TGTTCTCTGGCAAATTCTTCACTGA | 728 | 931 | 1421 | Antisense |
| TTTTGTAAACATACCTAGGCCCCTG | 833 | 1107 | 1564 | Antisense |
| CCCTGTTCCCCTGAGAGGGTGCTAA | 237 | 427 | 1584 | Antisense |
| GGGATACCTGGATTCTGGTTCTGAC | 491 | 805 | 1655 | Antisense |
| GACAGGGCCACAAGCTAGGATCTCT | 585 | 605 | 1677 | Antisense |
| AACGCAGAAGGCTTTGGCTCGTCAT | 1112 | 239 | 1706 | Antisense |
| TCTAAGAACTTCATTGCCCTGGGGA | 615 | 1007 | 1764 | Antisense |
| GAGCCTTGCCCGTGCATATTTAGGT | 197 | 623 | 1831 | Antisense |
| ATATTTAGGTCATTTCCCACACTGT | 214 | 23 | 1846 | Antisense |
| GAACTTGTCACCAGAAACCACATGT | 938 | 579 | 1877 | Antisense |

**>HG-U133_PLUS_2:220184_AT GENE NANOG**

gacattttaataaccttggctgctaaggacaacattgatagaagccgtctctggctatagataagtagatctaatactagtttggatatctttagggtttagaatctaacctcaagaataagaaatacaagtacgaattggtgatgaagatgtattcgtattgtttgggattgggaggctttgcttatttttttaaaactattgaggtaaagggttaagctgtaacatacttaattgatttcttaccgtttttggctctgttttgctatatcccctaatttgttggttgtgctaatctttgtagaaagaggtcttgtatttgctgcatcgtaatgacatgagtactactttagttggtttaagttcaaatgaatgaaacaaatatttttcctttagttgattttaccctgatttcaccgagt

**Alignment (orangutan on top; human on bottom)**

0000001 gacattttaataaccttggctgctaaggacaacattgatagaagccgtct 0000050

>>>>>>> ||||||||||||||||||||||||||||||||||||||||||||| | || >>>>>>>

8147787 gacattttaataaccttggctgctaaggacaacattgatagaagctgact 8147836

0000051 ctggctatagataagtagatctaatactagtttggatatctttagggttt 0000100

>>>>>>> |||||||||||||||||||||||||||||||||||||||||||||||||| >>>>>>>

8147837 ctggctatagataagtagatctaatactagtttggatatctttagggttt 8147886

0000101 agaatctaacctcaagaataagaaatacaagtacgaattg..gtgatgaa 0000148

>>>>>>> |||||||||||||||||||||||||||||||||| ||||| |||||||| >>>>>>>

8147887 agaatctaacctcaagaataagaaatacaagtacaaattggtgtgatgaa 8147936

0000149 gatgtattcgtattgtttgggattgggaggctttgcttatttttttaaaa 0000198

>>>>>>> ||| ||||| ||||||||||||||||||||||||||||||||||| |||| >>>>>>>

8147937 gatatattcctattgtttgggattgggaggctttgcttattttttaaaaa 8147986

0000199 ctattgaggtaaagggttaagctgtaacatacttaattgattt.cttacc 0000247

>>>>>>> ||||||||||||||||||||||||||||||||||||||||||| ||||| >>>>>>>

8147987 ctattgaggtaaagggttaagctgtaacatacttaattgattttcttact 8148036

0000248 gtttttggctctgttttgctatatcccctaatttgttggttgtgctaatc 0000297

>>>>>>> ||||||||||||||||| |||||||||||| |||||||||| |||||||| >>>>>>>

8148037 gtttttggctctgttttactatatcccctagtttgttggttatgctaatc 8148086

0000298 tttgtagaaagaggtcttgtatttgctgcatcgtaatgacatgagtacta 0000347

>>>>>>> ||||||||||||||||| ||||||| ||||| ||||||||| ||||| | >>>>>>>

8148087 tttgtagaaagaggtctcatatttgccgcatcataatgacattagtacga 8148136

0000348 ctttagttggtttaagttcaaatgaatgaaacaaatatttttcctttagt 0000397

>>>>>>> |||||||||||||||||||||||||||||||||| ||||||||||||||| >>>>>>>

8148137 ctttagttggtttaagttcaaatgaatgaaacaactatttttcctttagt 8148186

0000398 tgattttaccctgatttcaccgagt 0000422

>>>>>>> ||||||||||||||||||||| ||| >>>>>>>

8148187 tgattttaccctgatttcacctagt 8148211

| Probe Sequence(5'-3') | Probe X | Probe Y | Probe Interrogation Position | Target Strandedness |
| --- | --- | --- | --- | --- |
| GACATTTTAATAACCTTGGCTGCTA | 937 | 607 | 1674 | Antisense |
| AACATTGATAGAAGCCGTCTCTGGC | 1109 | 231 | 1704 | Antisense |
| GCCGTCTCTGGCTATAGATAAGTAG | 702 | 483 | 1717 | Antisense |
| TTACCGTTTTTGGCTCTGTTTTGCT | 124 | 1121 | 1916 | Antisense |
| TTTGGCTCTGTTTTGCTATATCCCC | 773 | 1089 | 1924 | Antisense |
| GCTATATCCCCTAATTTGTTGGTTG | 62 | 513 | 1938 | Antisense |
| TTTGTTGGTTGTGCTAATCTTTGTA | 53 | 1093 | 1952 | Antisense |
| AGAGGTCTTGTATTTGCTGCATCGT | 348 | 113 | 1980 | Antisense |
| GCTGCATCGTAATGACATGAGTACT | 109 | 503 | 1995 | Antisense |
| TTCCTTTAGTTGATTTTACCCTGAT | 593 | 1143 | 2061 | Antisense |
| TGATTTTACCCTGATTTCACCGAGT | 475 | 933 | 2071 | Antisense |

**>HG-U133_PLUS_2:201578_AT GENE PODXL**

Cacagactgtgcgcctcagaaggaataatcggtaaattaagaattgctactcgaaggtgccagaatgacacaaaggacagaattcctttcccagttgttaccctagcaaggctagggagggcatgaacacaaacataagaactggtcttctcacactttctctgaatcatttaggtttaagatgtaagtgaacaattctttctttctgccaagaaacaaagttttggatgagcttttatatatggaacttactccaacaggactgagggaccaaggaaacatgatgggggaggcaagagagggcaaagagtaaaactgtagcatagcttttgtcacggtcactagctgatccctcaggtctgctgcaaacacagcatggaggacacagatgactctttggtgttggtctttttgtctgcagtgaatgttcaacagtttgcccaggaactgggggatcatatatgtcttagtggacaggggtctgaagtacactgga

**Alignment (orangutan on top; human on bottom)**

000000001 cacagactgtgcgcctcagaaggaataatcggtaaattaagaattgctac 000000050

<<<<<<<<< |||||||||||| ||||||||||||||||| ||||||||||||||||||| <<<<<<<<<

128509771 cacagactgtgcacctcagaaggaataatcagtaaattaagaattgctac 128509722

000000051 tcgaaggtgccagaatgacacaaaggacagaattcctttcccagttgtta 000000100

<<<<<<<<< ||||||||||||||||||||||||||||||||||||||||||| || ||| <<<<<<<<<

128509721 tcgaaggtgccagaatgacacaaaggacagaattcctttcccatttctta 128509672

000000101 ccctagcaaggctagggagggcatgaacacaaacataagaactggtcttc 000000150

<<<<<<<<< |||||||||||||||||||||||||||||||||||||||||||||||||| <<<<<<<<<

128509671 ccctagcaaggctagggagggcatgaacacaaacataagaactggtcttc 128509622

000000151 tcacactttctctgaatcatttaggtttaagatgtaagtgaacaattctt 000000200

<<<<<<<<< | |||||| |||||||||||||||||||||||||||||||||||||||| <<<<<<<<<

128509621 t.acactt..tctgaatcatttaggtttaagatgtaagtgaacaattctt 128509575

000000201 tctttctgccaagaaacaaagttttggatgagcttttatatatggaactt 000000250

<<<<<<<<< ||| |||||||||| ||||||||||||||||||||| |||||||||| <<<<<<<<<

128509574 tct....gccaagaaac.aagttttggatgagcttttatgtatggaactt 128509530

000000251 actccaacaggactgagggaccaaggaaacatgatgggggaggcaagaga 000000300

<<<<<<<<< | ||||||||||| ||||||||||||||||| ||||||||||||| |||| <<<<<<<<<

128509529 attccaacaggaccgagggaccaaggaaacaagatgggggaggca.gaga 128509481

000000301 gggcaaagagtaaaactgtagcatagcttttgtcacggtcactagctgat 000000350

<<<<<<<<< |||| |||||||||||||||||||||||||||||||||||| |||||||| <<<<<<<<<

128509480 gggc.aagagtaaaactgtagcatagcttttgtcacggtcagtagctgat 128509432

000000351 ccctcaggtctgctgcaaacacagcatggaggacacagatgactctttgg 000000400

<<<<<<<<< ||||||||||||||||||||||| |||||||||| |||||||||||| | <<<<<<<<<

128509431 ccctcaggtctgctgcaaacacaacatggaggacgcagatgactcttcag 128509382

000000401 tgttggtctttttgtctgcagtgaatgttcaacagtttgcccaggaactg 000000450

<<<<<<<<< ||| |||||||||||||||||||||||||||||||||||||||||||||| <<<<<<<<<

128509381 tgtgggtctttttgtctgcagtgaatgttcaacagtttgcccaggaactg 128509332

000000451 ggggatcatatatgtcttagtggacaggggtctgaagtacactgga 000000496

<<<<<<<<< ||||||||||| |||||||||||||||||||||||||||||||||| <<<<<<<<<

128509331 ggggatcatatgtgtcttagtggacaggggtctgaagtacactgga 128509286

| Probe Sequence(5'-3') | Probe X | Probe Y | Probe Interrogation Position | Target Strandedness |
| --- | --- | --- | --- | --- |
| CACAGACTGTGCGCCTCAGAAGGAA | 299 | 309 | 5289 | Antisense |
| GAATTGCTACTCGAAGGTGCCAGAA | 947 | 547 | 5329 | Antisense |
| CCCAGTTGTTACCCTAGCAAGGCTA | 333 | 423 | 5378 | Antisense |
| TAAGAACTGGTCTTCTCACACTTTC | 1118 | 1039 | 5424 | Antisense |
| CTTCTCACACTTTCTCTGAATCATT | 683 | 361 | 5435 | Antisense |
| TACTCCAACAGGACTGAGGGACCAA | 788 | 1057 | 5538 | Antisense |
| GTAGCATAGCTTTTGTCACGGTCAC | 1101 | 729 | 5606 | Antisense |
| TTTTGTCACGGTCACTAGCTGATCC | 427 | 1111 | 5616 | Antisense |
| TGGAGGACACAGATGACTCTTTGGT | 650 | 903 | 5665 | Antisense |
| TTTGGTGTTGGTCTTTTTGTCTGCA | 505 | 1091 | 5684 | Antisense |
| GGACAGGGGTCTGAAGTACACTGGA | 489 | 849 | 5760 | Antisense |

**>HG-U133_PLUS_2:210198_S_AT GENE PLP1**

ttaattagttgtgtactctggcctctgtcatatcttcacaatggtgctcatttcatggggtattatccattcagtcatcgtaggtgatttgaaggtcttgatttgttttagaatgatgcacatttcatgtattccagtttgtttattacttatttggggttgcatcagaaatgtctggagaataattctttgattatgactgttttttaaactaggaaaattggacattaagcatcacaaatgatattaaaaattggctagttgaatctattgggattttctacaagtattctgcctttgcagaaacagatttggtgaatttgaatctcaatttgagtaatctgatcgttctttctagctaatggaaaatgattttacttagcaatgttatcttggtgtgttaagagttaggtttaacataaaggttattttctcctgatatagatcacataacagaatgcaccagtcatcagctattcagttggtaagcttccaggaaaaaggacaggcagaaagagtttgagacctgaatagctcccagatttcagtcttttc

**Alignment (orangutan on top; human on bottom)**

000000001 ttaattagttgtgtactctggcctctgtcatatcttcacaatggtgctca 000000050

>>>>>>>>> |||||||||||||||||||||||||||||||||||||||||||||||||| >>>>>>>>>

102745060 ttaattagttgtgtactctggcctctgtcatatcttcacaatggtgctca 102745109

000000051 tttcatggggtattatccattcagtcatcgtaggtgatttgaaggtcttg 000000100

>>>>>>>>> |||||||||||||||||||||||||||| ||||||||||||||||||||| >>>>>>>>>

102745110 tttcatggggtattatccattcagtcattgtaggtgatttgaaggtcttg 102745159

000000101 atttgttttagaatgatgcacatttcatgtattccagtttgtttattact 000000150

>>>>>>>>> ||||||||||||||||||||||||| ||||||||||||||||||||||| >>>>>>>>>

102745160 atttgttttagaatgatgcacattttgtgtattccagtttgtttattact 102745209

000000151 tatttggggttgcatcagaaatgtctggagaataattctttgattatgac 000000200

>>>>>>>>> |||||||||||||||||||||||||||||||||||||||||||||||||| >>>>>>>>>

102745210 tatttggggttgcatcagaaatgtctggagaataattctttgattatgac 102745259

000000201 tgttttttaaactaggaaaattggacattaagcatcacaaatgatattaa 000000250

>>>>>>>>> | ||||||||| ||||||||||||||||||| |||||||||||||||||| >>>>>>>>>

102745260 tcttttttaaaataggaaaattggacattaaacatcacaaatgatattaa 102745309

000000251 aaattggctagtt....gaatctattgggattttctacaagtattctgcc 000000296

>>>>>>>>> ||||||||||||| ||||||||||||||||||||||||||||||||| >>>>>>>>>

102745310 aaattggctagttagatgaatctattgggattttctacaagtattctgcc 102745359

000000297 tttgcagaaacagatttggtgaatttgaatctcaatttgagtaatctgat 000000346

>>>>>>>>> |||||||||||||||||||||||||||||||||||||||||||||| ||| >>>>>>>>>

102745360 tttgcagaaacagatttggtgaatttgaatctcaatttgagtaatccgat 102745409

000000347 cgttctttctagctaatggaaaatgattttacttagcaatgttatcttgg 000000396

>>>>>>>>> |||||||||||||||||||||||||||||||||||||||||||||||||| >>>>>>>>>

102745410 cgttctttctagctaatggaaaatgattttacttagcaatgttatcttgg 102745459

000000397 tgtgttaagagttaggtttaacataaaggttattttctcctgatatagat 000000446

>>>>>>>>> |||||||||||||||||||||||||||||||||||||||||||||||||| >>>>>>>>>

102745460 tgtgttaagagttaggtttaacataaaggttattttctcctgatatagat 102745509

000000447 cacataacagaatgcaccagtcatcagctattcagttggtaagcttccag 000000496

>>>>>>>>> |||||||||||||||||||||||||||||||||||||||||||||||||| >>>>>>>>>

102745510 cacataacagaatgcaccagtcatcagctattcagttggtaagcttccag 102745559

000000497 gaaaaaggacaggcagaaagagtttgagacctgaatagctcccagatttc 000000546

>>>>>>>>> |||||||||||||||||||||||||||||||||||||||||||||||||| >>>>>>>>>

102745560 gaaaaaggacaggcagaaagagtttgagacctgaatagctcccagatttc 102745609

000000547 agtcttttc 000000555

>>>>>>>>> ||||||||| >>>>>>>>>

102745610 agtcttttc 102745618

| Probe Sequence(5'-3') | Probe X | Probe Y | Probe Interrogation Position | Target Strandedness |
| --- | --- | --- | --- | --- |
| TTAATTAGTTGTGTACTCTGGCCTC | 932 | 1121 | 1832 | Antisense |
| AATGGTGCTCATTTCATGGGGTATT | 704 | 271 | 1871 | Antisense |
| GGTATTATCCATTCAGTCATCGTAG | 438 | 829 | 1890 | Antisense |
| GAATGATGCACATTTCATGTATTCC | 428 | 557 | 1942 | Antisense |
| ATTACTTATTTGGGGTTGCATCAGA | 595 | 11 | 1976 | Antisense |
| TTTTCTACAAGTATTCTGCCTTTGC | 946 | 1107 | 2108 | Antisense |
| GTAATCTGATCGTTCTTTCTAGCTA | 300 | 741 | 2168 | Antisense |
| GTTATTTTCTCCTGATATAGATCAC | 580 | 711 | 2256 | Antisense |
| CAGTCATCAGCTATTCAGTTGGTAA | 175 | 335 | 2295 | Antisense |
| GTTTGAGACCTGAATAGCTCCCAGA | 948 | 717 | 2349 | Antisense |
| ATAGCTCCCAGATTTCAGTCTTTTC | 454 | 29 | 2362 | Antisense |

**>HG-U133_PLUS_2:203638_S_AT GENE FGFR2**

caacgtctaactggacttcccaagataaatggtaccagcgtcctcttaaaagatgccttaatccattccttgaggacagaccttagttgaaatgatagcagaatgtgcttctctctggcagctggccttctgcttctgagttgcacattaatcagattagcctgattctcttcagtgaattttgataatggcttccagactctttgcgttggagacgcctgttaggatcttcaagtcccatcatagaaaattgaaacacagagttgttctgctgatagttttggggatacgtccatctttttaagggattgctttcatctaattctggcaggacctcaccaaaagatccagcctcatacctacatcagacaaaatatcgccgttgttccttctgtactaaagtattgtgttttgctttggaaacacccactcact

**Alignment (orangutan on top; human on bottom)**

000000001 caacgtctaactggacttcccaagataaatggtaccagcgtcctcttaaa 000000050

<<<<<<<<< ||||||||||||||||||||||||| |||||||||||||||||||||||| <<<<<<<<<

121051229 caacgtctaactggacttcccaagacaaatggtaccagcgtcctcttaaa 121051180

000000051 agatgccttaatccattccttgaggacagaccttagttgaaatgatagca 000000100

<<<<<<<<< ||||||||||||||||||||||||||||||||| |||||||||||||||| <<<<<<<<<

121051179 agatgccttaatccattccttgaggacagacctcagttgaaatgatagca 121051130

000000101 gaatgtgcttctctctggcagctggccttctgcttctgagttgcacatta 000000150

<<<<<<<<< |||||||||||||||||||||||||||||||||||||||||||||||||| <<<<<<<<<

121051129 gaatgtgcttctctctggcagctggccttctgcttctgagttgcacatta 121051080

000000151 atcagattagcctg.attctcttcagtgaattttgataatggcttccaga 000000199

<<<<<<<<< |||||||||||||| ||||||||||||||||||||||||||||||||||| <<<<<<<<<

121051079 atcagattagcctgtattctcttcagtgaattttgataatggcttccaga 121051030

000000200 ctctttg.cgttggagacgcctgttaggatcttcaagtcccatcatagaa 000000248

<<<<<<<<< ||||||| |||| ||||||||| ||||||||||||||||||||||||||| <<<<<<<<<

121051029 ctctttggcgttagagacgcctcttaggatcttcaagtcccatcatagaa 121050980

000000249 aattgaaacacagagttgttctgctgatagttttggggatacgtccatct 000000298

<<<<<<<<< ||||||||||||||||||||||||||||||||||||||||| |||| | | <<<<<<<<<

121050979 aattgaaacacagagttgttctgctgatagttttggggatatgtccgtat 121050930

000000299 ttttaagggattgctttcatctaattctggcaggacctcaccaaaagatc 000000348

<<<<<<<<< |||||||||||||||||||||||||||||||||||||||||||||||||| <<<<<<<<<

121050929 ttttaagggattgctttcatctaattctggcaggacctcaccaaaagatc 121050880

000000349 cagcctcatacctacatcagacaaaatatcgccgttgttccttctgtact 000000398

<<<<<<<<< |||||||||||||||||||||||||||||||||||||||||||||||||| <<<<<<<<<

121050879 cagcctcatacctacatcagacaaaatatcgccgttgttccttctgtact 121050830

000000399 aaagtattgtgttttgctttggaaacacccactcact 000000435

<<<<<<<<< ||||||||||||||||||||||||||||||||||||| <<<<<<<<<

121050829 aaagtattgtgttttgctttggaaacacccactcact 121050793

| Probe Sequence(5'-3') | Probe X | Probe Y | Probe Interrogation Position | Target Strandedness |
| --- | --- | --- | --- | --- |
| CAACGTCTAACTGGACTTCCCAAGA | 752 | 293 | 3995 | Antisense |
| GATAAATGGTACCAGCGTCCTCTTA | 496 | 681 | 4018 | Antisense |
| AAGATGCCTTAATCCATTCCTTGAG | 468 | 255 | 4044 | Antisense |
| CCTTCTGCTTCTGAGTTGCACATTA | 478 | 451 | 4120 | Antisense |
| GCTTCCAGACTCTTTGCGTTGGAGA | 1103 | 517 | 4185 | Antisense |
| GTTAGGATCTTCAAGTCCCATCATA | 77 | 711 | 4215 | Antisense |
| GGGATTGCTTTCATCTAATTCTGGC | 831 | 805 | 4299 | Antisense |
| AGGACCTCACCAAAAGATCCAGCCT | 659 | 79 | 4324 | Antisense |
| AGCCTCATACCTACATCAGACAAAA | 209 | 147 | 4344 | Antisense |
| CAAAATATCGCCGTTGTTCCTTCTG | 492 | 303 | 4364 | Antisense |
| TTTGCTTTGGAAACACCCACTCACT | 418 | 1095 | 4405 | Antisense |

**>HG-U133_PLUS_2:217901_AT GENE DSG2**

cttgactgaagatattttgctagggaagtgaaactttaaaattttgtagattttaaaaaatattgttgaatggtgtcatgcaaaggatttatatagtgtgctcccactaacnntgtacagatcaggacacatatttttagacatctaagtctgtagcttaaatggaggttactcttccatcatctagaattgtttacttagtaattgttgtttcttttattattatagacttactatcagttttattttgccaagtatgcaacaggnatatcactagtatatgaaaatgtaaatatcacttgtgtactcaaacaaaagttggtcttaagcttccaccttgagcagccttggaaacctaacctgcctcttttagcataatcaca

**Alignment (orangutan on top; human on bottom)**

00000001 cttgact 00000007

>>>>>>>> ||||||| >>>>>>>>

43531870 cttgact 43531876

00000023 gggaagtgaaactttaaaattttgtagattttaaaaaatattgttgaatg 00000072

>>>>>>>> |||||||||||||||||||||||||||| ||||||||||||||||||||| >>>>>>>>

43531877 gggaagtgaaactttaaaattttgtagactttaaaaaatattgttgaatg 43531926

00000073 gtgtcatgcaaaggatttatatagtgtgctcccactaacnntgtacagat 00000122

>>>>>>>> |||| |||||||||||||||||||||||||||||||||| ||||||||| >>>>>>>>

43531927 gtgttatgcaaaggatttatatagtgtgctcccactaactgtgtacagat 43531976

00000123 caggacacatatttttagacatctaagtctgtagcttaaatggaggttac 00000172

>>>>>>>> ||||||||||||||||||||||||||||||||||||||||||||||||| >>>>>>>>

43531977 caggacacatatttttagacatctaagtctgtagcttaaatggaggttat 43532026

00000173 tcttccatcatctagaattgtttacttagtaattgttgtttcttttatta 00000222

>>>>>>>> |||||||||||||||||| ||||||||||||||||||||||||||||||| >>>>>>>>

43532027 tcttccatcatctagaatggtttacttagtaattgttgtttcttttatta 43532076

00000223 ttatagacttactatcagttttattttgccaagtatgcaacaggnatatc 00000272

>>>>>>>> || ||||||||||| ||||||||||||||||||||||||||||| ||||| >>>>>>>>

43532077 ttgtagacttactaacagttttattttgccaagtatgcaacaggaatatc 43532126

00000273 actagtatatgaaaatgtaaatatcacttgtgtactcaaacaaaagttgg 00000322

>>>>>>>> |||||||| |||||||||||||||||| | |||||||||||||||||||| >>>>>>>>

43532127 actagtatgtgaaaatgtaaatatcacctatgtactcaaacaaaagttgg 43532176

00000323 tcttaagcttccaccttgagcagccttggaaacctaacctgcctctttta 00000372

>>>>>>>> ||||||||||||||||||||||||||||||||||||||||||||||| || >>>>>>>>

43532177 tcttaagcttccaccttgagcagccttggaaacctaacctgcctcttgta 43532226

00000373 gcataatcaca 00000383

>>>>>>>> ||||||||||| >>>>>>>>

43532227 gcataatcaca 43532237

| Probe Sequence(5'-3') | Probe X | Probe Y | Probe Interrogation Position | Target Strandedness |
| --- | --- | --- | --- | --- |
| CTTGACTGAAGATATTTTGCTAGGG * | 291 | 357 | 4998 | Antisense |
| ATTGTTGAATGGTGTCATGCAAAGG | 769 | 3 | 5059 | Antisense |
| GGATTTATATAGTGTGCTCCCACTA | 253 | 837 | 5082 | Antisense |
| GTACAGATCAGGACACATATTTTTA | 734 | 735 | 5112 | Antisense |
| GACATCTAAGTCTGTAGCTTAAATG | 185 | 603 | 5137 | Antisense |
| TGGAGGTTACTCTTCCATCATCTAG | 885 | 903 | 5160 | Antisense |
| TACTCTTCCATCATCTAGAATTGTT | 329 | 1057 | 5167 | Antisense |
| GTAAATATCACTTGTGTACTCAAAC | 166 | 737 | 5286 | Antisense |
| AAGTTGGTCTTAAGCTTCCACCTTG | 305 | 257 | 5313 | Antisense |
| GCAGCCTTGGAAACCTAACCTGCCT | 212 | 535 | 5339 | Antisense |
| ACCTGCCTCTTTTAGCATAATCACA | 1040 | 155 | 5356 | Antisense |

*Middle of probe sequence missing from alignment.

**>HG-U133_PLUS_2:219651_AT GENE DPPA4**

gattcacatacaaaaagctgcacatatttaatgtatcctattgtgtaattaatttttaattttttttgtgtacttcctaaacttatagtcctgcgagtctgggaacagatctgtttttcacttatcctgatttaatgacagtttccaacattgttttgttattacaagtaggggatctttttttttgcccgtttaatgaagatactaaaaataatgcactggaaggagtggaagagttggaaaatttgtaaccatcataatacaggtgtaataggtttgggaaagaatcctcaaaaatgttaaagcaagggaggaaagtttgttgagaagcaagatgttcttctctcctgcccgcccccgccgttggttgttggtggtcagaattattgtgtaataaataatagacattttttcttatactatgtgtattgttccttttgtttcctttttaaacttctcccctgctttatttggatgggtcaagtttctgttctgtttccttcctttctattaatttggaaatgtccttggctttacgattctgcttgtagatacttcccct

**Alignment (orangutan on top; human on bottom)**

00000001 gattcacatacaaaaagctgcacatatttaatgtatcctattgtgtaatt 00000050

>>>>>>>> |||| ||||||||||||||| ||||||||||||||||||||||||||||| >>>>>>>>

23517020 gattgacatacaaaaagctgtacatatttaatgtatcctattgtgtaatt 23517069

00000051 aatttttaattttttt 00000066

>>>>>>>> |||||||| ||||||| >>>>>>>>

23517070 aatttttatttttttt 23517085

00000069 tgtacttcctaaacttatagtcctgcgagtctgggaacagatctgttttt 00000118

>>>>>>>> ||||||||||||| |||||||||||||||||||||||||||||||||||| >>>>>>>>

23517087 tgtacttcctaaatttatagtcctgcgagtctgggaacagatctgttttt 23517136

00000119 cacttatcctgatttaatgacagtttccaacattgttttgttattacaag 00000168

>>>>>>>> |||||||||||||||||||||||||||||||||||||||||||||||||| >>>>>>>>

23517137 cacttatcctgatttaatgacagtttccaacattgttttgttattacaag 23517186

00000169 taggggatc 00000177

>>>>>>>> ||||||||| >>>>>>>>

23517187 taggggatc 23517195

00000178 tttttttttgcccgtttaatgaagatactaaaaataatgcactggaagga 00000227

>>>>>>>> ||||||||||||| |||||||||||||||||||||||| ||||||||||| >>>>>>>>

23517211 tttttttttgcccctttaatgaagatactaaaaataattcactggaagga 23517260

00000228 gtggaagagttggaaaatttgtaaccatcataatacaggtgtaataggtt 00000277

>>>>>>>> |||| ||||||||||||||||||||||||||||||||||||||||||||| >>>>>>>>

23517261 gtggcagagttggaaaatttgtaaccatcataatacaggtgtaataggtt 23517310

00000278 tgggaaagaatcctcaaaaatgttaaagcaagggaggaaagtttgttgag 00000327

>>>>>>>> |||||||||||||||| ||||||| ||||||||||||||||||||||||| >>>>>>>>

23517311 tgggaaagaatcctcagaaatgtttaagcaagggaggaaagtttgttgag 23517360

00000328 aagcaagatgttcttctctcctgcccgcccccgccgttggttgttggtgg 00000377

>>>>>>>> |||||||||||||||||||||||||| || |||||||||||||| >>>>>>>>

23517361 aagcaagatgttcttctctcctgccc.......ccattggttgttggtgg 23517403

00000378 tcagaattatt..gtgtaataaataatagacattttttcttatactatgt 00000425

>>>>>>>> |||||||| || |||||||||||||||||||||||| |||||||||||| >>>>>>>>

23517404 tcagaattgttcagtgtaataaataatagacattttt.cttatactatgt 23517452

00000426 gtattgttccttttgtttcctttttaaacttctcccctgctttatttgga 00000475

>>>>>>>> ||||||||| |||||||||||||||||||||||||||| ||||||||||| >>>>>>>>

23517453 gtattgttcgttttgtttcctttttaaacttctcccctactttatttgga 23517502

00000476 tgggtcaagtttctgttctgtttccttcctttctattaatttggaaatgt 00000525

>>>>>>>> |||||||||||||| ||||||||||||||||||||||||||||||||||| >>>>>>>>

23517503 tgggtcaagtttctattctgtttccttcctttctattaatttggaaatgt 23517552

00000526 ccttggctttacgattctgcttgtagatacttccc 00000560

>>>>>>>> |||| |||||| |||||||||||||||||| |||| >>>>>>>>

23517553 cctttgctttatgattctgcttgtagatacctccc 23517587

| Probe Sequence(5'-3') | Probe X | Probe Y | Probe Interrogation Position | Target Strandedness |
| --- | --- | --- | --- | --- |
| GATTCACATACAAAAAGCTGCACAT | 808 | 691 | 2141 | Antisense |
| GTACTTCCTAAACTTATAGTCCTGC | 561 | 733 | 2210 | Antisense |
| ATAGTCCTGCGAGTCTGGGAACAGA | 706 | 27 | 2225 | Antisense |
| TTTTCACTTATCCTGATTTAATGAC | 351 | 1109 | 2255 | Antisense |
| TTTAATGACAGTTTCCAACATTGTT | 214 | 1103 | 2271 | Antisense |
| GCCGTTGGTTGTTGGTGGTCAGAAT | 646 | 483 | 2500 | Antisense |
| AGACATTTTTTCTTATACTATGTGT | 427 | 121 | 2543 | Antisense |
| GGATGGGTCAAGTTTCTGTTCTGTT | 337 | 841 | 2613 | Antisense |
| GGAAATGTCCTTGGCTTTACGATTC | 82 | 859 | 2658 | Antisense |
| TCCTTGGCTTTACGATTCTGCTTGT | 200 | 979 | 2665 | Antisense |
| GATTCTGCTTGTAGATACTTCCCCT * | 1017 | 691 | 2678 | Antisense |

*End of Probe Sequence is not present in side by side analysis.

**>HG-U133_PLUS_2:220028_AT GENE ACVR2B**

ctttggcttggctgttcgatttgagccagggaaacctccaggggacacccacggacaggtaggcacgagacggtacatggctcctgaggtgctcgagggagccatcaacttccagagagatgccttcctgcgcattgacatgtatgccatggggttggtgctgtgggagcttgtgtctcgctgcaaggctgcagacggacccgtggatgagtacatgctgccctttgaggaagagattggccagcacccttcgttggaggagctgcaggaggtggtggtgcacaagaagatgaggcccaccattaaagatcactggttgaaacacccgggcctggcccagctttgtgtgaccatcgaggagtgctgggaccatgatgcagaggctcgcttgtccgcgggctgtgtggaggagcgggtgtccctgattcggaggtcggtcaacggcactacctcggactgtctcgtttccctggtgacctctgtcaccaatgtggacctgccccctaaagagtcaagcat

**Alignment (orangutan on top; human on bottom)**

000000001 ctttggcttggctgttcgatttgagccagggaaacctccaggggacaccc 000000050

<<<<<<<<< |||||||||||||||||||||||||||||||||||||||||||||||||| <<<<<<<<<

108223593 ctttggcttggctgttcgatttgagccagggaaacctccaggggacaccc 108223544

000000051 acggacag 000000058

<<<<<<<<< |||||||| <<<<<<<<<

108223543 acggacag 108223536

000000059 gtaggcacgagacggtacatggctcctgaggtgctcgagggagccatcaa 000000108

<<<<<<<<< ||||||||||||||||||||||||||||||||||| |||||||||||||| <<<<<<<<<

108222805 gtaggcacgagacggtacatggctcctgaggtgcttgagggagccatcaa 108222756

000000109 cttccagagagatgccttcctgcgcattgacatgtatgccatggggttgg 000000158

<<<<<<<<< |||||||||||||||||||||||||||||||||||||||||||||||||| <<<<<<<<<

108222755 cttccagagagatgccttcctgcgcattgacatgtatgccatggggttgg 108222706

000000159 tgctgtgggagcttgtgtctcgctgcaaggctgcagacg 000000197

<<<<<<<<< ||||||||||||||||||||||||||||||||||||||| <<<<<<<<<

108222705 tgctgtgggagcttgtgtctcgctgcaaggctgcagacg 108222667

000000198 gacccgtggatgagtacatgctgccctttgaggaagagattggccagcac 000000247

<<<<<<<<< |||||||||||||||||||||||||||||||||||||||||||||||||| <<<<<<<<<

108222573 gacccgtggatgagtacatgctgccctttgaggaagagattggccagcac 108222524

000000248 ccttcgttggaggagctgcaggaggtggtggtgcacaagaagatgaggcc 000000297

<<<<<<<<< |||||||||||||||||||||||||||||||||||||||||||||||||| <<<<<<<<<

108222523 ccttcgttggaggagctgcaggaggtggtggtgcacaagaagatgaggcc 108222474

000000298 caccattaaagatcactggttgaaacacccg 000000328

<<<<<<<<< ||||||||||||||||||||||||||||||| <<<<<<<<<

108222473 caccattaaagatcactggttgaaacacccg 108222443

000000329 ggcctggcccagctttgtgtgaccatcgaggagtgctgggaccatgatgc 000000378

<<<<<<<<< |||||||||||||||||||||||||||||||||||||||||||||||||| <<<<<<<<<

108221867 ggcctggcccagctttgtgtgaccatcgaggagtgctgggaccatgatgc 108221818

000000379 agaggctcgcttgtccgcgggctgtgtggaggagcgggtgtccctgattc 000000428

<<<<<<<<< |||||||||||||||||||||||||||||||||||||||||||||||||| <<<<<<<<<

108221817 agaggctcgcttgtccgcgggctgtgtggaggagcgggtgtccctgattc 108221768

000000429 ggaggtcggtcaacggcactacctcggactgtctcgtttccctggtgacc 000000478

<<<<<<<<< |||||||||||||||||||||||||||||||||||||||||||||||||| <<<<<<<<<

108221767 ggaggtcggtcaacggcactacctcggactgtctcgtttccctggtgacc 108221718

000000479 tctgtcaccaatgtggacctgccccctaaagagtcaagcat 000000519

<<<<<<<<< ||||||||||||||||||||||||||||||||||||||||| <<<<<<<<<

108221717 tctgtcaccaatgtggacctgccccctaaagagtcaagcat 108221677

| Probe Sequence(5'-3') | Probe X | Probe Y | Probe Interrogation Position | Target Strandedness |
| --- | --- | --- | --- | --- |
| CTTTGGCTTGGCTGTTCGATTTGAG | 726 | 367 | 1033 | Antisense |
| AGGCACGAGACGGTACATGGCTCCT | 226 | 77 | 1093 | Antisense |
| TGCTCGAGGGAGCCATCAACTTCCA | 760 | 963 | 1122 | Antisense |
| TGCCTTCCTGCGCATTGACATGTAT | 1069 | 971 | 1153 | Antisense |
| CTTGTGTCTCGCTGCAAGGCTGCAG | 381 | 357 | 1202 | Antisense |
| TGAGTACATGCTGCCCTTTGAGGAA | 396 | 941 | 1240 | Antisense |
| GATCACTGGTTGAAACACCCGGGCC | 746 | 679 | 1340 | Antisense |
| CCCAGCTTTGTGTGACCATCGAGGA | 335 | 423 | 1368 | Antisense |
| CCATGATGCAGAGGCTCGCTTGTCC | 900 | 455 | 1402 | Antisense |
| TGACCTCTGTCACCAATGTGGACCT | 516 | 945 | 1506 | Antisense |
| ACCTGCCCCCTAAAGAGTCAAGCAT | 986 | 155 | 1527 | Antisense |

**>HG-U133_PLUS_2:206122_AT GENE SOX15**

gaccttcccgctgcggacagggaagaggcaacctggccagcggcggcccgctctgggggccggggtacgcgaccacccaaccgagcagaggctttgggtacagaccccccagctactcgacagcctacctgcctggcagctatggctcttcccactgcaaactggaagccccctcaccgtgctccctccctcagagtgaccctaggctccagggggaactgctgcccacctatacccactacctgccccctggctctcccactccatacaaccctccccttgctggtgcccccatgcccctaacccacctctaaccctcatggacgcagacctcacgggacgggcctcatcctccttttttaatccagcagcatcccctaccccaggctgtcaaccctttctcctgttggactacagttcagaggcagcctgcagtcctcccatgatagccagggagagccgcacaacatacaattata

**Alignment (orangutan on top; human on bottom)**

0000001 gaccttcccgctgcggacagggaagaggcaacctggccagcggcggcccg 0000050

<<<<<<< ||| |||||||||||||||||||||||||||||||||||||||||||||| <<<<<<<

7601780 gactttcccgctgcggacagggaagaggcaacctggccagcggcggcccg 7601731

0000051 ctctgggggccggggtacgcgaccacccaaccgagcagaggctttgggta 0000100

<<<<<<< |||||||||||||||||||||||||||||||||||||||||||||||||| <<<<<<<

7601730 ctctgggggccggggtacgcgaccacccaaccgagcagaggctttgggta 7601681

0000101 cagaccccccagctactcgacagcctacctgcctggcagctatgg 0000145

<<<<<<< ||||||||||| ||||||||||||||||||||| ||||||||||| <<<<<<<

7601680 cagaccccccaactactcgacagcctacctgcccggcagctatgg 7601636

0000146 ctcttcccactgcaaactggaagccccctcaccgtgctccctccctcaga 0000195

<<<<<<< |||||||||||||||||||||||||||||||||||||||||||||||||| <<<<<<<

7601039 ctcttcccactgcaaactggaagccccctcaccgtgctccctccctcaga 7600990

0000196 gtgaccctaggctccagggggaactgctgcccacctatacccactacctg 0000245

<<<<<<< |||||||||||||||||||||| ||||||||||||||||||||||||||| <<<<<<<

7600989 gtgaccctaggctccagggggagctgctgcccacctatacccactacctg 7600940

0000246 ccccctggctctcccactccatacaaccctccccttgctggtgcccccat 0000295

<<<<<<< |||||||||||||||||||||||||||||||||||| ||||||||||||| <<<<<<<

7600939 ccccctggctctcccactccatacaaccctcccctttctggtgcccccat 7600890

0000296 gcccctaacccacctctaaccctcatggacgcagacctcacgggacgggc 0000345

<<<<<<< |||||||||||||||||||||||||||||||||||||||| |||||||| <<<<<<<

7600889 gcccctaacccacctctaaccctcatggacgcagacctcagcggacgggc 7600840

0000346 ctcatcctccttttttaatccagcagcatcccctaccccaggctgtcaac 0000395

<<<<<<< |||||||| |||||| ||| ||||||||||||||||||||||||||||| <<<<<<<

7600839 ctcatcct..ttttttcatctagcagcatcccctaccccaggctgtcaac 7600792

0000396 cctttctcctgttggactacagttcagaggcagcctgcagtcctcccatg 0000445

<<<<<<< |||||||||||||||||||||||||||||||||||||||||||||||||| <<<<<<<

7600791 cctttctcctgttggactacagttcagaggcagcctgcagtcctcccatg 7600742

0000446 atagccagggagagccgcacaacatacaattata 0000479

<<<<<<< |||||||||||||||||||||||||||| ||||| <<<<<<<

7600741 atagccagggagagccgcacaacatacagttata 7600708

| Probe Sequence(5'-3') | Probe X | Probe Y | Probe Interrogation Position | Target Strandedness |
| --- | --- | --- | --- | --- |
| GACCTTCCCGCTGCGGACAGGGAAG | 1102 | 619 | 895 | Antisense |
| CCACCCAACCGAGCAGAGGCTTTGG | 921 | 461 | 967 | Antisense |
| GAGCAGAGGCTTTGGGTACAGACCC | 712 | 631 | 977 | Antisense |
| GACCCTAGGCTCCAGGGGGAACTGC | 794 | 619 | 1092 | Antisense |
| TCCTCCTTTTTTAATCCAGCAGCAT | 653 | 983 | 1244 | Antisense |
| ACCCTTTCTCCTGTTGGACTACAGT | 325 | 159 | 1288 | Antisense |
| GGACTACAGTTCAGAGGCAGCCTGC | 388 | 855 | 1303 | Antisense |
| CAGTTCAGAGGCAGCCTGCAGTCCT | 467 | 333 | 1309 | Antisense |
| CAGTCCTCCCATGATAGCCAGGGAG | 423 | 335 | 1327 | Antisense |
| TGATAGCCAGGGAGAGCCGCACAAC | 512 | 935 | 1338 | Antisense |
| GAGAGCCGCACAACATACAATTATA | 1055 | 639 | 1349 | Antisense |

**>HG-U133_PLUS_2:206309_AT GENE LECT1**

aggactcacaagccttcaatcctgataatccttatcatcagcaggaaggggaaagcatgacattcgaccctagactggatcacgaaggaatctgttgtatagaatgtaggcggagctacacccactgccagaagatctgtgaacccctggggggctattacccatggccttataattatcaaggctgccgttcggcctgcagagtcatcatgccatgtagctggtgggtggcccgtatcttgggcatggtgtgaaatcacttcatatatcacgtgctgtaaaataagaactagctgaagagacaaccaaagaagcattaaggcaggttgatgctgatgggaccataaaatatttttacacgcagcctgagcggttattcttgacactcttaacagaatttttttaatcgttttccagaactttagtatatgcaaatgcactgaaagggtagttcaagtctaaaatgccataaccccgttatttgttattttttatttgcattgatttgccataagtcttcccttgcttgcatcttccaaagcta

**Alignment (orangutan on top; human on bottom)**

00000001 aggactcacaagccttcaatcctgataatccttatcat 00000038

<<<<<<<< ||||||| |||||||||||||||||||||||||||||| <<<<<<<<

53011344 aggactcccaagccttcaatcctgataatccttatcat 53011307

00000039 cagcaggaaggggaaagcatgacattcgaccctagactggatcacgaagg 00000088

<<<<<<<< |||||||||||||||||||||||||||||||||||||||||||||||||| <<<<<<<<

53006309 cagcaggaaggggaaagcatgacattcgaccctagactggatcacgaagg 53006260

00000089 aatctgttgtatagaatgtaggcggagctacacccactgccagaagatct 00000138

<<<<<<<< |||||||||||||||||||||||||||||||||||||||||||||||||| <<<<<<<<

53006259 aatctgttgtatagaatgtaggcggagctacacccactgccagaagatct 53006210

00000139 gtgaacccctggggggctattacccatggccttataattatcaaggctgc 00000188

<<<<<<<< |||||||||||||||||||||||||||||||||||||||||||||||||| <<<<<<<<

53006209 gtgaacccctggggggctattacccatggccttataattatcaaggctgc 53006160

00000189 cgttcggcctgcagagtcatcatgccatgtagctggtgggtggcccgtat 00000238

<<<<<<<< |||||||||||||||||||||||||||||||||||||||||||||||||| <<<<<<<<

53006159 cgttcggcctgcagagtcatcatgccatgtagctggtgggtggcccgtat 53006110

00000239 cttgggcatggtgtgaaatcacttcatatatcacgtgctgtaaaataaga 00000288

<<<<<<<< ||||||||||||||||||||||||||| ||||| ||||||||||||||| <<<<<<<<

53006109 cttgggcatggtgtgaaatcacttcatgtatcatctgctgtaaaataaga 53006060

00000289 actagctgaagagacaaccaaagaagcattaaggcaggttgatgctgatg 00000338

<<<<<<<< |||||||||||||| ||||||||||||||||||||||||||||||||||| <<<<<<<<

53006059 actagctgaagaga.aaccaaagaagcattaaggcaggttgatgctgatg 53006011

00000339 ggaccataaaatatttttacacgcagcctgagcggttattcttgacactc 00000388

<<<<<<<< |||||| ||||||||||||||| || |||||||||||||||||||||||| <<<<<<<<

53006010 ggaccacaaaatatttttacactcaacctgagcggttattcttgacactc 53005961

00000389 ttaacagaattttttt.aatcgttttccagaactttagtatatgcaaatg 00000437

<<<<<<<< |||||||||||||||| ||| ||||||||||||||||||||||||||||| <<<<<<<<

53005960 ttaacagaatttttttcaattgttttccagaactttagtatatgcaaatg 53005911

00000438 cactgaaagggtagttcaagtctaaaatgccataacccc 00000476

<<<<<<<< |||||||||||||||||||||||||||||||||||||| <<<<<<<<

53005910 tactgaaagggtagttcaagtctaaaatgccataacccc 53005872

00000478 ttatttgttattttttatttgcattgatttgccataagtcttcccttgct 00000527

<<<<<<<< |||||||||||||||||||||||||| ||||||||||||||||||||||| <<<<<<<<

53005867 ttatttgttattttttatttgcattgctttgccataagtcttcccttgct 53005818

00000528 tgcatcttccaaagcta 00000544

<<<<<<<< || |||||||||| ||| <<<<<<<<

53005817 tgtatcttccaaaccta 53005801

| Probe Sequence(5'-3') | Probe X | Probe Y | Probe Interrogation Position | Target Strandedness |
| --- | --- | --- | --- | --- |
| AGGACTCACAAGCCTTCAATCCTGA | 321 | 77 | 764 | Antisense |
| GACATTCGACCCTAGACTGGATCAC | 1035 | 607 | 822 | Antisense |
| AATGTAGGCGGAGCTACACCCACTG | 988 | 269 | 866 | Antisense |
| GATCTGTGAACCCCTGGGGGGCTAT | 180 | 671 | 897 | Antisense |
| AATTATCAAGGCTGCCGTTCGGCCT | 468 | 285 | 937 | Antisense |
| CATGTAGCTGGTGGGTGGCCCGTAT | 141 | 341 | 977 | Antisense |
| GTGGCCCGTATCTTGGGCATGGTGT | 401 | 791 | 991 | Antisense |
| AAATATTTTTACACGCAGCCTGAGC | 803 | 199 | 1110 | Antisense |
| CAGCCTGAGCGGTTATTCTTGACAC | 87 | 323 | 1125 | Antisense |
| ATTGATTTGCCATAAGTCTTCCCTT | 1127 | 3 | 1263 | Antisense |
| CCCTTGCTTGCATCTTCCAAAGCTA | 380 | 425 | 1283 | Antisense |

**>HG-U133_PLUS_2:204269_AT GENE PIM2**

Aacttagttcatatgcttttacttgggcaagggtgctttccttccaataccccagtagcttttattttagtaaagggaccctttcccctagcctagggtcccatattgggtcaagctgcttacctgcctcagcccaggattttttattttgggggaggtaatgccctgttgttaccccaaggcttctttttttttttttttttttttgggtgaggggaccctactttgttatcccaagtgctcttattctggtgagaagaaccttaattccataatttgggaaggaatggaagatggacaccaccggacaccaccagacaataggatgggatggatggttttttgggggatgggctaggggaaataaggcttgctgtttgttttcctggggcgctccctccaattttgcagatttttgcaacctcctcctgagccgggattgtccaattactaaaatgtaaataatcacgtattgtggggaggggagttccaagtgtgccctccttttttttcctgcctggattatttaaaaagccatgtgtggaaacccactatt

**Alignment (orangutan on top; human on bottom)**

00000001 aacttagttcatatgcttttacttgggcaagggtgctttccttcc...aa 00000047

<<<<<<<< ||||||||||||||||| ||||||||||||||||||||||||||| || <<<<<<<<

49698880 aacttagttcatatgctcttacttgggcaagggtgctttccttccaataa 49698831

00000048 taccccagtagcttttattttagtaaagggaccctttcccctagcctagg 00000097

<<<<<<<< |||||||||||||||||||||||||||||||||||||||||||||||||| <<<<<<<<

49698830 taccccagtagcttttattttagtaaagggaccctttcccctagcctagg 49698781

00000098 gtcccatattgggtcaagctgcttacctgcctcagcccaggattttttat 00000147

<<<<<<<< |||||||||||||||||||||||||||||||||||||||||||| ||||| <<<<<<<<

49698780 gtcccatattgggtcaagctgcttacctgcctcagcccaggattctttat 49698731

00000148 tttgggggaggtaatgccctgttgttaccccaaggctt 00000185

<<<<<<<< | |||||||||||||||||||||||||||||||||||| <<<<<<<<

49698730 tctgggggaggtaatgccctgttgttaccccaaggctt 49698693

00000187 tttttttttttttttttttttgggtgaggggaccctactttgttatccca 00000236

<<<<<<<< ||||||||||||||||||||||||||||||||||||||| |||||||||| <<<<<<<<

49698685 tttttttttttttttttttttgggtgaggggaccctactctgttatccca 49698636

00000237 agtgctcttattctggtgagaagaaccttaattccataatttgggaagga 00000286

<<<<<<<< |||||||||||||||||||||||||||||| ||||||||||||||||||| <<<<<<<<

49698635 agtgctcttattctggtgagaagaaccttacttccataatttgggaagga 49698586

00000287 atggaagatggacaccaccggacaccaccagacaataggatgggatggat 00000336

<<<<<<<< |||||||||||||||||||||||||||||||||| ||||||||||||||| <<<<<<<<

49698585 atggaagatggacaccaccggacaccaccagacactaggatgggatggat 49698536

00000337 ggttttttgggggatgggctaggggaaataaggcttgctgtttgttttcc 00000386

<<<<<<<< |||||||||||||||||||||||||||||||||||||||||||||| ||| <<<<<<<<

49698535 ggttttttgggggatgggctaggggaaataaggcttgctgtttgttctcc 49698486

00000387 tggggcgctccctccaa.ttttgcagatttttgcaacctcctcctgagcc 00000435

<<<<<<<< |||||||| |||||||| ||||||||||| |||||||||||||||||||| <<<<<<<<

49698485 tggggcgccccctccaacttttgcagattcttgcaacctcctcctgagcc 49698436

00000436 gggattgtccaattactaaaatgtaaataatcacgtattgtggggagggg 00000485

<<<<<<<< |||||||||||||| ||||||||||||||||||||||||||||||||||| <<<<<<<<

49698435 gggattgtccaattgctaaaatgtaaataatcacgtattgtggggagggg 49698386

00000486 agttccaagtgtgccctcct 00000505

<<<<<<<< |||||||||||||||||||| <<<<<<<<

49698385 agttccaagtgtgccctcct 49698366

00000512 tcctgcctggattatttaaaaagccatgtgtggaaacccactatt 00000556

<<<<<<<< ||||||||||||||||||||||||||||||||||||||||||||| <<<<<<<<

49698356 tcctgcctggattatttaaaaagccatgtgtggaaacccactatt 49698312

| Probe Sequence(5'-3') | Probe X | Probe Y | Probe Interrogation Position | Target Strandedness |
| --- | --- | --- | --- | --- |
| AACTTAGTTCATATGCTTTTACTTG | 14 | 235 | 1505 | Antisense |
| TTACTTGGGCAAGGGTGCTTTCCTT | 649 | 1121 | 1523 | Antisense |
| CCCTAGCCTAGGGTCCCATATTGGG | 888 | 425 | 1590 | Antisense |
| TATTGGGTCAAGCTGCTTACCTGCC | 914 | 1089 | 1608 | Antisense |
| TGGGGGAGGTAATGCCCTGTTGTTA | 760 | 909 | 1654 | Antisense |
| GACCCTACTTTGTTATCCCAAGTGC | 515 | 619 | 1721 | Antisense |
| TATCCCAAGTGCTCTTATTCTGGTG | 88 | 1081 | 1734 | Antisense |
| GGTGAGAAGAACCTTAATTCCATAA | 699 | 819 | 1755 | Antisense |
| ACCGGACACCACCAGACAATAGGAT | 730 | 157 | 1807 | Antisense |
| GAGGGGAGTTCCAAGTGTGCCCTCC | 358 | 653 | 1984 | Antisense |
| AAGCCATGTGTGGAAACCCACTATT | 793 | 245 | 2036 | Antisense |

**>HG-U133_PLUS_2:205268_S_AT GENE ADD2**

Taagaggccaggaacttgacccctttgaattgtgcatctcaggcacttcaaaactaaaaccaaatttagcataggaaaaagttgtttaatgctcagggcagaaatttgggaagttgaaatcctctgttggctttgggttgtataaggaggatcaaaacaacagaggaaatgctgactttctagctttgcatgacacctggagcaatgcactgtacctgcctcactcctgtccagtggtcaggtttcccccgaccttccctcacccccagaaacacttgcttacagaccgaaactggcatcttactcttggcaccttgacttgcaccctctgaggttccaactcagtcattctttgtccagcagaggagaatcagaaatgagcccttcaggattaatcctcttgcaccagctctcagagaaatgctgggtatccctgtccgtccctatctgtccatcctggggcctggtaatggccacagttattgttttaaatgccaacactgtcttctcatgttcttccgtggggcattgat

**Alignment (orangutan on top; human on bottom)**

00000001 taagaggccaggaacttgacccctttgaattgtgcatctcaggcacttca 00000050

>>>>>>>> |||||||||||||||||||||||||||||||||||||||||||||||||| >>>>>>>>

40133054 taagaggccaggaacttgacccctttgaattgtgcatctcaggcacttca 40133103

00000051 aaactaaaaccaaatttagcataggaaaaagttgtttaatgctcagggca 00000100

>>>>>>>> |||||||||||||||||||||||||||||||||||||||||||||||||| >>>>>>>>

40133104 aaactaaaaccaaatttagcataggaaaaagttgtttaatgctcagggca 40133153

00000101 gaaatttggg.aagttgaaatcctctgttggctttgggttgtataaggag 00000149

>>>>>>>> |||||||||| ||||||||||||||| ||||||||||||||||||||||| >>>>>>>>

40133154 gaaatttggggaagttgaaatcctctcttggctttgggttgtataaggag 40133203

00000150 gatcaaaacaacagaggaaatgc..tgactttctagctttgcatgacacc 00000197

>>>>>>>> |||||||||||||||| ||||| ||||||||||||||||||||||||| >>>>>>>>

40133204 gatcaaaacaacagagagaatgctatgactttctagctttgcatgacacc 40133253

00000198 tggagcaatgcactgtacctgcctcactcctgtccagtggtcaggtttcc 00000247

>>>>>>>> |||||||||||||||||||||||||||||||||||||| ||||||| ||| >>>>>>>>

40133254 tggagcaatgcactgtacctgcctcactcctgtccagtagtcaggtgtcc 40133303

00000248 cccgaccttccctcacccccagaaacacttgcttacagaccgaaactggc 00000297

>>>>>>>> || ||||||||||||||||||||||||||||||||||||||||||||||| >>>>>>>>

40133304 cctgaccttccctcacccccagaaacacttgcttacagaccgaaactggc 40133353

00000298 atcttactcttggcaccttgacttgcaccctctgaggttccaactc..ag 00000345

>>>>>>>> ||||||||||||||||||||| ||| |||||||||||||||||||| || >>>>>>>>

40133354 atcttactcttggcaccttgatttgtaccctctgaggttccaactctgag 40133403

00000346 tcattctttgtccagcagaggagaatcagaaatgagcccttcaggattaa 00000395

>>>>>>>> ||| |||| ||||||||||||||||||||||||||||||||||||||||| >>>>>>>>

40133404 tcactcttggtccagcagaggagaatcagaaatgagcccttcaggattaa 40133453

00000396 tcctcttgcaccagctctcagagaaatgctgggtatccctgtcc 00000439

>>>>>>>> |||||||||||||||||||||||||||||||||| | ||||||| >>>>>>>>

40133454 tcctcttgcaccagctctcagagaaatgctgggtgttcctgtcc 40133497

00000441 tccctatctgtccatcctggggcctggtaatggccacagttattgtttta 00000490

>>>>>>>> |||||||||||||||||||||||||||||||||||||||||||||||||| >>>>>>>>

40133501 tccctatctgtccatcctggggcctggtaatggccacagttattgtttta 40133550

00000491 aatgccaacactgtcttctcatgttcttccgtggggcattgat 00000533

>>>>>>>> |||||||||||||||||||| |||||||| ||||||||||||| >>>>>>>>

40133551 aatgccaacactgtcttctcgtgttcttctgtggggcattgat 40133593

| Probe Sequence(5'-3') | Probe X | Probe Y | Probe Interrogation Position | Target Strandedness |
| --- | --- | --- | --- | --- |
| TAAGAGGCCAGGAACTTGACCCCTT | 266 | 1037 | 3453 | Antisense |
| ACTTGACCCCTTTGAATTGTGCATC | 1152 | 177 | 3466 | Antisense |
| TTGAAATCCTCTGTTGGCTTTGGGT | 1148 | 1149 | 3566 | Antisense |
| GACTTTCTAGCTTTGCATGACACCT | 1095 | 609 | 3626 | Antisense |
| CCTGGAGCAATGCACTGTACCTGCC | 471 | 439 | 3648 | Antisense |
| TCCTGTCCAGTGGTCAGGTTTCCCC | 231 | 985 | 3677 | Antisense |
| GAAACACTTGCTTACAGACCGAAAC | 540 | 587 | 3721 | Antisense |
| ACCGAAACTGGCATCTTACTCTTGG | 256 | 157 | 3738 | Antisense |
| CCAACTCAGTCATTCTTTGTCCAGC | 786 | 465 | 3789 | Antisense |
| TAAATGCCAACACTGTCTTCTCATG | 220 | 1049 | 3941 | Antisense |
| TCATGTTCTTCCGTGGGGCATTGAT | 682 | 1019 | 3961 | Antisense |

**>HG-U133_PLUS_2:205350_AT GENE CRABP1**

aggcactgggtgtgaacgccatgctgaggaaagtggccgtagcggctgcgtccaagccgcacgtggagatccgccaggacggggatcagttctacatcaagacatccaccaccgtgcgcaccactgagatcaacttcaaggtcggagaaggctttgaggaggagaccgtggacggacgcaagtgcaggagtttagccacttgggagaatgagaacaagatccactgcacccaaactcttcttgaaggggacggccccaaaacctactggacccgtgagctggccaacgatgaacttatcctgacgtttggcgccgatgacgtggtctgcaccagaatttatgtccgggaatgaaggcagctggcttgctcctactttcaggaagggatgcaggtccccgaggaatatgtcatagttctgagctgccagtggaccgcccttttcccctaccaatattaggtgatcccgttttccccatgacaatgttgtagtgtcccccacccccacccccctggccttggtgcctcttgtatccctagtgctgcatagccc

**Alignment (orangutan on top; human on bottom)**

00000009 ggtgtgaacgccatgctgaggaaagtggccgtagcggctgcgtccaagcc 00000058

>>>>>>>> |||||||||||||||||||||||||||||||||||||||||||||||||| >>>>>>>>

75941427 ggtgtgaacgccatgctgaggaaagtggccgtagcggctgcgtccaagcc 75941476

00000059 gcacgtggagatccgccaggacggggatcagttctacatcaagacatcca 00000108

>>>>>>>> |||||||||||||||||||||||||||||||||||||||||||||||||| >>>>>>>>

75941477 gcacgtggagatccgccaggacggggatcagttctacatcaagacatcca 75941526

00000109 ccaccgtgcgcaccactgagatcaacttcaaggtcggagaaggctttgag 00000158

>>>>>>>> |||| ||||||||||||||||||||||||||||||||||||||||||||| >>>>>>>>

75941527 ccacggtgcgcaccactgagatcaacttcaaggtcggagaaggctttgag 75941576

00000159 gaggagaccgtggacggacgcaagtgcagg 00000188

>>>>>>>> |||||||||||||||||||||||||||||| >>>>>>>>

75941577 gaggagaccgtggacggacgcaagtgcagg 75941606

00000189 agtttagccacttgggagaatgagaacaagatccactgcacccaaactct 00000238

>>>>>>>> ||||||||||||||||||||||||||||||||||||||||| |||||||| >>>>>>>>

75943893 agtttagccacttgggagaatgagaacaagatccactgcacgcaaactct 75943942

00000239 tcttgaaggggacggccccaaaacctactggacccgtgagctggccaacg 00000288

>>>>>>>> |||||||||||||||||||||||||||||||||||||||||||||||||| >>>>>>>>

75943943 tcttgaaggggacggccccaaaacctactggacccgtgagctggccaacg 75943992

00000289 atgaacttatcctg 00000302

>>>>>>>> |||||||||||||| >>>>>>>>

75943993 atgaacttatcctg 75944006

00000303 acg 00000305

>>>>>>>> ||| >>>>>>>>

75944346 acg 75944348

00000306 tttggcgccgatgacgtggtctgcaccagaatttatgtccgggaatgaag 00000355

>>>>>>>> ||||||||||||||||||||||||||||||||||||||||| || ||||| >>>>>>>>

75948334 tttggcgccgatgacgtggtctgcaccagaatttatgtccgagagtgaag 75948383

00000356 gcagctggcttgctcctactttcaggaagggatgcagg.tcccc.gagga 00000403

>>>>>>>> |||||||||||||||||||||||||||||||||||||| ||||| ||||| >>>>>>>>

75948384 gcagctggcttgctcctactttcaggaagggatgcaggctcccctgagga 75948433

00000404 atatgtcatagttctgagctgccagtggaccgcccttttcccctaccaat 00000453

>>>>>>>> ||||||||||||||||||||||| |||||||||||||||||||||||||| >>>>>>>>

75948434 atatgtcatagttctgagctgccggtggaccgcccttttcccctaccaat 75948483

00000454 attaggtgatcccgttttccccatgacaatgttgtagtgtcccccacccc 00000503

>>>>>>>> |||||||||||| ||||| |||||||| |||||||||||||||| >>>>>>>>

75948484 attaggtgatcctgtttt.cccatgactatgttgtagtgtcccc...... 75948526

00000504 cacccccc 00000511

>>>>>>>> ||||||| >>>>>>>>

75948527 .acccccc 75948533

00000515 ccttggtgcctcttgtatccctagtgctgcatag 00000548

>>>>>>>> |||||||||||||||||||||||||||| ||||| >>>>>>>>

75948544 ccttggtgcctcttgtatccctagtgctccatag 75948577

| Probe Sequence(5'-3') | Probe X | Probe Y | Probe Interrogation Position | Target Strandedness |
| --- | --- | --- | --- | --- |
| AGGCACTGGGTGTGAACGCCATGCT* | 898 | 75 | 148 | Antisense |
| GGACGGGGATCAGTTCTACATCAAG | 769 | 855 | 224 | Antisense |
| GACCGTGGACGGACGCAAGTGCAGG | 72 | 621 | 311 | Antisense |
| CCAAAACCTACTGGACCCGTGAGCT | 156 | 465 | 403 | Antisense |
| TGAGCTGGCCAACGATGAACTTATC | 774 | 943 | 422 | Antisense |
| ATGAACTTATCCTGACGTTTGGCGC | 595 | 53 | 436 | Antisense |
| TCTGCACCAGAATTTATGTCCGGGA | 196 | 1001 | 472 | Antisense |
| TGGCTTGCTCCTACTTTCAGGAAGG | 589 | 897 | 508 | Antisense |
| GATGCAGGTCCCCGAGGAATATGTC | 1005 | 671 | 533 | Antisense |
| CATAGTTCTGAGCTGCCAGTGGACC | 117 | 351 | 557 | Antisense |
| TTGTATCCCTAGTGCTGCATAGCCC** | 237 | 1155 | 674 | Antisense |

*Beginning of Probe Sequence is not present in side by side alignment.

**End of Probe Sequence is not present in side by side alignment.

**>HG-U133_PLUS_2:214240_AT GENE GAL**

Tctggtcgccggccaaggaaaaacgaggctggaccctgaacagcgcgggctacctgctgggcccacatgccgttggcaaccacaggtcattcagcgacaagaatggcctcaccagcaagcgggagctgcggcccgaagatgacatgaaaccaggaagctttgacaggtccatacctgaaaacaatatcatgcgcacaatcattgagtttctgtctttcttgcatctcaaagaggccggtgccc

**Alignment (orangutan on top; human on bottom)**

0000011 ggccaaggaaaaacgaggctggaccctgaacagcgcgggctacctgctgg 0000060

<<<<<<< |||||||||||||||||||||||||||||||||||||||||||||||||| <<<<<<<

7398562 ggccaaggaaaaacgaggctggaccctgaacagcgcgggctacctgctgg 7398513

0000061 gcccac 0000066

<<<<<<< |||||| <<<<<<<

7398512 gcccac 7398507

0000067 atgccgttggcaaccacaggtcattcagcgacaagaatggcctcaccagc 0000116

<<<<<<< ||||||||||||||||||||||||||| ||||||||||||||||| ||| <<<<<<<

7396164 atgccgttggcaaccacaggtcattcaatgacaagaatggcctcactagc 7396115

0000117 aagcgggagctgcggcccgaagatgacatgaaaccag 0000153

<<<<<<< ||||||||||||| ||||||||||||||||||||||| <<<<<<<

7396114 aagcgggagctgcagcccgaagatgacatgaaaccag 7396078

0000154 gaagctttgacaggtccatacctgaaaacaatatcatgcgcacaatcatt 0000203

<<<<<<< ||||||||||||||||||||||||| |||||||||||||||||||||||| <<<<<<<

7395323 gaagctttgacaggtccatacctgagaacaatatcatgcgcacaatcatt 7395274

0000204 gagtttctgtctttcttgcatctcaaag 0000231

<<<<<<< |||||||||||||||||||||||||||| <<<<<<<

7395273 gagtttctgtctttcttgcatctcaaag 7395246

| Probe Sequence(5'-3') | Probe X | Probe Y | Probe Interrogation Position | Target Strandedness |
| --- | --- | --- | --- | --- |
| TCTGGTCGCCGGCCAAGGAAAAACG * | 477 | 997 | 280 | Antisense |
| GAAAAACGAGGCTGGACCCTGAACA | 232 | 585 | 297 | Antisense |
| CCACATGCCGTTGGCAACCACAGGT | 586 | 465 | 342 | Antisense |
| GGCAACCACAGGTCATTCAGCGACA | 375 | 871 | 354 | Antisense |
| CAGCGACAAGAATGGCCTCACCAGC | 984 | 323 | 371 | Antisense |
| GCCTCACCAGCAAGCGGGAGCTGCG | 396 | 479 | 385 | Antisense |
| GGAGCTGCGGCCCGAAGATGACATG | 745 | 851 | 401 | Antisense |
| AGGAAGCTTTGACAGGTCCATACCT | 488 | 77 | 431 | Antisense |
| ATATCATGCGCACAATCATTGAGTT | 750 | 23 | 463 | Antisense |
| TGAGTTTCTGTCTTTCTTGCATCTC | 514 | 941 | 482 | Antisense |
| TTGCATCTCAAAGAGGCCGGTGCCC ** | 269 | 1147 | 498 | Antisense |

*Beginning of probe sequence not present in side by side alignment.

**End of probe sequence not present in side by side alignment.

**>HG-U133_PLUS_2:204469_AT GENE PTPRZ1**

gtaaataaaacactcttccatatgatattcaacattttacaactgcagtattcacctaaagtagaaataatctgttacttattgtaaatactgccctagtgtctccatggaccaaatttatatttataattgtagatttttatattttactactgagtcaagttttctagttctgtgtaattgtttagtttaatgacgtagttcattagctggtcttactctaccagttttctgacattgtattgtgttacctaagtcattaactttgtttcagcatgtaattttaacttttgtggaaaatagaaataccttcattttgaaagaagtttttatgagaataacaccttaccaaacattg

**Alignment (orangutan on top; human on bottom)**

000000001 gtaaataaaacactcttccatatgatattcaacattttacaactgcagta 000000050

>>>>>>>>> ||||||||||||||||| | |||||||||||||||||||||||||||||| >>>>>>>>>

118927465 gtaaataaaacactcttgcctatgatattcaacattttacaactgcagta 118927514

000000051 ttcacctaaagtagaaataatctgttacttattgtaaatactgccctagt 000000100

>>>>>>>>> |||||||||||||||||||||||||||||||||||||||||||||||||| >>>>>>>>>

118927515 ttcacctaaagtagaaataatctgttacttattgtaaatactgccctagt 118927564

000000101 gtctccatggaccaaatttatatttataattgtagatttttatattttac 000000150

>>>>>>>>> |||||||||||||||||||||||||||||||||||||||||||||||||| >>>>>>>>>

118927565 gtctccatggaccaaatttatatttataattgtagatttttatattttac 118927614

000000151 tactgagtcaagttttctagttctgtgtaattgtttagtttaatgacgta 000000200

>>>>>>>>> |||||||||||||||||||||||||||||||||||||||||||||||||| >>>>>>>>>

118927615 tactgagtcaagttttctagttctgtgtaattgtttagtttaatgacgta 118927664

000000201 gttcattagctggtcttactctaccagttttctgacattgtattgtgtta 000000250

>>>>>>>>> |||||||| |||||||||||||||||||||||||||| |||||| ||||| >>>>>>>>>

118927665 gttcattatctggtcttactctaccagttttctgacactgtattatgtta 118927714

000000251 cctaagtcattaactttgtttcagcatgtaattttaacttttgtggaaaa 000000300

>>>>>>>>> |||||||||||||||||||||||||||||||||||||||| |||||||| >>>>>>>>>

118927715 tctaagtcattaactttgtttcagcatgtaattttaactttcgtggaaaa 118927764

000000301 tagaaataccttcattttgaaagaagtttttatgagaataacaccttacc 000000350

>>>>>>>>> |||||||||||| |||||||||||||||||||||||||||||||||||| >>>>>>>>>

118927765 tagaaatacctttgttttgaaagaagtttttatgagaataacaccttacc 118927814

000000351 aaacattg 000000358

>>>>>>>>> |||||||| >>>>>>>>>

118927815 aaacattg 118927822

| Probe Sequence(5'-3') | Probe X | Probe Y | Probe Interrogation Position | Target Strandedness |
| --- | --- | --- | --- | --- |
| GTAAATAAAACACTCTTCCATATGA | 123 | 737 | 7508 | Antisense |
| ATATTCAACATTTTACAACTGCAGT | 162 | 21 | 7532 | Antisense |
| ACAACTGCAGTATTCACCTAAAGTA | 252 | 195 | 7546 | Antisense |
| TGTAAATACTGCCCTAGTGTCTCCA | 75 | 925 | 7590 | Antisense |
| CCCTAGTGTCTCCATGGACCAAATT | 958 | 425 | 7601 | Antisense |
| GAGTCAAGTTTTCTAGTTCTGTGTA | 129 | 649 | 7662 | Antisense |
| GACGTAGTTCATTAGCTGGTCTTAC | 479 | 617 | 7702 | Antisense |
| GGTCTTACTCTACCAGTTTTCTGAC | 983 | 823 | 7719 | Antisense |
| ACCAGTTTTCTGACATTGTATTGTG | 641 | 151 | 7730 | Antisense |
| ATTGTATTGTGTTACCTAAGTCATT | 174 | 5 | 7744 | Antisense |
| GAGAATAACACCTTACCAAACATTG | 784 | 633 | 7841 | Antisense |

**>HG-U133_PLUS_2:215028_AT GENE SEMA6A**

cactgaattttctcttaggctgaccatatattttattaaagtagtcttgaattcaggagtggaaaaaaaaaagttatgttttagacctcaatgtaagctgaaaggaaataaaggaacttctatatttccatttctcaggctgattttccttcccctgttctcttatctgtatcatgacatgagangtgaacctcagaaatgtaaaaacaaaaagtcntagaataaagcattccnttgaattttaaatgaacatgcttttagcagcntggatacgaagcagactattttcaacaaattgaatgtttcccnacttgtgtgagagccaaacaagtgccatacagaaagggacctcttcaatgttaccnaaagctttgtggcagcatttaagtgtggtggctccatagaagtcactatagcatggacgagccacttggcttgct

#### Alignment (orangutan on top; human on bottom)

000000001 cactgaattttctcttaggctgaccatatattttattaaagtagtcttga 000000050

<<<<<<<<< |||||||||||||||||||||||||||||||||| ||| ||||||||||| <<<<<<<<<

117584064 cactgaattttctcttaggctgaccatatattttgttagagtagtcttga 117584015

000000051 attcaggagtggaaaaaaaaaagttatgttttagacctcaatgtaagctg 000000100

<<<<<<<<< |||||||||||||||||||||||||||||||||||||||||||||||||| <<<<<<<<<

117584014 attcaggagtggaaaaaaaaaagttatgttttagacctcaatgtaagctg 117583965

000000101 aaaggaaataaaggaacttctatatttccatttctcaggctgattttcct 000000150

<<<<<<<<< ||||||| |||||||||||||||||||||||||||||||||||||||||| <<<<<<<<<

117583964 aaaggaagtaaaggaacttctatatttccatttctcaggctgattttcct 117583915

000000151 tcccctgttctcttatctgtatcatgacatgagangtgaacctcagaaat 000000200

<<<<<<<<< ||||||||||||||||||||||||||||||||| ||||| ||||||||| <<<<<<<<<

117583914 gcccctgttctcttatctgtatcatgacatgagatgtgaaactcagaaat 117583865

000000201 gtaaaaacaaaaagtcntagaataaagcattccnttgaattttaaatgaa 000000250

<<<<<<<<< |||||||||||||||| |||||||||||||||| |||||||||||||||| <<<<<<<<<

117583864 gtaaaaacaaaaagtc.tagaataaagcattcc.ttgaattttaaatgaa 117583817

000000251 catgcttttagcagc 000000265

<<<<<<<<< ||| ||||||||||| <<<<<<<<<

117583816 catacttttagcagc 117583802

000000268 ggatacgaagcagactattttcaacaaattgaatgtttc 000000306

<<<<<<<<< | |||||||||||||||| |||||| ||||| ||||||| <<<<<<<<<

117583800 gtatacgaagcagactatcttcaacgaattggatgtttc 117583762

000000310 acttgtgtgagagccaaacaagtgccatacagaaagggacctcttcaatg 000000359

<<<<<<<<< ||||||| |||||||||||||||||||| ||||||||||||||||||||| <<<<<<<<<

117583759 acttgtgcgagagccaaacaagtgccatgcagaaagggacctcttcaatg 117583710

000000360 ttaccnaaagctttgtggcagcatttaagtgtggtggctccatagaagtc 000000409

<<<<<<<<< ||||| |||||||||||||||||||||||||||||||||||||||||||| <<<<<<<<<

117583709 ttacc.aaagctttgtggcagcatttaagtgtggtggctccatagaagtc 117583661

000000410 actatagcatggacgagccacttggcttgct 000000440

<<<<<<<<< ||||||||||||||||||||||||||||||| <<<<<<<<<

117583660 actatagcatggacgagccacttggcttgct 117583630

| Probe Sequence(5'-3') | Probe X | Probe Y | Probe Interrogation Position | Target Strandedness |
| --- | --- | --- | --- | --- |
| CACTGAATTTTCTCTTAGGCTGACC | 839 | 313 | 906 | Antisense |
| TTCTCTTAGGCTGACCATATATTTT | 975 | 1135 | 915 | Antisense |
| ATGTTTTAGACCTCAATGTAAGCTG | 268 | 61 | 981 | Antisense |
| TTCTCTTATCTGTATCATGACATGA | 851 | 1135 | 1063 | Antisense |
| GGATACGAAGCAGACTATTTTCAAC | 1128 | 835 | 1173 | Antisense |
| GTGAGAGCCAAACAAGTGCCATACA | 931 | 771 | 1221 | Antisense |
| GTGCCATACAGAAAGGGACCTCTTC | 424 | 761 | 1236 | Antisense |
| TGTGGCAGCATTTAAGTGTGGTGGC | 338 | 915 | 1278 | Antisense |
| GTGTGGTGGCTCCATAGAAGTCACT | 552 | 783 | 1293 | Antisense |
| GAAGTCACTATAGCATGGACGAGCC | 939 | 539 | 1309 | Antisense |
| GCATGGACGAGCCACTTGGCTTGCT | 423 | 525 | 1321 | Antisense |

**>HG-U133_PLUS_2:202746_AT GENE ITM2A**

aaactactaaccactgcaagctcttgtcaaattttagtttaattggcattgcttgttttttgaaactgaaattacntgagtttcattttttctttgaatttatagggtttagatttctgaaagcagcatgaatatatcacctaacatcctgacaataaattccatccgttgttttttttgtttgtttgttttttcttttcctttaagtaagctctttattcatcttatggtgcagcaattttaaaatttgaaatattttaaattgtttttgaactttttgtgtaaaatatatcagatctcaacattgttggtttcttttgtttttcattttgtacaactttcttgaatttagaaattacatctttgcagttctgttaggtgctctgtaattaacctgacttatatgtgaacaattttcatgagacagtcatttttaactaatgcagtgattctttctcactactatctgtattgtggaatgcacaaaattgtgtaggtgctgaatgctgtaaggagtttaggttgtatgaattctacaaccctataata

**Alignment (orangutan on top; human on bottom)**

00000001 aaactactaaccactgcaagctcttgtcaaattttagtttaattggcatt 00000050

<<<<<<<< |||||||||||||||||||||||||||||||||||||||||||||||||| <<<<<<<<

77133569 aaactactaaccactgcaagctcttgtcaaattttagtttaattggcatt 77133520

00000051 gcttgttttttgaaactgaaattacntgagtttcattttttctttgaatt 00000100

<<<<<<<< |||| |||||||||||||||||||| ||| |||||||||||||||||||| <<<<<<<<

77133519 gcttattttttgaaactgaaattacatgattttcattttttctttgaatt 77133470

00000101 tatagggtttagatttctgaaagcagcatgaatatatcacctaacatcct 00000150

<<<<<<<< ||||||||||||||||||||||||||||||||||| |||||||||||||| <<<<<<<<

77133469 tatagggtttagatttctgaaagcagcatgaatatgtcacctaacatcct 77133420

00000151 gacaataaattccatccgttgttttt.....tttgtttgtttgttttttc 00000195

<<<<<<<< |||||||||||||||||||||||||| ||||||||||||||||||| <<<<<<<<

77133419 gacaataaattccatccgttgtttttttttgtttgtttgtttgttttttc 77133370

00000196 ttttcctttaagtaagctctttattcatcttatggtgcagcaattttaaa 00000245

<<<<<<<< ||||||||||||||||||||||||||||||||||||| |||||||||||| <<<<<<<<

77133369 ttttcctttaagtaagctctttattcatcttatggtggagcaattttaaa 77133320

00000246 atttgaaatattttaaattgtttttgaactttttgtgtaaaatatatcag 00000295

<<<<<<<< |||||||||||||||||||||||||||||||||||||||||||||||||| <<<<<<<<

77133319 atttgaaatattttaaattgtttttgaactttttgtgtaaaatatatcag 77133270

00000296 atctcaacattgttggtttcttttgtttttcattttgtacaactttcttg 00000345

<<<<<<<< |||||||||||||||||| ||||||||||||||||||||||||||||||| <<<<<<<<

77133269 atctcaacattgttggttgcttttgtttttcattttgtacaactttcttg 77133220

00000346 aatttagaaattacatctttgcagttctgttaggtgctctgtaattaacc 00000395

<<<<<<<< |||||||||||||||||||||||||||| ||||||||||||||||||||| <<<<<<<<

77133219 aatttagaaattacatctttgcagttctattaggtgctctgtaattaacc 77133170

00000396 tgacttatatgtgaacaattttcatgagacagtcatttttaactaatgca 00000445

<<<<<<<< |||||||||||||||||||||||||||||||||||||||||||||||||| <<<<<<<<

77133169 tgacttatatgtgaacaattttcatgagacagtcatttttaactaatgca 77133120

00000446 gtgattctttctcactactatctgtattgtggaatgcacaaaattgtgta 00000495

<<<<<<<< |||||||||||||||||||||||||||||||||||||||||||||||||| <<<<<<<<

77133119 gtgattctttctcactactatctgtattgtggaatgcacaaaattgtgta 77133070

00000496 ggtgctgaatgctgtaaggagtttaggttgtatgaattctacaaccctat 00000545

<<<<<<<< |||||||||||||||||||||||||||||||||||||||||||||||||| <<<<<<<<

77133069 ggtgctgaatgctgtaaggagtttaggttgtatgaattctacaaccctat 77133020

00000546 aata 00000549

<<<<<<<< |||| <<<<<<<<

77133019 aata 77133016

| Probe Sequence(5'-3') | Probe X | Probe Y | Probe Interrogation Position | Target Strandedness |
| --- | --- | --- | --- | --- |
| AAACTACTAACCACTGCAAGCTCTT | 460 | 217 | 1283 | Antisense |
| GCAAGCTCTTGTCAAATTTTAGTTT | 294 | 543 | 1298 | Antisense |
| ATTGGCATTGCTTGTTTTTTGAAAC | 251 | 3 | 1324 | Antisense |
| GAATATATCACCTAACATCCTGACA | 505 | 551 | 1412 | Antisense |
| CATCCTGACAATAAATTCCATCCGT | 222 | 347 | 1427 | Antisense |
| AAGCTCTTTATTCATCTTATGGTGC | 675 | 247 | 1491 | Antisense |
| GAAATTACATCTTTGCAGTTCTGTT | 652 | 597 | 1634 | Antisense |
| TTGCAGTTCTGTTAGGTGCTCTGTA | 1118 | 1149 | 1646 | Antisense |
| GTGCTCTGTAATTAACCTGACTTAT | 415 | 765 | 1661 | Antisense |
| GATTCTTTCTCACTACTATCTGTAT | 1128 | 691 | 1730 | Antisense |
| GTATGAATTCTACAACCCTATAATA | 618 | 725 | 1807 | Antisense |

**>HG-U133_PLUS_2:207644_AT GENE FOXH1**

ttcctggccccacgagagtggagggggagactgtgcaggggggagccatcgggccctcaaccctctccccagagcctagggcctggcctctccacttactgcagggcaccgcagttcctgggggacggtccagcgggggacacagggcctccctctgggggcagctgcccacctcctacttgcctatctacactcccaatgtggtaatgcccttggcaccaccacccacctcctgtccccagtgtccgtcaaccagccctgcctactggggggtggcccctgaaacccgagggcccccagggctgctctgcgatctagacgccctcttccaaggggtgccacccaacaaaagcatctacgacgtttgggtcagccaccctcgggacctggcggcccctggcccaggctggctgctctcctggtgcagcctgtgaggctcttaagacaggggccgctc

**Alignment (orangutan on top; human on bottom)**

000000001 ttcctggccccacgagagtggagggggagactgtgcaggggggagccatc 000000050

<<<<<<<<< |||||| |||||| |||||||||||||||||| ||||||||||||||||| <<<<<<<<<

152835723 ttcctgtccccaccagagtggagggggagactctgcaggggggagccatc 152835674

000000051 gggccctcaaccctctccccagagcctagggcctggcctctccacttact 000000100

<<<<<<<<< ||||||| ||||||||||||||||||||||||||||||||||||||||| <<<<<<<<<

152835673 aggccctcgaccctctccccagagcctagggcctggcctctccacttact 152835624

000000101 gcagggcaccgcagttcctgggggacggtccagcgggggacacagggcct 000000150

<<<<<<<<< |||||||||| |||||||| |||||| ||||||||||||||||||||||| <<<<<<<<<

152835623 gcagggcacctcagttcctaggggactgtccagcgggggacacagggcct 152835574

000000151 ccctctgggggcagctgcccacctcctacttgcctatctacactcccaat 000000200

<<<<<<<<< |||||||||||||||||||||||||||||||||||||||||||||||||| <<<<<<<<<

152835573 ccctctgggggcagctgcccacctcctacttgcctatctacactcccaat 152835524

000000201 gtggtaatgcccttggcaccaccacccacctcctgtccccagtgt...cc 000000247

<<<<<<<<< |||||||||||||||||||||||||||||||||||||||| |||| || <<<<<<<<<

152835523 gtggtaatgcccttggcaccaccacccacctcctgtccccggtgtccacc 152835474

000000248 gtcaaccagccctgcctactggggggtggcccctgaaacccgagggcccc 000000297

<<<<<<<<< |||||||||||||||||||||||| ||| ||||||||||||||||||||| <<<<<<<<<

152835473 gtcaaccagccctgcctactggggcgtgacccctgaaacccgagggcccc 152835424

000000298 cagggctgctctgcgatctagacgccctcttccaaggggtgccacccaac 000000347

<<<<<<<<< |||||||||||||||||||||||||||||||||| ||||||||||||||| <<<<<<<<<

152835423 cagggctgctctgcgatctagacgccctcttccagggggtgccacccaac 152835374

000000348 aaaagcatctacgacgtttgggtcagccaccctcgggacctggcggcccc 000000397

<<<<<<<<< |||||||||||||||||||||||||||||||||||||||||||| ||||| <<<<<<<<<

152835373 aaaagcatctacgacgtttgggtcagccaccctcgggacctggcagcccc 152835324

000000398 tggcccaggctggctgctctcctggtgcagcctgtgaggctcttaagaca 000000447

<<<<<<<<< |||||||||||||||||||||||||||||||||||||||||| ||||||| <<<<<<<<<

152835323 tggcccaggctggctgctctcctggtgcagcctgtgaggctcctaagaca 152835274

000000448 ggggccgctc 000000457

<<<<<<<<< |||||||||| <<<<<<<<<

152835273 ggggccgctc 152835264

| Probe Sequence(5'-3') | Probe X | Probe Y | Probe Interrogation Position | Target Strandedness |
| --- | --- | --- | --- | --- |
| TTCCTGGCCCCACGAGAGTGGAGGG | 151 | 1143 | 1256 | Antisense |
| CCACGAGAGTGGAGGGGGAGACTGT | 80 | 463 | 1265 | Antisense |
| CCTATCTACACTCCCAATGTGGTAA | 597 | 449 | 1438 | Antisense |
| TACACTCCCAATGTGGTAATGCCCT | 781 | 1051 | 1444 | Antisense |
| GGTGCCACCCAACAAAAGCATCTAC | 461 | 821 | 1590 | Antisense |
| GCCACCCAACAAAAGCATCTACGAC | 762 | 471 | 1593 | Antisense |
| AGCATCTACGACGTTTGGGTCAGCC | 1087 | 133 | 1606 | Antisense |
| CATCTACGACGTTTGGGTCAGCCAC | 284 | 347 | 1608 | Antisense |
| CCTGGTGCAGCCTGTGAGGCTCTTA | 722 | 439 | 1673 | Antisense |
| GTGCAGCCTGTGAGGCTCTTAAGAC | 1057 | 765 | 1677 | Antisense |
| GAGGCTCTTAAGACAGGGGCCGCTC | 213 | 661 | 1688 | Antisense |

**>HG-U133_PLUS_2:206268_AT GENE LEFTY1**

gccttcaagtggccgtttctggggcctcgacagtgcatcgcctcggagactgactcgctgcccatgatcgtcagcatcaaggagggaggcaggaccaggccccaggtggtcagcctgcccaacatgagggtgcagaagtgcagctgtgcctcggatggtgcgctcgtgccaaggaggctccagccataggcgcctagtgtagccatcgagggacttgacttgtgtgtgtttctgaagtgttcgagggtaccaggagagctggcgatgactgaactgctgatggacaaatgctctgtgctctctagtgagccctgaatttgcttcctctgacaagttacctcacctaatttttgcttctcaggaatgagaatctttggccactggagagcccttgctcagttttc

#### Alignment (orangutan on top; human on bottom)

00000001 gccttcaagtggccgtttctggggcctcgacagtgcatcgcctcggagac 00000050

>>>>>>>> |||||||||||||||||||||||||| ||||||||||||||||||||||| >>>>>>>>

23781667 gccttcaagtggccgtttctggggccgcgacagtgcatcgcctcggagac 23781716

00000051 tgactcgctgcccatgatcgtcagcatcaaggagggaggcaggaccaggc 00000100

>>>>>>>> || ||||||||||||||||||| ||||||||||||||||||||| ||||| >>>>>>>>

23781717 tgcctcgctgcccatgatcgtcggcatcaaggagggaggcaggatcaggc 23781766

00000101 cccaggtggtcagcctgcccaacatgagggtgcagaagtgcagctgtgcc 00000150

>>>>>>>> |||||||||||||||||||||||||||||||||||||||||||||||||| >>>>>>>>

23781767 cccaggtggtcagcctgcccaacatgagggtgcagaagtgcagctgtgcc 23781816

00000151 tcggatggtgcgctcgtgccaaggaggctccagccataggcgcctagtgt 00000200

>>>>>>>> |||||||| |||||||||||||||||||||||||||||||||||||| || >>>>>>>>

23781817 tcggatggggcgctcgtgccaaggaggctccagccataggcgcctagagt 23781866

00000201 agccatcgagggacttgacttgtgtgtgtttctgaagtgttcgagggtac 00000250

>>>>>>>> |||||||||||||||||||||||||||||||||||||||||| ||||||| >>>>>>>>

23781867 agccatcgagggacttgacttgtgtgtgtttctgaagtgttctagggtac 23781916

00000251 caggagagctggcgatgactgaactgctgatggacaaatgctctgtgctc 00000300

>>>>>>>> |||||||||||| ||||||||| ||||||||||||||||||| ||||||| >>>>>>>>

23781917 caggagagctggtgatgactgagctgctgatggacaaatgctgtgtgctc 23781966

00000301 tctagtgagccctgaatttgcttcctctgacaagttacctcacctaattt 00000350

>>>>>>>> |||||||||||||||||||||||||||||||||||||||||||||||||| >>>>>>>>

23781967 tctagtgagccctgaatttgcttcctctgacaagttacctcacctaattt 23782016

00000351 ttgcttctcaggaatgagaatctttggccactggagagcccttgctcagt 00000400

>>>>>>>> |||||||||||||||||||||| ||||||||||||||||||||||||||| >>>>>>>>

23782017 ttgcttctcaggaatgagaatccttggccactggagagcccttgctcagt 23782066

00000401 tttc 00000404

>>>>>>>> |||| >>>>>>>>

23782067 tttc 23782070

| Probe Sequence(5'-3') | Probe X | Probe Y | Probe Interrogation Position | Target Strandedness |
| --- | --- | --- | --- | --- |
| GCCTTCAAGTGGCCGTTTCTGGGGC | 1086 | 475 | 995 | Antisense |
| GCCATAGGCGCCTAGTGTAGCCATC | 338 | 475 | 1177 | Antisense |
| GTAGCCATCGAGGGACTTGACTTGT | 375 | 731 | 1193 | Antisense |
| AGTGTTCGAGGGTACCAGGAGAGCT | 170 | 93 | 1230 | Antisense |
| GGCGATGACTGAACTGCTGATGGAC | 1031 | 883 | 1255 | Antisense |
| ACAAATGCTCTGTGCTCTCTAGTGA | 808 | 193 | 1278 | Antisense |
| TAGTGAGCCCTGAATTTGCTTCCTC | 154 | 1073 | 1297 | Antisense |
| ATTTGCTTCCTCTGACAAGTTACCT | 320 | 19 | 1310 | Antisense |
| CCTCACCTAATTTTTGCTTCTCAGG | 442 | 447 | 1332 | Antisense |
| GAATGAGAATCTTTGGCCACTGGAG | 452 | 557 | 1356 | Antisense |
| ACTGGAGAGCCCTTGCTCAGTTTTC | 599 | 165 | 1374 | Antisense |

**>HG-U133_PLUS_2:205850_S_AT GENE GABRB3**

Gcaaccgggtggatgctcatggaaatattctgttgacatcgctggaagttcacaatgaaatgaatgaggtctcaggcggcattggcgataccaggaattcagcaatatcctttgacaactcaggaatccagtacaggaaacagagcatgcctcgagaagggcatgggcgattcctgggggacagaagcctcccgcacaagaagacccatctacggaggaggtcttcacagctcaaaattaaaatacctgatctaaccgatgtgaatgccatagacagatggtccaggatcgtgtttccattcactttttctcttttcaacttagtttactggctgtactatgttaactgag

**Alignment (orangutan on top; human on bottom)**

00000008 ggtggatgctcatggaaatattctgttgacatcgctggaagttcacaatg 00000057

<<<<<<<< |||||||||||||||||||||||||||||||||||| ||||||||||||| <<<<<<<<

22833683 ggtggatgctcatggaaatattctgttgacatcgctcgaagttcacaatg 22833634

00000058 aaatgaatgaggtctcaggcggcattggcgataccaggaattcagcaata 00000107

<<<<<<<< |||||||||||||| |||| || |||||||||||||||||||||||||| <<<<<<<<

22833633 aaatgaatgaggtcgcaggtggtgttggcgataccaggaattcagcaata 22833584

00000108 tcctttgacaactcaggaatccagtacaggaaacagagcatgcctcgaga 00000157

<<<<<<<< |||||||||||||||||||||||||||||||||||||||||||||||||| <<<<<<<<

22833583 tcctttgacaactcaggaatccagtacaggaaacagagcatgcctcgaga 22833534

00000158 agggcatgggcgattcctgggggacagaagcctcccgcacaagaagaccc 00000207

<<<<<<<< |||||||||||||||||||||||||||||||||||||||||||||||||| <<<<<<<<

22833533 agggcatgggcgattcctgggggacagaagcctcccgcacaagaagaccc 22833484

00000208 atctacggaggaggtcttcacagctcaaaattaaaatacctgatctaacc 00000257

<<<<<<<< ||||||||||||||||||| |||||||||||||||||||||||||||||| <<<<<<<<

22833483 atctacggaggaggtcttcgcagctcaaaattaaaatacctgatctaacc 22833434

00000258 gatgtgaatgccatagacagatggtccaggatcgtgtttccattcacttt 00000307

<<<<<<<< |||||||||||||||||||||||||||||||||||||||||||||||||| <<<<<<<<

22833433 gatgtgaatgccatagacagatggtccaggatcgtgtttccattcacttt 22833384

00000308 ttctcttttcaacttagtttactggctgtactatgttaactgag 00000351

<<<<<<<< |||||||||||||||||||||||||||||||||||||||||||| <<<<<<<<

22833383 ttctcttttcaacttagtttactggctgtactatgttaactgag 22833340

| Probe Sequence(5'-3') | Probe X | Probe Y | Probe Interrogation Position | Target Strandedness |
| --- | --- | --- | --- | --- |
| GCAACCGGGTGGATGCTCATGGAAA * | 40 | 545 | 1224 | Antisense |
| TCTGTTGACATCGCTGGAAGTTCAC | 791 | 999 | 1252 | Antisense |
| TCTCAGGCGGCATTGGCGATACCAG | 1049 | 1005 | 1293 | Antisense |
| TCAGCAATATCCTTTGACAACTCAG | 912 | 1023 | 1322 | Antisense |
| GCCTCGAGAAGGGCATGGGCGATTC | 1062 | 479 | 1372 | Antisense |
| GGGCGATTCCTGGGGGACAGAAGCC | 1024 | 789 | 1388 | Antisense |
| CAAGAAGACCCATCTACGGAGGAGG | 854 | 293 | 1420 | Antisense |
| ACGGAGGAGGTCTTCACAGCTCAAA | 631 | 163 | 1435 | Antisense |
| AAATACCTGATCTAACCGATGTGAA | 983 | 199 | 1464 | Antisense |
| AGACAGATGGTCCAGGATCGTGTTT | 374 | 121 | 1495 | Antisense |
| TACTGGCTGTACTATGTTAACTGAG | 145 | 1059 | 1550 | Antisense |

*Beginning of Probe Sequence not present in side by side alignment.

**>HG-U133_PLUS_2:204466_S_AT GENE SNCA**

gaaatatctttgctcccagtttcttgagatctgctgacagatgttccatcctgtacaagtgctcagttccaatgtgcccagtcatgacatttctcaaagtttttacagtgtatctcgaagtcttccatcagcagtgattgaagtatctgtacctgcccccactcagcatttcggtgcttccctttcactgaagtgaatacatggtagcagggtctttgtgtgctgtggattttgtggcttcaatctacgatgttaaaacaaattaaaaacacctaagtgactaccacttatttctaaatc

**Alignment (orangutan on top; human on bottom)**

00000001 gaaatatctttgctcccagtttcttgagatctgctgacagatgttccatc 00000050

<<<<<<<< ||||||||||||||||||||||| ||||||||||||||||| |||||||| <<<<<<<<

93673874 gaaatatctttgctcccagtttcatgagatctgctgacagacgttccatc 93673825

00000051 ctgtacaagtgctcagttccaatgtgcccagtcatgacatttctcaaagt 00000100

<<<<<<<< ||||||||||| |||||||||||||||||||||||||||||||||||||| <<<<<<<<

93673824 ctgtacaagtggtcagttccaatgtgcccagtcatgacatttctcaaagt 93673775

00000101 ttttacagtgtatctcgaagtcttccatcagcagtgattgaagtatctgt 00000150

<<<<<<<< ||||||||||||||| ||||||||||||||||| |||||||||||||||| <<<<<<<<

93673774 ttttacagtgtatcttgaagtcttccatcagcaatgattgaagtatctgt 93673725

00000151 acctgcccccactcagcatttcggtgcttccctttcactgaagtgaatac 00000200

<<<<<<<< |||||||||||||||||||||||||||||||||||||||||||||||||| <<<<<<<<

93673724 acctgcccccactcagcatttcggtgcttccctttcactgaagtgaatac 93673675

00000201 atggtagcagggtctttgtgtgctgtggattttgtggcttcaatctacga 00000250

<<<<<<<< ||||||||||||||||||||||||||||||||||||||||||||||| || <<<<<<<<

93673674 atggtagcagggtctttgtgtgctgtggattttgtggcttcaatctatga 93673625

00000251 tgttaaaacaaattaaaaacacctaagtgactaccacttatttctaaatc 00000300

<<<<<<<< |||||||||||||||||||||||||||||||||||||||||||||||||| <<<<<<<<

93673624 tgttaaaacaaattaaaaacacctaagtgactaccacttatttctaaatc 93673575

| Probe Sequence(5'-3') | Probe X | Probe Y | Probe Interrogation Position | Target Strandedness |
| --- | --- | --- | --- | --- |
| GAAATATCTTTGCTCCCAGTTTCTT | 71 | 597 | 699 | Antisense |
| ATCTGCTGACAGATGTTCCATCCTG | 439 | 45 | 727 | Antisense |
| GTTCCATCCTGTACAAGTGCTCAGT | 939 | 701 | 741 | Antisense |
| ACAAGTGCTCAGTTCCAATGTGCCC | 43 | 197 | 753 | Antisense |
| TGTGCCCAGTCATGACATTTCTCAA | 304 | 921 | 771 | Antisense |
| TACAGTGTATCTCGAAGTCTTCCAT | 128 | 1053 | 802 | Antisense |
| GAAGTCTTCCATCAGCAGTGATTGA | 74 | 565 | 815 | Antisense |
| TGATTGAAGTATCTGTACCTGCCCC | 1037 | 933 | 833 | Antisense |
| GGTAGCAGGGTCTTTGTGTGCTGTG | 1029 | 827 | 901 | Antisense |
| ATTTTGTGGCTTCAATCTACGATGT | 594 | 15 | 927 | Antisense |
| AGTGACTACCACTTATTTCTAAATC | 758 | 93 | 974 | Antisense |

**>HG-U133_PLUS_2:204271_S_AT GENE EDNRB**

aactgctttaagtcatgcttatgctgctggtgccagtcatttgaagaaaaacagtccttggaggaaaagcagtcgtgcttaaagttcaaagctaatgatcacggatatgacaacttccgttccagtaataaatacagctcatcttgaaa

**Alignment (orangutan on top; human on bottom)**

00000011 agtcatgcttatgctgctggtgccagtcatttgaagaaaaacagtccttg 00000060

<<<<<<<< |||||||||||||||||||||||||||||||||||||||||||||||||| <<<<<<<<

79323111 agtcatgcttatgctgctggtgccagtcatttgaagaaaaacagtccttg 79323062

00000061 gaggaaaagcagtcgtgcttaaagttcaaagctaatgatcacggatatga 00000110

<<<<<<<< |||||||||||||||||||||||||||||||||||||||||||||||||| <<<<<<<<

79323061 gaggaaaagcagtcgtgcttaaagttcaaagctaatgatcacggatatga 79323012

00000111 caacttccgttccagtaataaatacagctcatcttgaaa 00000149

<<<<<<<< ||||||||||||||||||||||||||||||||||||||| <<<<<<<<

79323011 caacttccgttccagtaataaatacagctcatcttgaaa 79322973

| Probe Sequence(5'-3') | Probe X | Probe Y | Probe Interrogation Position | Target Strandedness |
| --- | --- | --- | --- | --- |
| AACTGCTTTAAGTCATGCTTATGCT * | 749 | 237 | 1241 | Antisense |
| AAGTCATGCTTATGCTGCTGGTGCC * | 1122 | 259 | 1250 | Antisense |
| TCATGCTTATGCTGCTGGTGCCAGT | 200 | 1019 | 1253 | Antisense |
| GCTTATGCTGCTGGTGCCAGTCATT | 409 | 515 | 1257 | Antisense |
| TGCTGCTGGTGCCAGTCATTTGAAG | 175 | 965 | 1262 | Antisense |
| GTCCTTGGAGGAAAAGCAGTCGTGC | 983 | 759 | 1294 | Antisense |
| GCAGTCGTGCTTAAAGTTCAAAGCT | 1009 | 527 | 1309 | Antisense |
| GTTCAAAGCTAATGATCACGGATAT | 1062 | 705 | 1324 | Antisense |
| GGATATGACAACTTCCGTTCCAGTA | 164 | 837 | 1343 | Antisense |
| GACAACTTCCGTTCCAGTAATAAAT | 386 | 589 | 1349 | Antisense |
| GTAATAAATACAGCTCATCTTGAAA | 397 | 741 | 1365 | Antisense |

*Note that beginning of Probe Sequence is not present in side by side alignment.

**Key:**

**Yellow highlight with asterisk*** indicates truncated Probe Sequence.

**Yellow highlight** indicates Probe Sequence with insertions or deletions.

**Red highlight** indicates total Probe Sequence not contained.

**Green highlight** indicates complete match.

**Red font text** indicates mismatch.

**Blue text** indicates overlapping sequences.
