# Supplementary figures and images for: Derivation of induced pluripotent stem cells from orangutan skin fibroblasts
Source: BMC Res Notes. 2015 Oct 16;8:577. doi: 10.1186/s13104-015-1567-0 (PMC4609060; doi:10.1186/s13104-015-1567-0)

## Slide 1
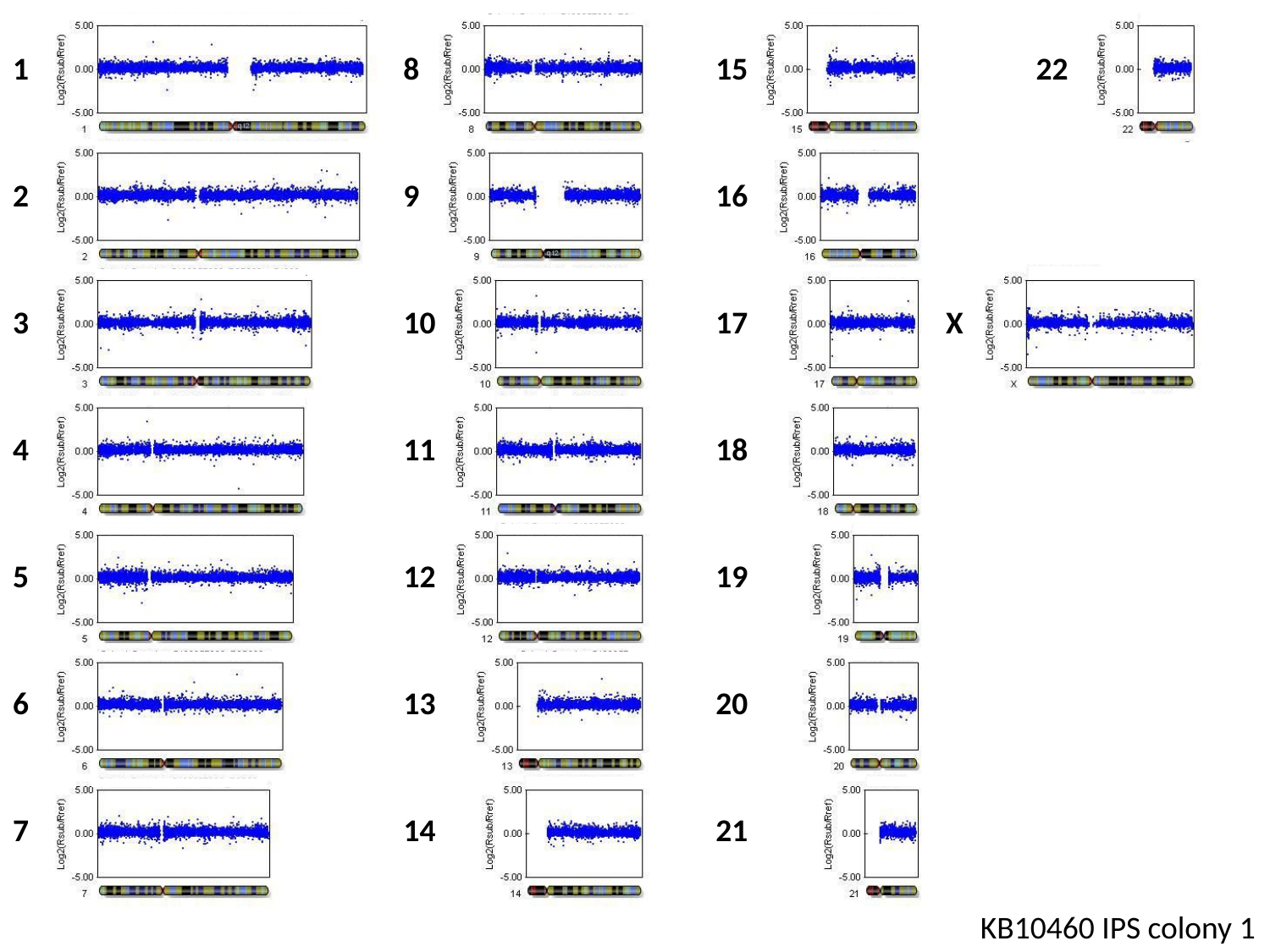

1
8
15
22
2
9
16
3
10
17
X
4
11
18
5
12
19
6
13
20
7
14
21
KB10460 IPS colony 1

## Slide 2
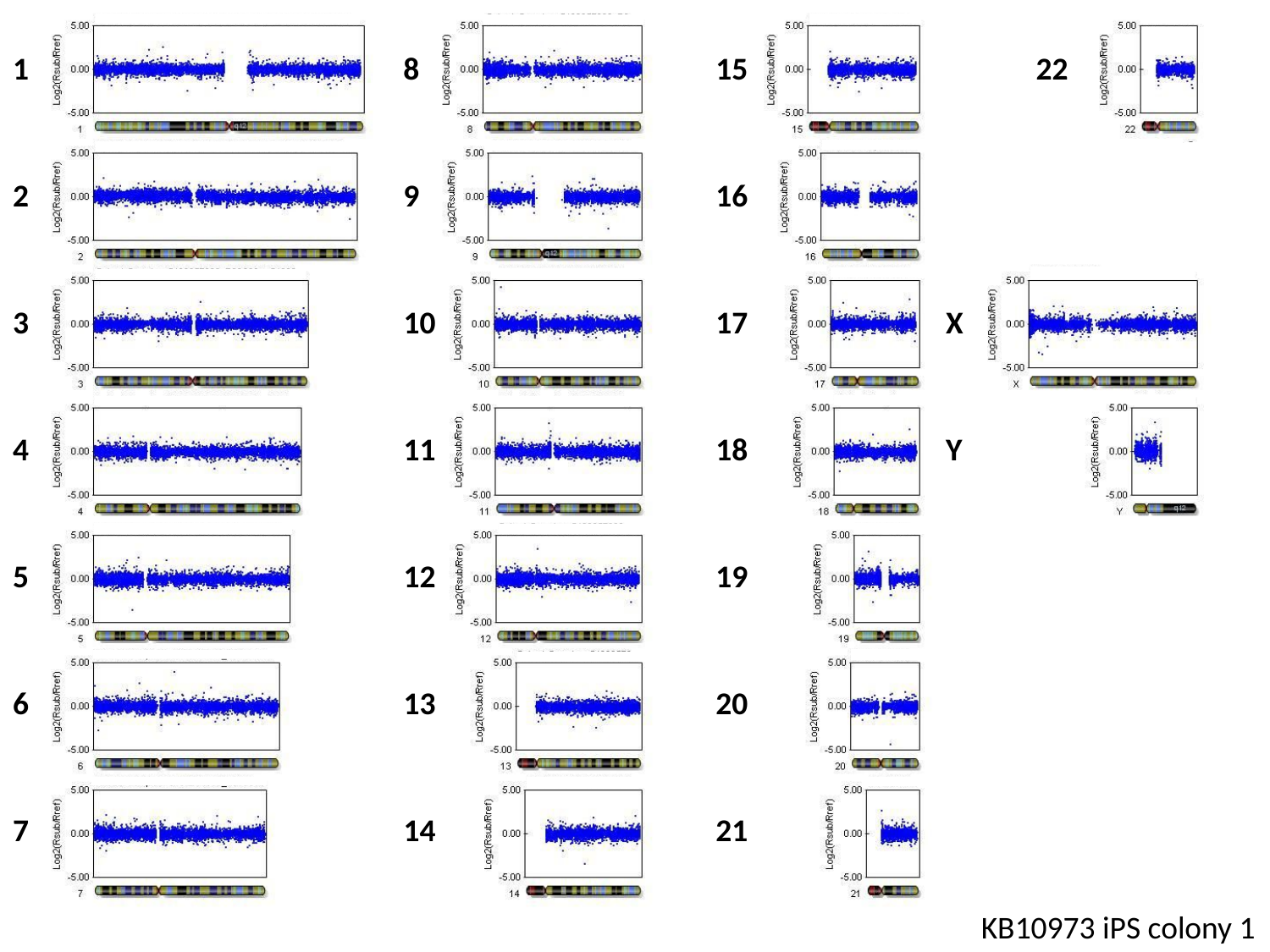

1
8
15
22
2
9
16
3
10
17
X
4
11
18
Y
5
12
19
6
13
20
7
14
21
KB10973 iPS colony 1

Supplement: Supplementary file 3 — 10.1186/s13104-015-1567-0 Copy number variation analysis for orangutan iPSCs. Log base 2 of the ratio of subject (iPSC) and reference (fibroblast) R values for probes in the CNV BeadArray are provided for KB10973 iPSC colony 1 and KB10460 iPSC colony 1. All data is mapped onto the human karyotype. [file 13104_2015_1567_MOESM3_ESM.pptx]
